# A 3D braincase of the early jawed vertebrate *Palaeospondylus* from Australia

**DOI:** 10.1093/nsr/nwae444

**Published:** 2024-12-03

**Authors:** Carole J Burrow, Gavin C Young, Jing Lu

**Affiliations:** Geosciences, Queensland Museum, Hendra 4011, Australia; Department of Materials Physics, Australian National University, Canberra 2600, Australia; Australian Museum Research Institute, Australian Museum, Sydney 2010, Australia; Key Laboratory of Vertebrate Evolution and Human Origins of Chinese Academy of Sciences, Institute of Vertebrate Paleontology and Paleoanthropology, Beijing 100044, China; University of Chinese Academy of Sciences, Beijing 100049, China

**Keywords:** Australia, Early Devonian, gnathostome, *Palaeospondylus*, braincase

## Abstract

*Palaeospondylus gunni* Traquair, 1890, is represented by thousands of similarly preserved articulated fossils from the Achannaras quarry (∼390 Mya) in Caithness, Scotland. With radically different interpretations of its structure, it has been assigned to almost all major jawless and jawed vertebrate groups. Here we report a new and older species of *Palaeospondylus* from the Early Devonian of Australia (c. 400 Ma), investigated using high-resolution computed tomography. Its 3D-preserved braincase demonstrates a combination of primitive gnathostome features including an anteriorly positioned transverse cranial fissure of uncertain homology, a large dorsal fontanelle and a small hypophysial fossa. Contrary to recent interpretations of *P. gunni*, the new braincase shows that *Palaeospondylus* lacks both a postorbital process and an intracranial joint. Our new Australian species extends the history of *Palaeospondylus* back some 10 million years prior to its occurrence in Scotland. The newly identified neurocranial characters have been coded into a phylogenetic analysis that places *Palaeospondylus* as a sister group of the Chondrichthyes, but some neurocranial characters could indicate a phylogenetic position within the gnathostome stem group.

## Teaser

This paper describes a new Australian species of an enigmatic 400-million-year-old fossil vertebrate, Palaeospondylus, previously only known from Scotland. The 3D preservation of the new specimens facilitates a much better understanding of the skeletal anatomy of this unusual jawed vertebrate.

## INTRODUCTION

Nearly all of the thousands of specimens of the enigmatic early gnathostome *Palaeospondylus gunni* come from its type locality (the Achannaras flagstone quarry). Despite this abundance, its affinities to other vertebrates have been widely debated over the last 130+ years [1–5]. Recent work based on computed tomography and synchrotron scanning has not resolved its affinities, with *Palaeospondylus* assessed as either related to chondrichthyans [6] or even as a ‘stem-tetrapod’ [7,8]. Until now, *Palaeospondylus* has been known only from the Middle Devonian Orcadian Basin of Scotland [9]. It was initially interpreted as a jawless vertebrate [10], and shortly after placed in its own order and family [11]. Whereas the Scottish specimens are extremely compressed dorsoventrally, with all skeletal elements welded together, our new discovery of *Palaeospondylus*, in a 400-million-year-old limestone of central Australia, has a completely different preservation as 3D uncrushed disarticulated elements. These display the distinctive histology of large cartilage cell spaces as inferred for the bituminized Scottish *Palaeospondylus* [4]. Our 3D-preserved isolated neurocranium shares unique features with the Scottish *P. gunni*, and for the first time provides undistorted details of the neurocranium. It demonstrates that various structures, previously misinterpreted as neurocranial, are separate elements of the jaws and visceral arches. The controversial recent proposal of tetrapod affinities [7,8,12], implicating *Palaeospondylus* in perhaps the most significant ecological transition in vertebrate evolutionary history (from the aquatic to the terrestrial environment), is refuted by our new evidence. The new *Palaeospondylus* from central Australia clarifies the neurocranial morphology of this enigmatic animal—a major contribution to resolving the phylogenetic affinity of *Palaeospondylus*. A much wider and earlier distribution than previously assumed is indicated, given the two known localities were on opposite sides of the globe in Early Devonian times.

## RESULTS

### Systematic palaeontology

Gnathostomata Gegenbaur, 1874

*Palaeospondylus* Traquair, 1890

**Diagnosis.** Gnathostome lacking a dermal skeleton; endoskeletal tissues composed of a honeycomb-like homogeneous mineralized matrix surrounding large cartilage cell spaces comprise the neurocranium, occipital arches, visceral and mandibular arches, rostral complex, nasal capsules, postoccipital lamellae, vertebral centra, neural arches and fin rays. An elongate dorsal fontanelle comprises at least half the preserved braincase length. The narrow pre-otic region of the braincase forms a prominent T-shaped ventral thickening. A transverse cranial fissure divides the braincase into pre- and postorbital regions. Paired occipital arches are separated from the neurocranium by an oticoccipital fissure. The hyoid arch comprises three elements; the proximal element is short and square with a canal presumably for the facial nerve, a middle element is ‘dog-bone’ shaped, and the ceratohyal is a large broad flat element. Vertebral centra are hollow semi-circular elements, with separate neural and haemal arches. Postoccipital lamellae articulate with the posterior of the otic capsule.

#### *Palaeospondylus australis* sp. nov.

**Figure 1.**
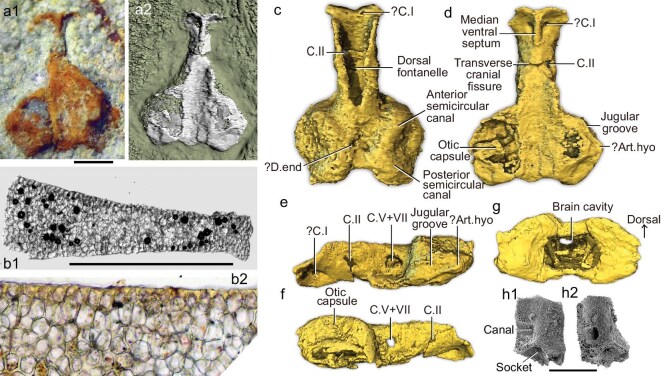
*Palaeospondylus australis* sp. nov. braincase and histological section. (a, c–g) Braincase QMF52826: (a1) light microscope image, (a2) HRCT surface scan; (c–g) HRCT scans in (c) dorsal, (d) ventral, (e) left lateral, (f) right lateral and (g) posterior views. (b) QMF53552, thin section of an undetermined element, (b1) whole element, (b2) closeup. (h) QMF53548, Type 1 element = hyomandibula, (h1) lateral and (h2) anterolateral views. Abbreviations: ?art, possible articulation; ?art.hyo, possible articulation for hyomandibula; c.I, olfactory nerve foramen; c.II, optic nerve foramen; c.V + VII, foramen for trigeminal and facial nerves; d.end, endolymphatic duct opening. Scale bars, 1.0 mm.

**Figure 2.**
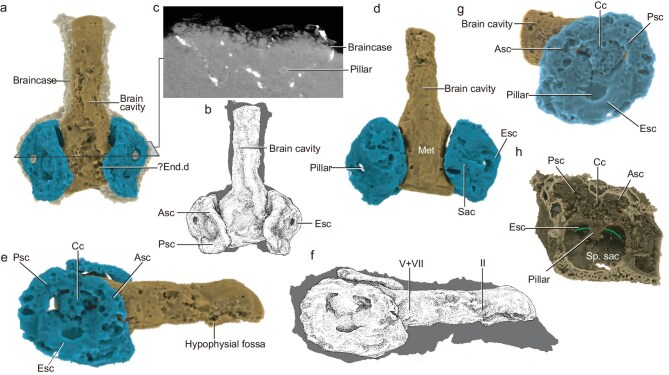
3D Mimics rendered braincase of *P. australis.* (a–b) Dorsal view (b, illustrative drawing). (c) Transverse section showing the pillar separating the external semicircular canal. (d) Ventral view. (e–f) Right lateral view (f, illustrative drawing, approximate positions of II and V + VII indicated). (g) Lateral view of the left side of the otic capsule. (h) Vertical cut-away view of the left otic capsule (anterior to right), showing the pillar separating the external semicircular canal. Abbreviations: asc, anterior semicircular canal; cc, crus commune; end.d, endolymphatic duct; esc, external semicircular canal; met, metencephalon; psc, posterior semicircular canal; sp.sac, space for sacculus; II, canal for optic nerve; V + VII, canal for trigeminal and facial nerves.

**Holotype**. QMF52826, a 3D preserved braincase (Figs 1a, c–g, 2a–h, Figs S2b, S3–5, S6a–d).

**Figure 3.**
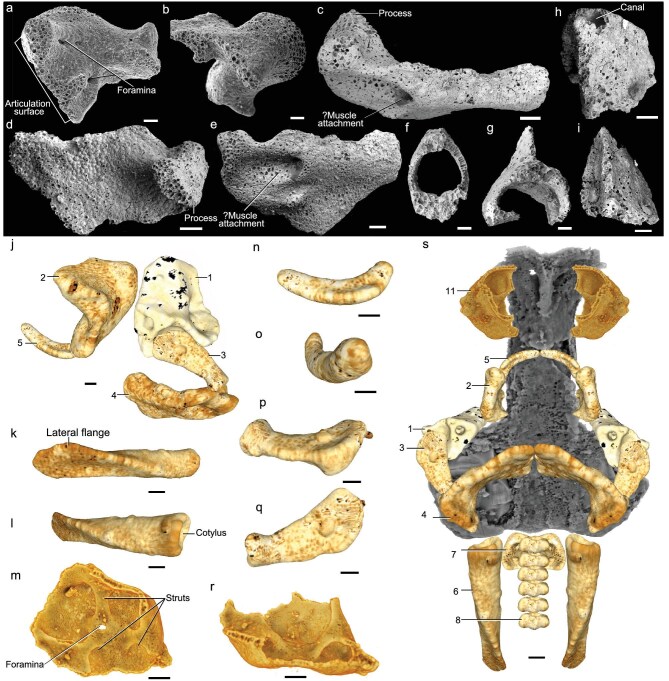
SEM and 3D scan images of *P. australis* sp. nov. isolated elements. (a, b) QMF53547, Type 2 element, possible left palatoquadrate in dorsomedial and dorsolateral views. (c–e) QMF52830, Type 4 element, ceratohyal, in dorsal, ventral and anterior views. (f) QMF52832, Type 8 element, vertebral centrum. (g) QMF52843, Type 9 element, neural or haemal arch with spine. (h, i) QMF52831, Type 7 element, occipital arch, apex and lateral views. (j) Possible arrangement of the left side mandibular and hyoid arch elements QMF53550.1, 3, 4, 5, 9 in lateral view. (k, l) QMF53550.11, Type 6 element, right postoccipital lamella, ventrolateral and dorsolateral views. (m, n) QMF53550.3, Type 5 element, possible Meckel's cartilage, ?anterodorsal and anterolateral views. (o, p) QMF 53550.4, Type 3 element, interhyal (or hyomandibula). (q, r) QMF52828, Type 11 element, ‘hemidome’ sensu Sollas and Sollas [17] (possible nasal capsule), internal view and lateral/anterior view. (s) Reconstruction of the neurocranium (grey) and associated elements as in (j) plus vertebral centra, occipital arches and postoccipital lamellae QMF53550.7, 8, 11, ventral view. Scale bars, 0.1 mm; (a–i) scanning electron micrographs; (j–s) 3D microCT scan images; (j–p) Meshlab images; (q, r, s) Drishti images. Type 1–11 elements, see Supplementary Data and Table S1.

**Referred material**. Two hundred and twenty isolated elements of eleven morphotypes and braincase fragments, plus thin sections, including QMF52827.1-5, 52828.1-5, 52829-32, 52843, 53546-49, 53550.1-11 and 53552 (Figs 1b, h, 3a–r, Table S1, Figs S1a–d, S6e–j, S7).

**Locality and horizon**. A small limestone outcrop of late Emsian age in the Cravens Peak Beds, Georgina Basin, western Queensland, Australia. Approximate coordinates: 23°23'S; 138°08'E (see Supplementary Data for more details).

**Etymology.** The species name *australis* (Latin), ‘southern’, acknowledging that the new species comes from Australia, in the southern hemisphere.

**Diagnosis**. A *Palaeospondylus* with the narrow pre-otic region of the neurocranium slightly longer than the otic region. The T-shaped ventral thickening forms a convex rather than straight or concave anteroventral margin, with a prominent median ventral process. The elongate dorsal fontanelle has a narrow and pointed rather than transverse posterior border. A deep posterior embayment between otic capsules includes a median posterior process projecting from beneath the cranial cavity. Up to five paired articular surfaces on the neurocranium include a palatobasal (possibly double) connection for the palatoquadrate, two surfaces in the otic region possibly representing connections with mandibular or hyoid arch elements, and a posterior articulation for ‘postoccipital lamellae’. Paired pyramid-shaped occipital arches. Anterior and posterior semicircular canals of the labyrinth connect; the lateral semicircular canal is incompletely separated; the saccular space is small and situated in a dorsal position. The jugular vein was not invested in the otic region.

### Description

The braincase and all the other endoskeletal elements assigned to *Palaeospondylus australis* are preserved in 3D (Fig. 1a and c–g). The histological structure of all elements shows a spongiose tissue, with subspherical to polyhedral spaces ca. 30 μm in diameter separated by thin trabeculae (Fig. 1b and Fig. S1a–d). All elements have the same composition, being totally formed of an open network of a mineralized extracellular matrix (ECM). This matrix shows no evidence of bone cell lacunae, fibrous strands or any other evidence of collagen. The same type of structure is otherwise only recorded for *P. gunni* (Fig. S1e and f).

The holotype isolated braincase is preserved with the ventral floor exposed, and the rest embedded in a matrix (Fig. 1a), providing a unique opportunity to investigate its structure unhindered by other endoskeletal elements (see Supplementary Data for details of comparative morphology). Its overall shape closely resembles a restoration of *P. gunni* from over 80 years ago by Moy-Thomas [1], with expanded otic capsules posteriorly, and the cranial cavity open anteriorly and dorsally through a dorsal fontanelle (Fig. S2). The latter is a common feature of embryonic, incompletely chondrified neurocrania [13], so paedomorphic development of the new braincase is suggested. The absence of any change in proportions or morphology with increase in size of the associated elements suggests we have an adult form, not a larva. The same arguments regarding the well-mineralized cranial and axial skeletons of *P. gunni* also convinced Moy-Thomas [1] that it was an adult. The overall neurocranial resemblance (Fig. S2) demonstrates that Moy-Thomas provided the most reliable previous attempt to decide which associated elements in the articulated type species were not neurocranial structures. Thus, the structure interpreted elsewhere [6] as a projecting postorbital process pierced by a jugular canal, and a bulbous ventral projection beneath the otic capsule interpreted as a hyomandibular articular facet [7], were separate elements (gammation (GA), branchial arch (BA), Fig. S2d), rather than parts of the neurocranium. In addition, the pro-otic [7] and ‘lateral otic’ processes [6] of *P. gunni* were accentuated by compression, and form only slight angles on the uncrushed braincase of *Palaeospondylus australis* sp. nov. (Fig. 1c and d). Recent arguments for and against the tetrapodomorph interpretation for *Palaeospondylus* [8,12] were based on these erroneous interpretations.

In dorsal view, the elongate dorsal fontanelle of *P. australis* occupies over 50% of the preserved braincase length (Fig. 1c). The anterior portion recalls the precerebral fontanelle in primitive chondrichthyans, whereas the posterior portion resembles the anterior dorsal fontanelle of actinopterygians, considered to be an osteichthyan synapomorphy [14]. The different shape to the dorsal fontanelle of *P. gunni*, and the longer pre-otic region of our braincase restoration (Fig. S2), probably indicate species differences in *P. australis*. Posteriorly, an embayment between the otic capsules is separated by a posterior tectum from an endolymphatic depression into which the endolymphatic ducts open, as seen in stem chondrichthyans [15]. Neither structure had been identified for *P. gunni*, probably due to its crushed preservation.

Ventrally, the braincase is divided into pre- and postorbital regions by a transverse cranial fissure (Fig. 1a and d). The opening for the optic nerve passes through the fissure (Fig. 1e and f), this feature recalling the optic fissure in placoderms, but ventrally the fissure lies just behind the depression we interpret as the hypophysial fossa (Fig. S3), in that respect resembling the ventral fissure of osteichthyans. Anteriorly, a T-shaped prominent thickening forms the preorbital region, its prominent anterior process probably a species difference to the straight or embayed margin of *P. gunni* (Fig. S2). It is pierced by a pair of openings in the anterior cranial wall which might represent the passages for the olfactory nerve canals, if the ‘hemidomes’ of Sollas and Sollas [17] are interpreted as nasal capsules [3,6] (see Table S2).

Another striking feature is the long and broad undivided ventral braincase surface, representing basisphenoid and sub-otic regions posterior to the transverse cranial fissure (Fig. 1d). There is no sign of the ‘basicranial fenestra’ recently interpreted for *P. gunni* [7]. A medial trough (Fig. 1d and g) is interpreted to have overlain the dorsal surface of the notochord, and its sloping lateral walls show foramina and grooves interpreted as part of the cranial vascular system (Fig. S3a; see Supplementary Data). The otic capsules are large; the left side shows anterolateral and posterior depressions, with an intervening ridge that forms a lateral angle to the otic region (Fig. la and c–e). An elongate depression across the anterolateral face of the otic capsule is interpreted as a groove for the jugular vein (Fig. 1e), indicating that this vein did not traverse the braincase through a jugular canal. Articulation areas are developed on the anterolateral, lateral and posterolateral angles of the otic capsule (Fig. S3). In lateral view, the openings for the optic (II), and trigeminal (V) and facial (VII) nerves are preserved on the lateral wall of the neurocranium anterior to the otic capsules (Fig. 1e and f).

The 3D-preserved braincase of *P. australis* provides a unique opportunity to investigate the endocranial and inner ear cavities of this enigmatic fish (Fig. 2). The whole brain cavity is almost straight (Fig. 2a–b and e–f); brain divisions could not be distinguished, except for a small ventral swelling interpreted as the hypophysial fossa (Fig. 2e). In dorsal view, a pair of median depressions at the level of the connection of the anterior and posterior semicircular canals may represent the vestige of the endolymphatic sacs (Fig. 2a). The well-preserved labyrinth shows three semicircular canals (Fig. 2a, b, f and g), which confirms the gnathostome affinity of this animal. The semicircular canals in *P. australis* are broad, with no obvious expansions for ampullae. These were described for *P. gunni* [6], but it is possible that features of the membranous labyrinth are not preserved in the skeletal labyrinth [18].

**Figure 4.**
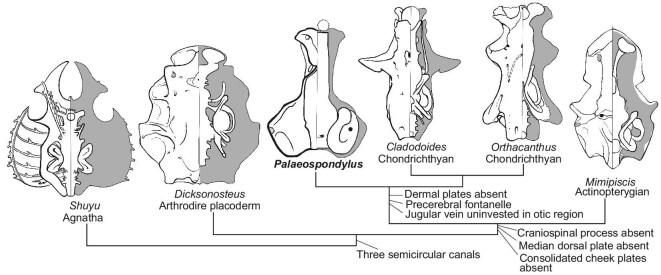
Simplified phylogeny showing the neurocranial evolution in early vertebrates. Left sides show ventral views of the braincase, and right sides show dorsal views of the endocast (images modified from references [14,16,19,20–22]). Significant apomorphies at each node are listed. *Palaeospondylus* is a sister group to the chondrichthyan clade. Full cladograms are provided in Figs S7 and S8.

The anterior semicircular canal is slightly longer than the posterior, and they are connected by a crus commune (Fig. 2e, g and h). Such a connection is present in lampreys, osteichthyans, chimaeroid chondrichthyans and some extinct agnathans and ‘placoderms’, but is absent in living and some extinct elasmobranchs [15,16]. It is striking that the external (horizontal) semicircular canal of *P. australis* is less canal-like, being doughnut-shaped (Fig. 2e and g). The space separating the external semicircular canal and sacculus is quite small, and forms a pillar-shaped hollow area (Fig. 2c and h). Such an arrangement recalls the early developmental pattern of the bony labyrinth in some modern gnathostomes, where the vestibule volume is much greater than the canals housed [18]. On the other hand, it could also represent a primitive crown gnathostome condition. In the bony labyrinth of other early gnathostomes (Fig. 4), the semicircular canals are much slenderer than in *P. australis* [16]. The stout rather than slender anterior and posterior semicircular canal spaces in the vestibule of *P. australis* recalls the condition in the galeaspid agnathan *Shuyu* [16,19]. As in osteostracan agnathans, there is no sinus superior in *P. australis.* The space for the sacculus of *P. australis* is small and flat, and does not form a distinct lower chamber in ventral view (Fig. 2h).

At least 11 different morphotypes of elements are identified and assigned to *P. australis*, based on their histological structure (Figs 1h and 3; Supplementary Data and Table S1). The most interesting of these element types (‘Type 1’) is the small square element pierced by a canal, which we identify as the proximal element of the hyoid arch (Figs 1h, 3j and s). The equivalent structure in *P. gunni* was referred to as a ‘gammation’ [17]; it is closely associated with the neurocranium in *P. gunni* (GA, Fig. S2d), and connected with an epal element of the hyoid arch, which articulated distally with the ceratohyal, and is thus part of the hyoid arch (Fig. 3j and s). This arrangement counters previous interpretations of the ‘gammation’, either as part of the mandibular arch [1,7], or as the postorbital process of the neurocranium [6]. We consider two possibilities—that the element is the hyomandibula, or that it is a lateral commissure that has not been incorporated into the neurocranium. The latter would be a unique adult vertebrate condition, and if correct lends further support to the interpretation of *Palaeospondylus* as paedomorphic.

Phylogenetic analyses (Fig. S7a) were run based on these two scenarios. Both these analyses show the same tree topology, and place *Palaeospondylus* as a sister group to the total group Chondrichthyes. This chondrichthyan affinity seems largely due to the absence of dermal bones and the presence of the precerebral fontanelle. However, some of the new characters demonstrated for *P. australis* are features more characteristic of osteichthyans, including the long dorsal fontanelle, separate paired occipital arches, mineralized hollow vertebral centra, and possibly a hyomandibula pierced by a canal. The most recent phylogenetic analyses for gnathostomes including *Palaeospondylus* [8,12], based on the anatomical interpretations that previously suggested a tetrapodomorph affinity [7], gave dramatically different results, with *Palaeospondylus* in a polytomy at the base of the Gnathostomata [12] or a tetrapodomorph [8]. As noted above, our new data show that many of the anatomical interpretations used in those analyses are erroneous. The phylogenetic analysis we ran using our character codings, but based on the same character set used in those studies [22], resulted in the same position for *Palaeospondylus* as in our other analyses (Fig. S7b). In order to test whether the position of *Palaeospondylus* could have been affected by the large number of characters that were inapplicable for that taxon, we ran a fourth analysis where we deleted characters in the first data matrix related to individual dermal elements (Fig. S8a and b). Yet again, the analysis showed *Palaeospondylus* as the sister group to the Chondrichthyes. In Fig. 4, we show a simplified cladogram comparing the neurocrania of *Palaeospondylus* with those of representatives of other early vertebrate clades.

## DISCUSSION

Many of the characters seen in *Palaeospondylus* appear to be retained larval characters—the total lack of a mineralized dermal skeleton, with the whole endoskeleton formed of a distinctive tissue that has been compared with incompletely developed endochondral bone [23], lack of distinct morphology in the endocranial cavity, a long dorsal fontanelle, an occipital arch separated from the otic region by an oticoccipital fissure, and very small size. These features all support an interpretation of *Palaeospondylus* as paedomorphic. As noted earlier, given the absence of any change in proportions or morphology with increase in size of the individual elements of *P. australis* and *P. gunni*, we consider the fossils to be adults, not larvae.

## CONCLUSION

Discovery of the mysterious animal *Palaeospondylus* in the Early Devonian of Australia indicates a likely worldwide distribution of the form, given that Scotland and eastern Australia were on opposite sides of the globe then as now. Our new evidence of the neurocranial features of *Palaeospondylus* adds critical, but conflicting, information regarding its affinities. Until new and superior evidence becomes available (for example, uncrushed 3D preservation as for *P. australis*, combined with complete articulated skeletons as for *P. gunni*), *Palaeospondylus* can be regarded as a paedomorphic stem gnathostome, perhaps sister group to the Chondrichthyes, showing a mosaic of features exhibited by osteostracans and some ‘placoderms’, as well as both chondrichthyans and osteichthyans.

## METHODS

### Specimen preparation

The vertebrate microremains were recovered by standard acetic or formic acid treatment of limestone samples collected in 2006 from the southern end of the Toomba Range in the Cravens Peak Beds, Georgina Basin, western Queensland [24]. For scanning electron microscopy, a JEOL 6300F machine at the Centre for Microscopy and Microanalysis, University of Queensland was used for platinum-coated specimens imaged at 10 kV, and a Hitachi TM-1000 Tabletop ESEM at the Queensland Museum was used for uncoated specimens. Ground thin sections were imaged using an Olympus BX-50 transmission microscope and DP-12 imaging system. Specimens are reposited in the Queensland Museum collection (QMF).

### X-ray computed tomography

QMF 52826 and QMF 53550 were scanned at the ‘National Laboratory for X-Ray Micro Computed Tomography’ (CT Lab) at the Australian National University (ANU). QMF 52826 was scanned twice (7 September 2017, 28 March 2019) using a HeliScan MicroCT system. The 2017 scan had a resolution of 10.96 μm, a 2 mm aluminium filter was used, the specimen distance was 58 mm from the source, and the detector position was 728 mm from the source, and probed separately with a polychromatic X-ray beam. Accelerating voltage of the electron beam generating Bremsstrahlung radiation was 120 kV with a current of 120 μA. Reconstruction was based on 3600 radiographic projections on a 2840 × 2840 Pixium Flat Panel camera. The 2019 scan used the newest optimized space-filling trajectory [25] and combined region-of-interest scan to yield sharp images [26] at a resolution of 5.42 μm (the highest possible resolution using any micro-CT scanner). A 0.75 mm aluminium filter was used, with a specimen distance of 17 mm from the source and a detector position 431 mm from the source; probed separately with a polychromatic X-ray beam. Accelerating voltage of the electron beam generating Bremsstrahlung radiation 80 kV with a current of 50A; reconstruction based on 3600 radiographic projections on a 3040 × 3040 Varian Flat Panel camera. QMF 53550 (11 elements, QMF 53550.1–QMF 53550.11) was scanned together (10 February 2014) in a 38 mm diameter pill bottle with a 2 mm aluminium filter (resolution of 10.32 μm, specimen distance of 75 mm and detector 1000 mm from the source; probed separately with a polychromatic X-ray beam). Accelerating voltage of the electron beam generating Bremsstrahlung radiation 120 kV with a current of 80 μA; reconstruction based on 2880 radiographic projections on a 2880 × 2880 Pixium Flat Panel camera. Scan data were analysed using Drishti v2.7 [27] and Mimics (http://biomedical.materialise.com/mimics; Materialise). After segmenting, surface meshes were rendered and imaged in Vayu (http://admorph.ivpp.ac.cn/download.html). Figures were compiled using Adobe Photoshop CS4.

### Phylogenetic analysis

Four data matrices were developed, with two using data matrices based on the analysis by Lu *et al*. [28] for the competing scenarios—‘gammation’ as the hyomandibula, or as the lateral commissure—comprising 367 and 368 characters respectively. The 96 taxa are the same as the 94 used [28] with the addition of *P. gunni* and *P. australis*. The third data matrix was run using the same base data set [22] as for the two most recent analyses of *P. gunni* [8,12], consisting of 99 taxa and 284 characters, with character codings revised in line with codings for our main analysis. In the fourth data matrix, in order to test whether the position of *Palaeospondylus* could have been affected by the large number of characters that were inapplicable for that taxon, we deleted characters in the first data matrix related to individual dermal elements—i.e. chars. 5–69, 76–91, 93, 97, 103, 107–111, 113, 175–184, 186–201, 204–221, 225–227, 230–238, 241–253, 257, 260–263, 265, 269–271, 273–276, 280, 281, 283–300, 302–306, 310–330, 354, 356–360—and added new characters (#142–145 in the fourth matrix)—dermal scales: 0, present; 1, absent; dermal plates: 0, present; 1, absent; fin spines: 0, present; 1, absent; teeth: 0, present; 1, absent. This data matrix thus used 145 characters.

All data entry and formatting was performed in Mesquite (v.2.5); all characters were unordered and unweighted. Data were processed using TNT (v.1.5) software [29] for maximum-parsimony analysis using a ‘New technology search’, with Galeaspida and Osteostraci as the outgroups. Default settings were used for most parameters, but the value for ‘random additional sequence’ was changed from 1 to 1000, and the Ratchet and Drift were also used during tree search. Bremer support values were generated in TNT using the script ‘Bremer.run’.

## Supplementary Material

nwae444_Supplemental_File

## Data Availability

All data analysed in this paper, including the phylogenetic data sets, are available as part of the article, Figs S1–S8, Tables S1 and S2, and the Supplementary Data. Supplementary Data are available at Figshare (https://figshare.com/s/5315534e4deb2b0a5f9e). The Life Science Identifier (LSID) for the new species has been deposited at ZooBank:LSIDurn:lsid:zoobank.org:act:B0A666F5-ED95-4782-94AC-F24851973AEC.
