# Correction to: A 3D Braincase of the Early Jawed Vertebrate *Palaeospondylus* from Australia

**DOI:** 10.1093/nsr/nwaf197

**Published:** 2025-07-10

**Authors:** Carole J Burrow, Gavin Young, Jing Lu

**Affiliations:** Geosciences, Queensland Museum, Hendra 4011, Australia; Department of Materials Physics, Australian National University, Canberra 2600, Australia; Australian Museum Research Institute, Australian Museum, Sydney 2010, Australia; Key Laboratory of Vertebrate Evolution and Human Origins of Chinese Academy of Sciences, Institute of Vertebrate Paleontology and Paleoanthropology, Beijing 100044, China; University of Chinese Academy of Sciences, Beijing 100049, China

In the article by Burrow *et al.*, ‘A 3D Braincase of the Early Jawed Vertebrate *Palaeospondylus* from Australia,’ *National Science Review* 2025; **12**: nwae444. DOI: 10.1093/nsr/nwae444, an error was identified in Fig. 3.

**Error**: The labels in Fig. 3 were inconsistent with the figure legend.

**Correction**: The labels have been corrected as follows:

m → q
n → m
o → n
p → o
q → p

The corrected Fig. 3 is presented below.

We apologize for any inconvenience caused. This correction does not affect the conclusions of the article.

**Figure 3.**
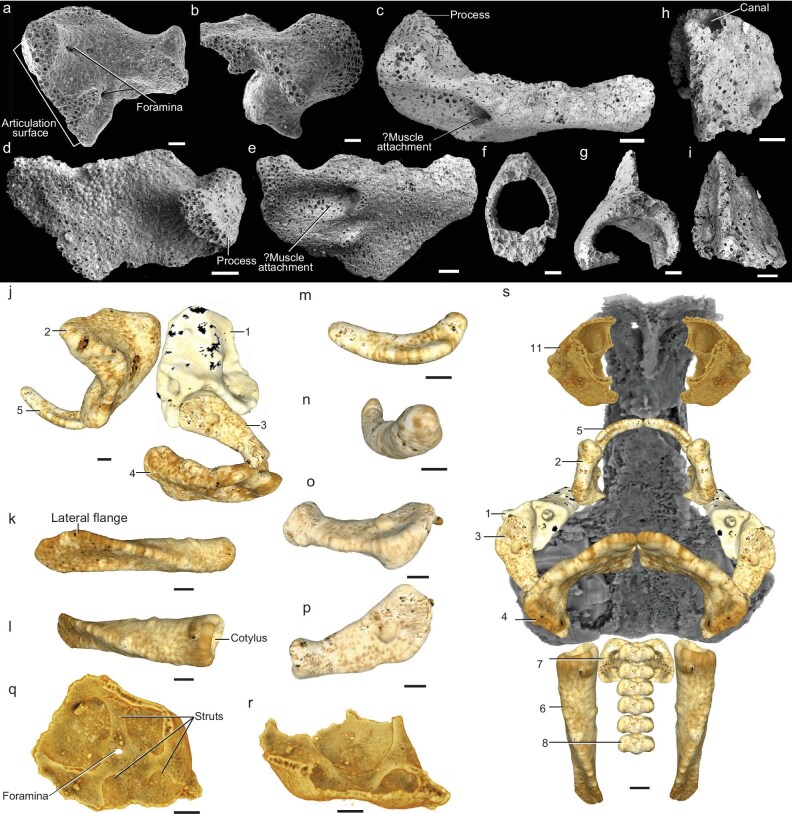
SEM and 3D scan images of *P. australis* sp. nov. isolated elements. (a, b) QMF53547, Type 2 element, possible left palatoquadrate in dorsaomedial and dorsolateral views. (c-e) QMF52830, Type 4 element, ceratohyal, in dorsal, ventral and anterior views. (f) QMF52832, Type 8 element, vertebral centrum. (g) QMF52843, Type 9 element, neural or haemal arch with spine. (h, i) QMF52831, Type 7 element, occipital arch, apex and lateral views. (j) Possible arrangement of the left side mandibular and hyoid arch elements QMF53550.1, 3, 4, 5, 9 in lateral view. (k, l) QMF53550.11, Type 6 element, right postoccipital lamella, ventrolateral and dorsolateral views. (m, n) QMF53550.3, Type 5 element, possible Meckel's cartilage, ?anterodorsal and anterolateral views. (o, p) QMF 53550.4, Type 3 element, interhyal (or hyomandibula). (q, r) QMF52828, Type 11 element, ‘hemidome’ sensu Sollas and Sollas [17] (possible nasal capsule), internal view and lateral/anterior view. (s) Reconstruction of the neurocranium (grey) and associated elements as in (j) plus vertebral centra, occipital arches and postoccipital lamellae QMF53550.7, 8, 11, ventral view. Scale bars, 0.1 mm; (a–i) scanning electron micrographs; (j–s) 3D microCT scan images; (j–p) Meshlab images; (q, r, s) Drishti images. Type 1–11 elements, see Supplementary Data and Table S1.

