# Supplementary material for: A 3D braincase of the early jawed vertebrate Palaeospondylus from Australia
Source: Natl Sci Rev. 2024 Dec 3;12(5):nwae444. doi: 10.1093/nsr/nwae444 (PMC11970237; doi:10.1093/nsr/nwae444)
Supplement: nwae444_Supplemental_File [file nwae444_supplemental_file.docx]

Supplementary Information for

**A 3D braincase of the early jawed vertebrate *Palaeospondylus* from Australia**

Carole J. Burrow^1^, Gavin C. Young^2,3^, and Jing Lu^4,5,6*^

^1^Geosciences, Queensland Museum, Hendra 4011, Australia. ^2^Department of Materials Physics, Australian National University, Canberra 0200, Australia. ^3^Australian Museum Research Institute, Sydney 2010, Australia. ^4^Key Laboratory of Vertebrate Evolution and Human Origins of Chinese Academy of Sciences, Institute of Vertebrate Paleontology and Paleoanthropology, Beijing 100044, China. ^5^University of Chinese Academy of Sciences, Beijing 100049, China.

*Corresponding author: [lujing@ivpp.ac.cn](mailto:lujing@ivpp.ac.cn)

Contents

1. Geological setting
2. Histology of *Palaeospondylus australis* sp. nov.
3. Restoration and description of the neurocranium for *Palaeospondylus australis* sp. nov.
4. Description of other material referred to *Palaeospondylus australis* sp. nov.
5. Relationships of *Palaeospondylus*
6. SI Figures
7. Phylogenetic Data
8. Description of supplementary data and video
9. References

**I. Geological setting**

The type locality is a small limestone outcrop designated GY6-11 (Burrow & Young 2005), at the southern end of the Toomba Range, on the western flank of Toko Syncline in the Georgina Basin, western Queensland, Australia. This remote locality, on the northern edge of the Simpson Desert, has yielded dissociated elements from a rich vertebrate fauna, the first discovery being Devonian acanthodian and thelodont scales (Turner *et al*. 1981). Subsequently described are thelodont agnathans (Turner 1995, 1997), asterolepid antiarchs (Young 1984), the ‘notidanid’ shark *Maiseyodus* Long *et al.,* 2021 (*Mcmurdodus* of Turner & Young 1987, Burrow *et al.* 2008), various acanthodians (Burrow & Young 2005), and osteolepid, holoptychiid, dipnoan, and onychodontid osteichthyans (Young & Schultze 2005, Burrow & Turner 2012). Several other groups await description. The *Maiseyodus* shark teeth are the oldest of neoselachian design from the fossil record (Capetta *et al.* 1993), and the antiarch is the oldest known representative of the order Asterolepiformes (Young & Lu 2020).

The age and stratigraphy of the outcrop have been discussed by Turner *et al*. (1981), Young (1996), Young & Turner (2000), Young & Goujet (2003), Burrow & Young (2005), Young & Schultze (2005), and Young *et al*. (2010). Calcareous beds in the area represent the base of the Devonian Cravens Peak Beds. However, possible faulting at the limestone outcrop producing *Palaeospondylus* *australis* sp. nov. makes precise field relationships uncertain. The overlying clastic sequence contains a diverse vertebrate fauna of the *Wuttagoonaspis* assemblage (Young 1991, Young & Goujet 2003), which indicates a total age range of no older than Pragian, and no younger than earliest Eifelian (Young & Goujet 2003). Associated invertebrates from the basal calcareous beds include ostracods very close or identical to *Healdianella subdistincta*, described by Wang (1983) from the late Emsian Sipai Formation of Guangxi Zhuang Autonomous Region in southern China. Thus, all the palaeontological evidence indicates a likely Emsian age for *Palaeospondylus* *australis* sp. nov.

Young & Lu (2020) discussed the biogeographic and palaeogeographic setting for the Cravens Peak Beds fauna. The vertebrate component shows mainly Gondwanan affinity, rather than the South Chinese affinity indicated by the marine invertebrates (ostracods). The widely distributed thelodont *Turinia* (Gondwana, and also the South China block, and west Yunnan, part of the Shan-Thai terrane), evidently could disperse through marine environments. The mcmurdontid sharks *Mcmurdodus* and *Maiseyodus* seem clearly Gondwanan forms (first described from Antarctica). In the overlying *Wuttagoonaspis* assemblage the armoured agnathans *Pituriaspis* and *Neeyambaspis* show affinity to the huananaspid galeaspids from South China (Young & Lu 2020, fig. 2). The only other suggestion of a possible South Chinese element concerns unusual ‘osteolepid’ scales with posterior serrations from the basal limestone (Young & Schultze 2005, Schultze 2016). Friedman & Brazeau (2010) noted a resemblance to scales of the early osteichthyan *Guiyu* Zhu *et al*. (2009) from the Silurian (Ludlow) Kuanti Formation of Yunnan Province.

A marginal marine palaeoenvironment is proposed for the new *Palaeospondylus* locality (the limestone contains stromatolites; R.V. Burne *pers. comm*.). Stronger marine incursions (sea level rise) would explain the marine invertebrates, perhaps associated with vertebrate faunal exchange with South China related to the E’Em bioevent (Pragian-Emsian boundary) recognized by Zhu (2000). A similar palaeoecological interpretation for the type locality of *Palaeospondylus gunni* involves water depth fluctuations in a large lake system, perhaps related to Milankovitch cycles. The fish beds, including the Achanarras horizon (*Palaeospondylus*), have been associated with maximum lake expansion that involved occasional overflow into the sea (Stephenson *et al*. 2006). These rare marine connections (Marshall *et al*. 1996, 2007), explain the same fish taxa occurring in shallow marine environments of the Baltic region (Mark-Kurik & Poldvere 2012). Mass death events (algal blooms?) produced concentrations of vast numbers of *Palaeospondylus* specimens in a single set of laminites with less than one metre total thickness, this taxon apparently a chance introduction from an outside area related to one of the marine incursions (Trewin 1986). Hirasawa *et al*. (2022) also suggested turbidity currents as a possible factor.

**II. Histology of *Palaeospondylus australis* sp. nov.**

Isolated elements from the GY6-11 locality were interpreted on histology to belong to *Palaeospondylus* before the holotype braincase was discovered (Burrow *et al.* 2014). The internal structure of all elements shows a foam-like architecture (Fig. 1b, Supplementary Fig. S1a-d). The hard tissue forming the scaffold shows no evidence of bone cell lacunae, fibrous strands, or any other indication of collagen. The subspherical to polyhedral spaces between the thin trabeculae of the hard tissue are 30-40 μm in diameter, and the struts are less than 5 μm wide. As far as we can determine, the neurocranium and isolated elements are wholly composed of this tissue. The trabeculae in the tissue mostly lack a regular pattern except in the outermost subsurface layer where the ‘cells’ are aligned with the surface (Supplementary Fig. S1c). The outer surface of the elements appears to be formed of the same tissue as the trabeculae, but may be slightly thicker. This tissue is presumed to be a mineralized extracellular matrix produced by the cells that originally occupied the spaces. The same type of structure is otherwise only known to occur in *Palaeospondylus gunni* (Supplementary Fig. S1e, f). Although not as well preserved, the tissue in the latter shows the same architecture and dimensions as in *P. australis*. In *P. gunni*, the intertrabecular spaces were interpreted as originally occupied by large hypertrophic cartilage cells, and the trabeculae are composed of calcium and phosphorus, consistent with biological mineralisation as hydroxyapatite. Thin sections of *P. gunni* show an outer layer that is largely unmineralized, with fewer lacunae, described as the sub-perichondrium (Johanson *et al*. 2010, fig. 4). This layer and the lacunal ‘spaces’ in *P. gunni* are composed mainly of carbon, whereas in *P. australis* there is no evidence of a differentiated outer layer. Possibly this is a result of the different preservation (especially diagenetic changes in *P. gunni*) and preparation of specimens (acid etching of the *P. australis* elements). Unlike Hirasawa *et al*. (2022), who identified a small area of tissue in *P. gunni* as perichondral bone, we have not seen evidence for perichondral bone in *P. australis*. Although the Hirasawa *et. al*. (2022) study was based on synchrotron scanning, we note that perichondral bone is normally readily recognized by other means of investigation.

**III. Restoration and description of the neurocranium for *Palaeospondylus australis* sp. nov.**

Articulated specimens normally enhance our understanding of fossils, but for *Palaeospondylus* *gunni* this has hampered interpretation of the neurocranium. All Scottish specimens are compressed in black shale, making it uncertain what structures were actually part of the neurocranium. The *Palaeospondylus* *australis* holotype (QMF 52826) is an isolated neurocranium preserved uncrushed in limestone, so that issue does not arise.

The new neurocranium overall shows a rather granular texture, resulting from the unique histology of *Palaeospondylus* described above. Some sedimentary abrasion could have occurred in gentle currents that disassociated other skeletal elements before the specimen was embedded in the sediment. Slight projections that are less pronounced on one side of the neurocranium than the other are assumed to result from minor abrasion. Other disarticulated vertebrate remains from this deposit are generally well preserved, with little evidence of abrasion.

Preserved neurocranial length for QMF 52826 is ~3.6 mm. Total length for numerous studied specimens of *P. gunni* (Sollas & Sollas 1904, p. 270; Moy-Thomas 1940, p. 394; Trewin 1986, pp. 36–37) varies from 13-14 mm to over 50 mm. The overall morphology of *P. gunni* is not size-dependent, with recognised features showing the same proportions and architecture in all published and observed specimens. The smallest neurocranium is about 2.2 mm long, with average length 4-5 mm (dimensions excluding anterior rostralia and posterior occipital elements). The largest figured specimen (total length ~62 mm; Newman & den Blaauwen 2008) has a neurocranium just over 8 mm long, so *P. gunni* attained twice the size of the *P. australis* holotype, which is of comparable size to average neurocranial length for *P. gunni*.

QMF 52826 demonstrates that separate elements of the visceral skeleton have been erroneously interpreted as neurocranial structures in almost all previous studies of *P.* *gunni*, including detailed new descriptions based on the latest synchrotron and CT scanning techniques (Johanson *et al*. 2017, Hirasawa *et al*. 2022). Hirasawa *et al*. (2022) attempted synchrotron micro-computed tomography to identify cell lacunae indicating boundaries between cranial and visceral arch structures, but our new evidence shows this was not effective.

As noted, the most reliable previous reconstruction that separates visceral arch from neurocranial structures is the seminal work of Moy-Thomas (1940), who used traditional methods to study nearly 500 specimens of *Palaeospondylus gunni*, providing a neurocranial restoration that compares best with the new evidence of the *P. australis* holotype. Our restorations for *P. australis* are presented in dorsal, ventral, and left lateral views (Supplementary Fig. S2c, S3a-b), and compared with dorsal and ventral views of *P. gunni* by Moy-Thomas (Supplementary Fig. S2a-b). Displayed in the new specimen are various foramina (labelled fo1-12), notches (n1-4), and grooves, most of which have never been observed before in *Palaeospondylus*. We use non-committal terminology for these, and their comparative morphology is discussed in the descriptions below.

**Dorsal aspect**

Basic similarities in neurocranial restorations for the two *Palaeospondylus* species (Supplementary Fig. S2a-b) include a much-expanded posterior otic region that is roofed over (tectum synotum), and a narrow unroofed pre-otic region, the latter evidently more elongate in *P. australis*. Moy-Thomas (1940) considered the ‘ampyx’ and ‘dorsal rostralia’ at the anterior end of the neurocranium in *P.* *gunni* to be separate structures (AM, DR, Supplementary Fig. S2d), and this is confirmed by their absence in the *P. australis* holotype. Moy-Thomas interpreted these as parts of anterior nasal capsules (also Hirasawa *et al*. 2022); other authors (e.g. Sollas & Sollas 1904, Johanson *et al*. 2017) interpreted the more posteriorly positioned ‘hemidomes’ as nasal capsules. In either case they were not fused into the neurocranium. De Beer (1937, p. 394) considered that fusion of nasal capsules to the neurocranium in gnathostomes generally was a secondary condition, because in *Petromyzon* they are attached only by connective tissue. For Palaeozoic fossils that condition could not necessarily be distinguished due to lack of mineralization of the nasal capsules. Unmineralized, or incompletely mineralized nasal capsules (as in *Palaeospondylus*) occur in various crown gnathostomes, including *Acanthodes*, and various chondrichthyans (Brazeau & Friedman 2014).

Other general resemblances to the Moy-Thomas (1940) neurocranial restoration are the posterior dorsal embayment, the absence of an occipital division to the neurocranium, and the elongate dorsal fontanelle (d.font) comprising at least half total braincase length (Supplementary Fig. S2a-b). The dorsal fontanelle is flared out anteriorly in *P. australis* (prc.font, Supplementary Fig. S2b), equivalent to the ‘precerebral fontanelle’ of Johanson *et al*. (2017, fig. S1A) for *P. gunni*. Moy-Thomas (1940) restored the T-shaped anterior ‘tauidion’ (as named by Sollas & Sollas 1904) as anteriorly concave (Supplementary Fig. S2c), but our material (Supplementary Fig. S6e-j) shows an anteriorly projecting median ventral process (mv.pr, Supplementary Fig. S3), assumed to be a specific difference in *P. australis*. A constriction midway along the dorsal fontanelle in both *Palaeospondylus* species (Supplementary Fig. S2a-b) is reminiscent of the tectum orbitale (te.orb) in *Squalus* embryos (e.g. Holmgren 1940, fig. 69). The dorsal fontanelle of *P.* *gunni* was restored as broad and slightly rounded posteriorly, compared with the pointed posterior shape of the new species; this could be another specific difference. Anteriorly, the cylindrical floor of the cranial cavity of *P. australis* is visible within the dorsal fontanelle, pierced by conspicuous foramina that exit ventrally through the ‘tauidion’ region (fo7, Supplementary Figs. 2b, 3).

The roof of the otic region is restored as a flat featureless surface for *P.* *gunni*, with a simple shallow posterior embayment (Supplementary Fig. S2a). This lack of detail is probably due to the crushed preservation. *P. australis* shows a deep v-shaped posterior median embayment, and a ventral median process (mp.pr). A central depressed area between otic capsules is flanked by thick posterolateral and anterolateral ridges (r.asc, r.psc, Supplementary Fig. S2b), reflecting the position of underlying semicircular canals. The central depression extends anteriorly as diverging deep grooves (gr.al, Supplementary Fig. S4a). Behind is an irregular shallow depression (dep.end), delimited posteriorly by ridges arched over two conspicuous foramina opening upwards and forwards into the depression (te.p, d.end, Supplementary Fig. S4a). Restored in dorsal view, the ridge is obliquely oriented over the asymmetrically placed openings (Supplementary Fig. S2b). This irregular depression has a comparable position to the endolymphatic fossa of living chondrichthyans, for example *Squalus* (Maisey 2001a, fig. 7) or *Chlamydoselachus* (Allis 1923, fig. 9). In *Chlamydoselachus* the fossa has a floor, also pierced by posterior openings for the endolymphatic ducts beneath a posterior ridge (but in this case these structures are symmetrical). In Palaeozoic chondrichthyans, the endolymphatic fossa is open through to the cranial cavity, and orientation or position of any openings for paired endolymphatic ducts is uncertain. The fossa may be bounded posteriorly by a transverse ‘posterior tectum’ (*Tamiobatis*; Schaeffer 1981), or it opens posteriorly into the occipital fissure (*Cladodoides*, Maisey 2005; *Doliodus*, Maisey *et al.* 2009). The Middle Devonian *Pucapampella* is restored with a transverse ‘synotic tectum’ anterior to a fossa interpreted to contain the endolymphatic duct openings (Maisey 2001b, fig. 16.1a). In the latter three taxa the endolymphatic fossa straddles or represents the dorsal part of the oticoccipital fissure (the posterior dorsal fontanelle of various osteichthyans may be homologous; Gardiner 1984, p. 202; Brazeau & Friedman 2014, fig. 8). Based on these comparisons, *P. australis* is interpreted to differ from Palaeozoic chondrichthyans in having a floored endolymphatic depression, with the equivalent of a posterior tectum separating it from the deep v-shaped dorsal embayment of the neurocranium. The latter would be equivalent to the dorsal part of the oticoccipital fissure. Neither Johanson *et al*. (2017) nor Hirasawa *et al*. (2022) show any of these structures.

The posterior embayment is more pronounced in *P. australis* than in any neurocranial restoration for *P. gunni*. It comprises a deep v-shaped indentation dorsally, above a slightly concave upper border to the lozenge-shaped posterior opening into the cranial cavity (cr.cav, Supplementary Fig. S4b). Beneath this, a median posterior process is deflected slightly upwards in posterior view (mp.pr, Supplementary Figs. S2b, S3a). A dorsal crest (cr) forms a double projection posteriorly (pd.pr).

The posterior embayment is comparable with the posterior dorsal fontanelle in actinopterygians, confluent with the oticoccipital fissure, and identified by Brazeau & Friedman (2014, fig. 8B) as an osteichthyan character. Johanson *et al*. (2017) noted ‘small comma-shaped structures’ identified as occipitals, and suggested these were connected to the otic capsules across a presumed oticoccipital fissure. Hirasawa *et al*. (2022, fig. 1b) distinguished three ‘anterior occipital arches’ referred to as the ‘basioccipital part of the neurocranium’. However, *P. australis* confirms earlier opinions (e.g. Stensiö 1927, Moy-Thomas 1940) that the occipital division was not incorporated into the neurocranium of *Palaeospondylus*.

**Ventral aspect**

The otic capsules are abraded ventrally due to exposure on the limestone surface (Fig. 1a, d), but a posterior view shows they were gently convex. The more complete left capsule exhibits anterolateral and posterolateral depressions (dep.al, dep.pl, Supplementary Fig. S3a) separated by a low ridge (r.lat), possibly traversed by at least one canal passing posterolaterally between irregular foramina (fo11,12, suggested by a groove within the floor of the otic capsule exposed on the opposite side; Supplementary Fig. S5a-b). A somewhat similar posterolateral depression on the otic capsule is interpreted as a jugular groove for the enigmatic *Ramirosuarezia* (Pradel *et al.* 2009, fig. 2B).

The lateral ridge in *P. australis* curves up to a slightly bilobed lateral angle (la). A more prominent ‘lateral otic process’ for *P. gunni* (Johanson *et al*. 2017, Hirasawa *et al*. 2022) must have been accentuated by compression (corrected for in the Moy-Thomas restoration). The lateral otic process of Palaeozoic chondrichthyans is defined as partly enclosing the posterior semicircular canal (Maisey 2005, p. 88), whereas the structure so named in *P. gunni* by Johanson *et al*. (2017) projects at the level of the middle of the horizontal semicircular canal.

Hirasawa *et al*. (2022, fig. 3a) also identified a ‘pro-otic process’, equivalent to the more-rounded anterolateral angle of our reconstruction (ala, Supplementary Fig. S3a). As was evidently assumed by Moy-Thomas (1940), who did not show it, this was also accentuated by compression in *P. gunni*. In the same position Johanson *et al*. (2017, fig. 1d) show a more prominent ‘postorbital process’. This structure is the ‘gammation’ of Sollas & Sollas (1904). The new evidence of *P. australis* confirms the interpretation of Moy-Thomas (1940) that this was a separate element from the neurocranium (GA, Supplementary Fig. S2d).

A less distinct ridge in *P. australis* (r.pvl) passed to a posteroventral angle (pva, Supplementary Fig. S3b), the latter also accentuated by compression in *P. gunni*. There is no indication of the bulbous ventral projection (‘articular facet for the hyomandibula’) identified by Hirasawa *et al*. (2022, suppl. figs. 4c, 5b). Moy-Thomas (1940) interpreted this ‘projection’ as a separate branchial arch (BA, Supplementary Fig. S2d), as did Johanson *et al*. (2017) and all previous workers. Many illustrated specimens clearly indicate that this was a separate element, being asymmetrically displaced between left and right sides (e.g. Johanson *et al*. 2017, fig. S1B). This also invalidates the ‘lateral commissure’ interpretation of Hirasawa *et al*. (2022, fig. 3a-b), identified only because it carried their hypothesized hyomandibular articulation. The *P. australis* holotype confirms that structures in this position were not part of the neurocranium.

In general, the shape of the otic capsule in *P. australis* confirms the accuracy of Moy-Thomas’s (1940) restoration for *P. gunni*, and his interpretation that neither the ‘gammation’ (‘postorbital process’ of Johanson *et al.* 2017), nor his posterior branchial arch (‘hyomandibular articulation’ of Hirasawa *et al*. 2022), were part of the neurocranium.

In contrast, other ‘branchial arches’ putatively crushed against the *P. gunni* braincase (Sollas & Sollas 1904) were correctly interpreted by Moy-Thomas (also Bulman 1931) as neurocranial. These formed thickened anterior edges to the otic capsules (r.prot, Supplementary Fig. S2c), and are clearly shown in the CT-scanned specimen of Hirasawa *et al*. (2022). A possible comparison is with the ‘subcranial ridges’ under the otic capsules of *Doliodus* (Maisey *et al*. 2009) and *Janusiscus* (Giles *et al*. 2015). Moy-Thomas restored a rounded ‘articular surface’ on this ‘preotic’ ridge (Supplementary Fig. S2c). In *P. australis* an indistinct depression in an equivalent position is only seen on the right side (?art, Fig. 1d, f). This area seems less complete on the left side, and the restoration assumes it was lost by abrasion, to indicate paired articular facets in a position corresponding to those in *P. gunni* (art.av, Supplementary Fig. S3a). More anteriorly, the left side of the *P. australis* holotype seems better preserved, indicating the preotic ridge turning forward to project ventrally beneath the orbit; this roughly longitudinal part is termed the ‘suborbital ridge’ (r.so; Supplementary Fig. S3a).

Between the otic capsules a median longitudinal depression extends forward for some two thirds of total braincase length (Supplementary Fig. S3a), a feature never identified in the crushed *P. gunni* specimens. Foramina and paired longitudinal grooves on the lateral sloping sides of the depression are interpreted as vascular structures (described below), and the smooth central part is interpreted as the notochordal groove (gr.not). Anteriorly, the depression decreases in width between the prominent suborbital ridges (r.so), and narrows to a point behind the distinct cleft for the transverse fissure (trans.f), with paired foramina just behind (fo6, Supplementary Fig. S3a). In front, a prominent posteroventral projection (fo.hyp) contains the interpreted hypophysial fossa identified in the endocast of the cranial cavity (Fig. 2e). More posteriorly, the floor of the endocast is featureless, so identifying this posteroventral projection as the hypophysial fossa seems the only reasonable interpretation. It is placed farther forward than the ‘fossa hypophyseos’ identified by earlier workers for *P. gunni*, ‘at the transverse plane through the anterior ends of the otic capsules’ (Stensiö 1927, p. 376), but Moy-Thomas (1940) considered that supposed change in the level of the neurocranial floor largely due to compaction under pressure. There is no indication of a bucco-hypophysial foramen in *P. australis*.

In stem chondrichthyans (e.g. *Cladodoides*, Maisey 2005; pucapampellids, Maisey *et al.* 2019) the notochordal canal was confined to the parachordal plate behind the level of the oticoccipital fissure (not known for *P. australis*). The position or existence of a notochord al canal was neither identified nor discussed in early descriptions of *P. gunni* (e.g. Stensiö 1927; Moy-Thomas 1940). Hirasawa *et al*. (2022) proposed a completely different restoration for *P. gunni*, with the notochord extending forward past the otic capsules to a ‘notochordal pit’, in front of an ‘arcual plate’ that covered a ‘basicranial fenestra’. Our new evidence shows no indication of an ‘arcual plate’, which we consider to be a basibranchial (BB, Supplementary Fig. S2d), as interpreted by Moy-Thomas (1940). Similarly, we have no evidence of a ‘basicranial fenestra’. The detailed analysis of serial sections by Sollas & Sollas (1904) noted ‘no signs of a basi-cranial fontanelle’, and this structure was never identified by other researchers. The interpreted ‘basicranial fenestra’ of Hirasawa *et al*. (2022, fig. 3b) is an irregular opening much smaller than the large median fenestra of stem tetrapodomorphs like *Tungsenia*, *Gogonasus* or *Eusthenopteron* (Jarvik 1996; Long *et al.* 2006; Lu *et al.* 2019). We suggest that the putative ‘basicranial fenestra’ could be a remnant of the longitudinal depression of *P. australis*, the rest being obliterated by crushing.

For *P. australis*, the extent that the notochord continued in front of the oticoccipital fissure is unknown, and it may have been limited only to the posterior part. As documented for modern neurocrania (de Beer 1937, pp. 380-381), there is much variation both in chondrification of the paired parachordals, and their fusion into a completed basal plate around the notochord. The notochord may persist as a well-developed adult structure (e.g. cyclostomes, chondrichthyans, dipnoans, *Latimeria*), but in other groups it is much reduced, and restricted to the hinder region of the basal plate. The parachordals may fuse above and below the notochord to form a notochordal canal, or fuse just below it (with the notochord or its remnants situated dorsal to the basal plate), or fuse above it (when they are situated ventral to the basal plate). By comparison with an Early Devonian chondrichthyan like *Gydoselache*, which has a notochordal canal (Maisey & Anderson 2001, fig. 1), our restoration for *P. australis* (Supplementary Fig. S3a) would indicate fusion of the paired parachordals above the notochord but not beneath it, leaving only the notochordal groove (gr.not).

Further anteriorly, the interpreted ‘vestibular fontanelles’ of *P. gunni* (Hirasawa *et al*. 2022, fig. 3b) could be another remnant of the longitudinal depression of *P. australis*, the rest being obliterated by crushing. These openings are minute by comparison with the very large paired fontanelles of *Eusthenopteron*, *Gogonasus*, and other sarcopterygians. The detailed morphology of this region for *Palaeospondylus* is revealed for the first time by the uncrushed *P. australis* holotype. The only openings here are two pairs of much smaller foramina (fo1, 2, Supplementary Fig. S3a), associated with grooves evidently representing part of the cranial vascular system (see below). In summary, the *P. australis* holotype provides no evidence for any of the interpreted ‘sarcopterygian/stem tetrapod’ neurocranial structures proposed by Hirasawa *et al*. (2022).

The anterior part of the neurocranial floor in *P. gunni* has been restored completely differently (Supplementary Fig. S2c), but in all specimens this area is obscured by visceral arch elements crushed up onto the neurocranial floor. Hirasawa *et al*. (2022) also identified a median bucco-hypophysial foramen, flanked by curved basipterygoid processes, with paired foramina for the internal carotids in front. The *P. australis* holotype confirms earlier interpretations that these curved structures (AT, Supplementary Fig. S2d) are separate visceral arch elements (‘anterior trapezial bars’ of Moy-Thomas 1940). Based on evidence of associated vascular grooves (see below) we identify another lateral projection as a possible palatobasal connection (bpt, Supplementary Fig. S3a), and a different course for the internal carotid artery. The ‘internal carotid’ foramina of Hirasawa *et al*. (2022) could be lateral openings for the optic nerve that have been crushed down into a ventral position; these are just visible ventrally in *P. australis* (II, Supplementary Fig. S3a).

Anteriorly, the hypophysial fossa of *P. australis* narrows to a median ridge continuous with the posterior part (mv.sept) of the T-shaped ‘tauidion’ (TA). This was previously interpreted either as an ‘anterior palatal ossification’ of the neurocranium (Moy-Thomas 1940, p. 410), or as a separate element (e.g. Forey & Gardiner 1981, Thomson *et al.* 2003, Johanson *et al*. 2017). *P.* *australis* confirms Moy-Thomas’s interpretation that the ‘tauidion’ was part of the neurocranium. Paired lateral grooves running from conspicuous foramina through the ‘tauidion’ into the cranial cavity (l.gr, fo7, Supplementary Fig. S3) meet posteriorly at a slight median notch (n3). The anterior margin of the ‘tauidion’ is incomplete in the *P.* *australis* holotype, but a strong median ventral process (mv.pr) is well preserved in two fragmentary specimens (QMF 52827.1, 2; Supplementary Fig. S6e-j).

**Lateral aspect**

The first uncrushed lateral views of the neurocranium for *Palaeospondylus* (Fig. 1e, f) show clear evidence of two major openings for cranial nerves (II, V+VII, Supplementary Fig. S3b). The neurocranium is most shallow at the sharp upward inflection into the transverse fissure (trans.f), which is delimited in front by the posteroventral projection containing the hypophysial fossa (fo.hyp). The upper margin of the dorsal fontanelle slopes downwards to an anterior notch (n4). The median ventral septum and process of the ‘tauidion’ (mv.sept, mv.pr) project strongly downwards to the anterior margin. A subocular shelf (sos) projects laterally in front of the otic capsules to confine the orbital cavity (orb, Supplementary Figs. S2b, S6b). The optic nerve must have turned posteriorly from its foramen to reach the eyeball, as for example in *Chlamydoselachus* (Allis 1923, fig. 52). Johanson *et al*. (2017) interpreted the orbit to be much more restricted, but the structure they considered the ‘vertical anterior wall of the orbit’ is shown by the *P. australis* holotype to be an element separate from the neurocranium.

Both sides of the *P. australis* holotype show irregular horizontal ridges behind the optic foramen carrying a small concavity opening anteriorly (eys, Supplementary Figs. S3b, S5b, S6d), provisionally interpreted as the attachment for an optic pedicle (eye-stalk). *Cladodoides* shows a similar morphology behind the optic nerve foramen. The more complete left orbit of *P. australis* shows a bilobed deep depression above the subocular shelf, suggesting a myodome for extra-ocular muscles. It contains two significant openings (fo8, fo9), and is delimited above from the trigemino-facialis opening by a horizontal ridge (r.h1). In some other groups the ophthalmic artery reached the orbit through a foramen in a ventral myodome (e.g., Young 1986, fig. 7, Clement *et al.* 2018, fig. 4C), a possible interpretation for one of these openings.

This general configuration bears little resemblance to previous descriptions of the ‘floor of the cranium’ for *P. gunni*. In a comparison with elasmobranchs, Sollas & Sollas (1904, p. 276) argued that a step-like descent or ‘saddle ledge’ in the neurocranial floor beneath their presumed optic foramen indicated the hypophysial fossa, an interpretation supported by Stensiö (1927). However, Moy-Thomas (1940 p. 396) considered the ‘step-like descent’ an artefact of preservation, and restored the neurocranium floor as flat and featureless. The XCT scanned specimen of Hirasawa *et al*. (2022, fig. 3b-c) is clearly much compressed, as depicted in their oblique lateral view, and their ‘bucco-hypophysial foramen’ is much farther back than the hypophysial fossa of *P. australis*. This foramen, placed behind the level of our transverse fissure (not identified by Hirasawa *et al*. due to crushing), could be a misinterpreted part of the vascular system.

**Neurocranial subdivisions (cranial fissures)**

Moy-Thomas (1940, p. 395) concluded there was no occipital division incorporated in the neurocranium. All subsequent workers agreed, except for Hirasawa *et al*. (2022), who proposed two occipital arches fused into the otoccipital portion of the neurocranium. Contrary to this, the evidence of *P. australis* confirms the interpretation of earlier workers, that *Palaeospondylus* had a complete **oticoccipital fissure**. The evidence of *P. gunni* suggests this was not expanded dorsally into a dorsal fontanelle (see above).

Johanson *et al*. (2017) interpreted lineations behind their ‘postorbital process’ as evidence of a ventral cranial fissure. We interpret this ‘fissure’ as the compressed junction of a separate element (the ‘gammation’) crushed against the neurocranium. Hirasawa *et al*. (2022) proposed a complete intracranial joint at this level, as in various sarcopterygians, a radical new interpretation based on only two CT-scanned specimens. Such a major structure has never been interpreted in the many hundreds of previously studied *P. gunni* specimens, and there is no evidence for it in the *P. australis* neurocranium. We interpret this as a post-mortem fracture through the weakest part of the neurocranium, the juncture between the more robust otic capsules, and the anterior unroofed pre-otic division, this level also weakened by the large trigeminal foramen, and therefore liable to collapse with compaction.

The neurocranium of *P. australis* reveals a single cranial fissure, preserved on each side as a deep groove of coalesced pores behind the interpreted hypophysial fossa, passing up into the optic nerve foramen. For this structure, of uncertain homology, we use the non-committal term **transverse fissure** (trans.f, Supplementary Fig. S3b). It originates ventrally as a deep cleft behind the hypophysial fossa (fo.hyp). This position resembles the ‘ventral otic/cranial fissure’ of early osteichthyans (Gardiner & Bartram 1977, Gardiner 1984), and the ‘stem chondrichthyan’ *Pucapampella* (Maisey 2001b, Brazeau & Friedman 2014). This approximates the embryonic boundary between the parachordal and trabecular/polar cartilage regions of the basicranium (generally anterior to the end of the notochord, and just behind the transverse pituitary vein and entry of the internal carotids). The ventral cranial fissure of basal actinopterygians may correspond with the ventral part to the sarcopterygian intracranial joint (Gardiner 1984); in other gnathostome groups it is more posteriorly situated, near the level of the posterior orbital wall, trigeminal nerve foramen, pituitary canal and basipterygoid process (e.g., Maisey 2001b, fig. 16.6, Brazeau & Friedman 2014, fig. 9).

By contrast, the transverse fissure in *P. australis* is relatively far forward, like the ‘optic fissure’ of various ‘placoderms’, which also passes into the optic nerve foramen. The placoderm optic fissure is also anterior to the entry points for the internal carotids, and some distance in front of the level of the pituitary vein. However, the ventral section of the placoderm optic fissure passes in front of the hypophysial fossa, not behind as in *P. australis*. Similar variation is seen in the cranial nerve branches exiting through the intracranial joint of sarcopterygians (profundus in osteolepiforms and porolepiforms, trigeminal in actinistians; see Schultze 1987, Janvier 1996, fig. 6.4), leading to diverse interpretations of homologies (e.g., Bjerring 1978).

In summary, the transverse fissure that we identify in the holotype of *P. australis* has a unique morphology, resembling in some respects the ventral otic fissure of osteichthyans (including the sarcopterygian intracranial joint), and in others the optic fissure of placoderms.

**Cranial nerves**

Two major openings pierce the lateral wall of the neurocranium (Fig. 1e). The smaller anterior opening for the optic nerve (II, Supplementary Figs. S3b, S5b) is anterodorsally notched on both sides (opa/acv). The optic artery and/or anterior cerebral vein have similar positions in *Chlamydoselachus* (Allis 1923, fig. 8) or *Cladodoides* (Maisey 2005, fig. 6). The recent interpretations of *P. gunni* based on CT data (Johanson *et al*. 2017, Hirasawa *et al*. 2022) proposed two other positions for the optic nerve foramen, but neither is correct. Johanson *et al*. (2017, fig. 1d) stated that their presumed postorbital process was ‘associated with a foramen for cranial nerve II (optic nerve) in the sidewall of the chondrocranium’, but this structure is the ‘gammation’, and not a neurocranial structure (see above). Hirasawa *et al*. (2022, fig. 3c) suggested a position for the optic nerve by an arrow over the lateral wall of the dorsal fontanelle, but any canal or opening is unclear, and this is a more anterior position compared to the clearly preserved optic nerve foramen on both sides of the *P. australis* holotype.

The larger posterior foramen in the *P. australis* holotype (V+VII, Fig. 1e, f) has the same level antero-posteriorly as the only cranial nerve foramen originally identified in *P. gunni* (arrow, Supplementary Fig. S2a), previously interpreted as either for the optic nerve (Sollas & Sollas 1904, Bulman 1931), or for the trigeminal and facial nerves (Moy-Thomas 1940, p. 397). Our new evidence supports Moy-Thomas’s interpretation that this is the trigemino-facialis foramen. The more complete right foramen (Fig. 1f, Supplementary Fig. S5b) suggests a posterodorsal notch, with a posterior groove and ridge above (r.h2, Supplementary Fig. S3b). These could have transmitted the mandibular branch of nerve V or the hyomandibular branch of nerve VII, both needing to pass posterolaterally behind the jaw joint.

Regarding the olfactory nerve (I), olfactory tracts traversing the cranial walls would have left openings of significant size. Paired anterior openings on either side of the median ventral septum (?I, Fig. 1c, d, e) correspond to those interpreted as olfactory nerve foramina in *P. gunni* by Johanson *et al*. (2017). This would imply backward deflection of the olfactory tracts to reach the structures called ‘hemidomes’ by Sollas & Sollas (1904), and interpreted by them and some other authors (Thomson *et al*. 2003, Johanson *et al*. 2017, fig. 1f; ‘elements 1-5’) to be mineralised parts of the nasal capsules.

Alternatively, Moy-Thomas (1940) considered the same ‘hemidome’ structures to represent parts of the palatoquadrate (HE, Supplementary Fig. S2d), and proposed that the nasal sacs of *Palaeospondylus* were positioned anteriorly ‘between the ventral rostralia’, divergent olfactory tracts being suggested by the diverging lateral rostralia (LR, Supplementary Fig. S2d). Moy-Thomas (1940, p. 397) noted variation in the number of dorsal rostralia (DR) in different *P. gunni* specimens (between 4 and 6), suggesting they could be preservational artefacts of an originally continuous sheet with regular thickenings (perhaps comparable to fluting preserved within ossified nasal capsules of some placoderms; e.g. Ritchie 2004, fig. 3; Goujet & Young 2004, fig. 6C). In their CT-scanned specimen, Hirasawa *et al*. (2022, suppl. fig. 2) interpreted three dorsal rostralia on each side as the roof of left and right nasal capsules, their ‘postnasal wall’ corresponding to the ‘ampyx’ of previous authors (AM, Supplementary Fig. S2d). The structure of the *P. australis* holotype confirms that under either the ‘hemidome’ or anterior nasal sac interpretation, the nasal capsules were not integrated with the rest of the neurocranium. However the lateral expansion of our interpreted ‘precerebral fontanelle’ would be consistent with anteriorly diverging olfactory tracts (prc.font, Supplementary Fig. S2b). Comparisons can be made with some Palaeozoic chondrichthyans, where the nasal capsules are preserved as anteriorly open cups on either side of the precerebral fontanelle (e.g., Schaeffer 1981), the olfactory tracts leaving only grooves on each side (e.g., *Cladodoides*, Maisey 2005).

New evidence is provided by the *P. australis* holotype for the paired foramina interpreted by Johanson *et al*. (2017) to have transmitted the olfactory nerves. These penetrate the ‘tauidion’ (fo7, Supplementary Figs. S2, S3). They open ventrally into conspicuous lateral grooves (l.gr), which meet posteriorly in the midline in a notch (n3) behind the median ventral septum. This seems an unlikely morphology for transmitting the olfactory nerves back to paired nasal capsules. Whatever nerves and/or vessels were contained in these conspicuous openings, they traversed the anterior floor of the cranial cavity near the midline, then passed downwards and backwards into the anterior part of the orbital cavity. Possible equivalents are the small canals passing back from the nasal capsules in *Squalus* (e.g., Schaeffer 1981, fig. 15), the orbitonasal and profundus canals of *Youngolepis* (Chang 1982, fig. 17), or the ‘nasobasal canals’ of various osteichthyans, which Gardiner (1984, p. 267) concluded transmitted maxillary, buccal or profundus nerve branches. Arteries (maxillary branch of the orbital) and/or veins (orbitonasal) may also have passed through them. Hirasawa *et al*. (2022) interpreted these foramina as transmitting the anterior cerebral veins.

For the remaining cranial nerves, the only evidence is a communication from the cranial cavity into the otic capsule shown by a horizontal slice near the neurocranial floor (VIII, Supplementary Fig. S6b), possibly for the auditory nerve. The oculomotory nerves (III, IV, VI) normally enter the orbit through smaller foramina, of which there is no clear evidence in the *P. australis* holotype. There is also no indication of the position of the glossopharyngeal (IX) or vagus (X) cranial nerves, the latter presumably emanating through the non-preserved oticoccipital fissure, as suggested by Hirasawa *et al*. (2022, fig. 3a).

**Cranial arteries**

The cranial vascular system has been almost completely unknown for *Palaeospondylus*. The *P. australis* holotype is the first and only specimen showing clear vascular structures. The median ventral depression shows many foramina, and two pairs of longitudinal grooves (gr.vasl, gr.vasm, Supplementary Figs. S3a, S4b, S5a-b) that contain small foramina piercing the basicranium, suggesting blood supply to the brain.

For craniates, the fundamental position of the cranial arterial system is ventral to the basis cranii (Goodrich 1930, p. 514). The interpreted vascular grooves on the lateral sloping walls of the median longitudinal depression in *P. australis* are therefore assumed to have been morphologically ventral to the notochord, or perhaps somewhat lateral. This position would essentially correspond to the Early Devonian *Gydoselache*, where both the notochord and dorsal aortae are preserved beside each other as canals within the parachordal plate (Maisey *et al.* 2019, fig. 5.4H-I).

The preserved pattern of foramina and grooves, summarised in Supplementary Figure S3a, has been interpreted mainly by comparison with Palaeozoic chondrichthyans. However, these show considerable diversity in braincase morphologies (e.g., Coates & Sequeira 1998, figs. 6–7), and even after decades of investigation, interpretation of the basicranial arterial pattern in some taxa is still uncertain (e.g. Maisey 2005, fig. 35). For *Palaeospondylus* therefore, this first description and interpretation of the cranial vascular system is very provisional.

The longitudinal vascular grooves extend some two thirds the length of the neurocranium (Supplementary Fig. S3a), from paired posterior foramina facing ventromesially (fo1, fo2), to an anterior notch (n2) in front of the suborbital ridge. A posterior branch of the mesial groove goes to another notch (n1) beside the posterior median process. The lateral longitudinal groove (gr.vasl), fully preserved only on the left side (Supplementary Figs. S4b, S5b), is most distinct posteriorly, with indications of a mesial branch connecting to the mesial vascular groove. From its originating posterior foramen (fo2) smaller dorsal and dorsolateral grooves seem to pass onto the posterior neurocranial wall (gr.d, gr.dl, Supplementary Fig. S4b).

Small irregular central openings (Supplementary Fig. S6b) are probably artefacts (gaps in the thin neurocranial floor), but the most anterior (fo3, Supplementary Fig. S5a) suggests two lateral notches from faint converging rows of pores, reminiscent of the converging grooves to a single median internal carotid foramen in Palaeozoic chondrichthyans (e.g., *Xenacanthus* sp., Schaeffer 1981, figs. 5-6; Coates & Sequeira 1998, figs. 6-7). Scattered pores across the midline suggest a vascular commissure (comm), but otherwise the middle part of the longitudinal depression (gr.not) is relatively smooth compared to the denser pore distribution laterally (Supplementary Fig. S5).

In the Devonian chondrichthyans *Cladodoides* and *Doliodus* (Maisey 2005, fig. 21; Maisey *et al.* 2009, fig. 3) the lateral dorsal aortae are enclosed within the basicranial floor between two pairs of foramina. The posterior foramina are behind the level of the oticoccipital fissure, so would not be shown by the *P. australis* holotype. Paired ventral foramina in *P. gunni* labelled ‘lda’ (Johanson *et al*. 2017, fig. S1B, D), if not gaps between the first occipital arch crushed against the neurocranium, could correspond to the posterior paired openings in the occipital segment of these Palaeozoic chondrichthyans. The openings in *P. gunni* evidently also partly correspond to the ‘vestibular fontanelles’ claimed by Hirasawa *et al*. (2022).

In *Cladodoides* the anterior foramina open into converging ventral grooves, a short lateral branch interpreted as for the efferent hyoid artery (Maisey 2005, fig. 5). The converging grooves branch anteriorly, interpreted as for a lateral orbital artery and mesial internal carotid. The internal carotid is assumed to re-enter the neurocranium near the midline beside the hypophysial fossa, as in various other Palaeozoic chondrichthyans. This is a morphological association that applies to most vertebrates (Rahmat & Gilland 2014). For the Early–Middle Devonian pucapampellids re-entry of the internal carotids is interpreted as widely separated in Bolivian material (Maisey 2001b, fig. 16.5), or close to the midline through a bilobed median hypophysial opening in the South African specimen (Maisey & Anderson 2001, *Gydoselache*: Maisey *et al.* 2019, fig. 5.4B). In *Cladodoides* and ‘*Cobelodus*’ the lateral dorsal aortae also exited through paired foramina in the parachordal plate, then bifurcate into lateral (orbital artery) and mesial (internal carotid) branches (Maisey 2005, 2007). A long external course for the orbital artery seems a chondrichthyan feature compared to other early gnathostomes (e.g., Giles *et al.* 2015, fig. 2).

In the holotype of *P. australis*, lacking the occipital portion, the longitudinal vascular grooves originating from double paired foramina (fo1,2) might indicate that each lateral dorsal aorta bifurcated internally just before its exit, in contrast to *Doliodus*, *Cladodoides*, and ‘*Cobelodus*’, where it subdivided just after its exit. This provisional interpretation would imply that the lateral vascular groove of *P. australis* carried the orbital artery, and the mesial groove the internal carotid artery. However, there is no indication of a lateral branch for an efferent hyoid artery.

The indistinct anterior continuation of the lateral vascular groove (gr.vasl) aligns with the vascularised surface just inside the ventrally projecting suborbital ridge (r.so, Supplementary Fig. S3a). A large foramen (fo4) through the subocular shelf and into the orbit could have contained an anteriorly directed and largely external orbital artery. By comparison, the orbital artery of *Cladodoides* and ‘*Cobelodus*’ was more external, only notching the edge of the subocular shelf (Maisey 2005, 2007), whereas in *Doliodus* it evidently was enclosed for most of its passage into the orbit (e.g., Giles *et al.* 2015, fig. 2).

Within the orbit of *P. australis* the corresponding opening (fo5, Supplementary Figs. S3b, S6b-c) is associated with several grooves crossing the subocular shelf. The orbital artery of lower gnathostomes (which evolved into the stapedial artery of tetrapods) originally comprised supraorbital, infraorbital and mandibular branches (Goodrich 1930, fig. 547), so a prominent posterolateral groove could have carried the mandibular branch (as identified by an equivalent groove in *Buchanosteus*; Young 1979). The anterior maxillary branch (‘external carotid’) in *Chlamydoselachus* runs forward to pass beneath the nasal capsule (Allis 1923, fig. 55). For *Palaeospondylus* it is also possible that the paired openings through the ‘tauidion’ (fo7) may have carried this branch.

The mesial vascular groove of *P. australis* (gr.vasm), interpreted to have carried the internal carotid, extends anteriorly between paired ‘suborbital ridges’ as restored (r.so, Supplementary Fig. S3a). The suborbital ridge is only preserved on the more complete left side of the holotype, and is presumed to be lost on the right side due to abrasion (see above). This irregular structure displays a more distinct lateral notch (n.epsb), with associated rows of pores running mesially (these preserved on both sides; Supplementary Fig. S5a). These structures can be interpreted to represent the passage of the efferent pseudobranchial artery to its junction with the internal carotid through this lateral notch (n.epsb, Supplementary Fig. S3a-b). The associated grooves and rows of pores suggest that this junction was extramural, as in placoderms and osteichthyans. A similar lateral notch in *Gydoselache* may have transmitted the efferent pseudobranchial in the opposite direction (Maisey & Anderson 2001, fig. 1). The corresponding lateral notch of *Cladodoides* (Maisey 2005, fig. 21), situated well in front of the internal carotid entry and hypophysial fossa, implies that the efferent pseudobranchial must have had a dorsal junction, embryonically dorsal to the trabeculae, as in modern elasmobranchs. In osteichthyans this junction is ventral, a condition is also indicated in the ‘stem gnathostome’ *Janusiscus* (Giles *et al.* 2015), where a lateral groove turns back towards the palatobasal connection (but evidently in front of it); a similar morphology is seen in several ‘placoderms’ (Young 1986, p.18). The association with the palatobasal connection to the palatoquadrate is variable in buchanosteid arthrodires -- the efferent pseudobranchial groove passes behind it in some specimens (Hu *et al.* 2017), or between double articular facets in others (Young 1986, fig. 9A). The efferent pseudobranchial notch in *Cladodoides* is also associated with a flange for the palatobasal articulation. The arterial interpretation for this region just presented therefore suggests a palatobasal connection (possibly double) for *P. australis* (see below).

An efferent pseudobranchial/internal carotid junction dorsal to the trabeculae was proposed as a chondrichthyan synapomorphy (Schaeffer 1981, Coates & Sequeira 1998), but may be apomorphic only for elasmobranchs (Maisey 2001b, Maisey & Anderson 2001). Maisey (2005, p. 59) proposed that the efferent pseudobranchial passed through the trabeculae primitively in gnathostomes, a condition conserved in osteichthyans, and stem chondrichthyans like the Early Devonian *Gydoselache*.

The internal carotid groove of *P. australis* continues anteriorly to the same notch in front of the suborbital ridge as the lateral vascular groove (n2, Supplementary Fig. S2d-e). If the internal carotid turned up over this notch, it may have followed a slight groove running anteromesially towards the transverse fissure (gr.ic, Supplementary Fig. S3b). There are paired foramina just behind the transverse fissure (fo6), and larger openings formed the fissure itself (Supplementary Fig. S5a). We provisionally interpret the internal carotids to have entered the cranial cavity close to or within the transverse fissure (f.ic). A single midline entry for the internal carotids behind the hypophysial fossa is considered a derived condition for chondrichthyans (Coates & Sequeira 1998, Davis *et al.* 2012, character 78). In contrast, Hirasawa *et al*. (2022) labelled widely-spaced openings for the internal carotids. The position of these is difficult to locate on our uncrushed neurocranium, where no comparable foramina are apparent, and the *P. australis* holotype suggests that internal carotid entry was much closer to the midline. A lateral branch of the internal carotid could have passed in the same groove back into the orbit, representing the ophthalmic artery. Alternatively, this may have branched internally, like various placoderms and osteichthyans where the ophthalmic artery reached the orbit through a foramen in a ventral myodome (Young 1986, Clement *et al.* 2018). Nevertheless, these interpretations remain very provisional for *P. australis*.

Summarising the above interpretation, the cranial arterial circuit for *Palaeospondylus* would have comprised **lateral dorsal aortae** entering the occipital division through paired foramina (on the evidence of *P. gunni*), and were thus enclosed in canals within the ‘basioccipital’ region (character 30 of Brazeau & Friedman 2014). These divided internally into lateral (orbital artery) and mesial (internal carotid) branches, which emanated onto the ventral surface of the neurocranium just anterior to the oticoccipital fissure. Both arteries were then extramural across the basicranium (a branching point for the **efferent hyoid artery** is not identified). The **orbital artery** entered the orbit via a foramen (fo4) and short canal traversing the suborbital ridge, its mandibular branch possibly indicated by a posterior groove on the subocular shelf, and its maxillary branch possibly passing anteriorly through a foramen (fo7) in the ‘tauidion’. The **internal carotid** received the **efferent pseudobranchial artery** externally, through a lateral notch (n.epsb) in the suborbital ridge associated with a double basipterygoid process. The internal carotid passed dorsally over an anterior notch (n2), turning anteromesially to enter the cranial cavity close to or within the transverse fissure. A posterior branch into the orbit for the **ophthalmic artery** may have been external or internal.

It is noted that two features of *Palaeospondylus* just described (fo3, comm, Supplementary Fig. S3a) are not seen in the basicranial arterial system of other early gnathostomes. Grooves and foramina suggest possible mesial arterial branches that entered the cranial cavity through a median opening (fo3), but this seems too posterior to represent the internal carotids, and foramina across the midline (comm) seem to indicate a median connection between internal carotids at the level of the efferent pseudobranchial notch.

**Cranial veins**

As in all gnathostomes, the cranial venous system in *Palaeospondylus* presumably comprised equivalents of the cerebral, ophthalmic, and pituitary veins, all draining laterally into a jugular vein. According to classic comparative morphology (Goodrich 1930, p. 533) the main head vein (vena capitis medialis) in front of the level of the trigeminal nerve is positioned on the inner side, but behind this is represented by the vena capitis lateralis (jugular vein), which passed posteriorly on the outside of the otic capsule. The connection between the two through the lateral cranial wall (pituitary vein) is associated with exit of the facial nerve.

Previously for *Palaeospondylus*, Johanson *et al*. (2017, fig. S1D, S4) identified jugular canal openings in the ‘postorbial process’ of *P. gunni*, but as noted above the interpreted ‘postorbital process’ of *P. gunni* is not neurocranial. Hirasawa *et al*. (2022) suggested the paired foramina through the ‘tauidion’ (fo7, Supplementary Figs. S2c, S3) transmitted the orbitonasal veins, an interpretation consistent with the relatively large size of those foramina, with associated lateral grooves and a posterior notch in the *P. australis* holotype suggesting a midline connection of the contained vessels (n3, Supplementary Fig. S3). Allis (1923, p. 203) briefly noted that in *Chlamydoselachus* the two orbitonasal veins were connected by a commissure, which would be consistent with this morphology.

It is very uncertain where the pituitary vein opened in the *P. australis* holotype. A space in the posteroventral corner of the orbit beneath the large trigemino-facialis foramen (v.pit, Supplementary Fig. S6c-d) could correspond to the large pituitary vein opening in various ‘placoderms’ (e.g. Young 1986). However, an anterior or anterodorsal position relative to the basipterygoid process is seen in various Devonian osteichthyans (Chang 1992, Gardiner 1984, Lu *et al.* 2012), so the pituitary vein could have emanated through more anterior foramina (fo8 or fo9), placing it closer to the level of the transverse fissure. We note that any position somewhat behind our interpreted hypophysial fossa would be anomalous.

The pituitary vein must have connected posteriorly with the jugular vein. Typically, stem gnathostomes have the jugular vein enclosed in a jugular canal traversing the base of a lateral projection of the neurocranial wall behind the orbit (‘postorbital’ or ‘lateral otic’ process of Giles *et al.* 2015). For *Palaeospondylus* *australis* there is no postorbital process, and the XCT data show no indication of an internal jugular canal, with the otic capsule completely occupied by the labyrinth cavity (Fig. 2a, b). Similarly, the labyrinth cavity in *P. gunni* (Johanson *et al*. (2017, fig. 2; Hirasawa *et al*. 2022, fig. 3c, suppl. fig. 5a) shows no evidence of an internal jugular canal in the XCT data.

This suggests that the jugular vein remained external. Both sides of the *P. australis* holotype have a broad shallow groove across the anterolateral face of the otic capsule, which we can interpret to have carried the jugular vein posteriorly from the orbit. This could have connected with the pituitary vein opening, either directly if it was placed in the posteroventral corner of the orbit (gr.jv, Supplementary Fig. S6c-d), or beneath the horizontal ridge (r.h1, Supplementary Figs. S3b, S6b), if the pituitary vein opened more anteriorly as in osteichthyans. The course of the jugular vein further posteriorly remains uncertain. It may have passed through a ventral notch (n.jv, Supplementary Fig. S6c-d) going beneath a possible articular facet at the lateral angle of the neurocranium (n.jv, art.al, Supplementary Fig. S3b). If that structure was not an articular facet, the jugular vein may have continued posteriorly, passing along a groove above a second (posterolateral) articular facet (art.pl, Supplementary Fig. S3b), and across the posterolateral face of the otic capsule. In most other stem gnathostomes the posterior section of the jugular vein is external, in a groove across the posterolateral face of the otic capsule. However, a jugular groove across the anterolateral face seems unique to *P. australis*.

Another possibility to be considered, noting that a postorbital process pierced by a jugular canal is lacking in the *P. australis* neurocranium, is that the separate ‘gammation’ of *P. gunni* (GA, Supplementary Fig. S2d), interpreted as containing a jugular canal, might be a homologue of the neurocranial postorbital process/lateral commissure. The embryonic lateral commissure is a neurocranial structure in most gnathostome groups, interpreted as originally a visceral arch structure (mandibular or hyoid arch), which later accreted to the neurocranium, thereby enclosing the jugular vein in a canal. If the ‘gammation’ of *Palaeospondylus* was a lateral commissure/postorbital process not yet incorporated into the neurocranium, this would also be a unique adult condition.

**Articulation surfaces on the neurocranium**

New evidence for articulation or attachment surfaces can contribute to previous discussions of the jaws and visceral arch elements for *P. gunni*. Due to incomplete preservation of the *P. australis* holotype, some of the structures identified here are only observed on one side, so our interpretations are very provisional. We show them as paired structures on the restorations, to facilitate comparison with more completely preserved neurocrania.

Such structures could represent connections with upper cartilages of the jaws or branchial arches, but could also represent sites for tendinous or muscle insertions. They have generally been recognised as porous depressions or openings that are partly or completely surrounded by raised rims. Given the porous surface texture of the neurocranium as a whole, identifying such structures is very provisional, but all are shown on the restorations for completeness.

The following five possible articular surfaces are illustrated for the neurocranium of *P. australis*:

1. a laterally-directed possibly double ‘basipterygoid’ process on the subocular shelf (bpt, Supplementary Figs. S3a, S5a);
2. an articular depression on the mesial part of the pre-otic ridge, facing anteroventrally and slightly laterally (art.av, Supplementary Figs. S3a, S5a);
3. an articular depression above and anterior to the lateral angle of the otic region, facing anterolaterally (art.al, Supplementary Figs. S3b, S6c);
4. an articular depression below and behind the lateral angle, facing posterolaterally (art.pl, Supplementary Figs. S3b, S6c);
5. an articular depression inside the posteroventral angle of the otic region, facing posteriorly and slightly laterally (art.pv, Supplementary Figs. S3, S4b).

Articular surface (i) is proposed on the evidence of a lateral projection (possibly double) from the suborbital ridge closely associated with the notch and groove interpreted above as for the efferent pseudobranchial artery. This would imply a ‘palatobasal’ connection with the palatoquadrate in *P. australis*. For *P. gunni* Moy-Thomas (1940, p. 398) noted that the ‘hemidomes … apparently articulate with the side walls of the neurocranium, anteriorly to the large nerve foramen’. He considered the hemidomes to be part of the palatoquadrate, so this could correspond to our proposed palatobasal connection in *P. australis*, which is also just anterior to the level of the trigemino-facialis opening (Supplementary Fig. S3).

Of the three standard connections between the palatoquadrate and the braincase, Gardiner (1984, p. 301) maintained that palatobasal and otic connections were primitive for gnathostomes, and Maisey (2005) concluded that ethmoidal and palatobasal connections were primitive for chondrichthyans. Moy-Thomas (1940, p. 398) suggested that in *P. gunni* an anterior palatoquadrate ossification (‘hemidome’) articulated with the ‘ampyx’ (HE, AM, Supplementary Fig. S2d), which would represent an ethmoidal connection. However, *P. australis* shows no evidence of an anterior articulation on the neurocranium, with clearly no articular surface on the lateral process of the ‘tauidion’ (Supplementary Fig. S6e-j).

Articular surface (ii) is provisionally identified in the same position as that previously restored for *P. gunni* (art.av, Supplementary Fig. S2c), but, as noted above, the restored depression in *P. australis* (art.av, Supplementary Fig. S3a) is only seen on the right side of the holotype (?art, Fig. 1). According to Moy-Thomas (1940, fig. 4) this articular surface in *P. gunni* received a posterior articular process of the ‘gammation’, considered by him as also part of the palatoquadrate. This position on the neurocranium in both species would be consistent with an otic palatoquadrate connection.

Articular surface (iii), a possible anterolateral articulation (art.al, Supplementary Fig. S3b) occupies the posterior end of the groove interpreted above as for the external jugular vein (Fig. 1e). It is rimmed above and below; an anterior rim, separating it from the jugular groove, may be an artefact (Supplementary Fig. S6c). If an actual articular facet, this surface is roughly horizontal, and faces anterolaterally, so is unlikely to have received an epibranchial visceral arch element.

Articular surface (iv) faces mainly laterally, but also slightly posteriorly and ventrally (art.pl, Supplementary Fig. S3b). It is interpreted as a roughly triangular articular facet, broadest dorsally with a distinct rim, and with its long axis steeply inclined. This immediately suggests the oblique hyomandibular articular facet on the lateral commissure of various early osteichthyans (e.g., Chang 1982, Gardiner 1984, Lu *et al.* 2017, 2019). This could have received the hyomandibula that we have identified for *P. australis* (Fig. 1h), but if so the articulation would be much further posteriorly than the hyoid arch elements identified by Moy-Thomas (1940) or Johanson *et al*. (2017) for *P. gunni*. However Hirasawa *et al*. (2022) proposed an element even farther back as a posteriorly directed ‘hyomandibula’–the ‘post-occipital lamellae’ of other authors (PL, Supplementary Fig. S2d). The ‘hyomandibulae’ of Hirasawa *et al*. are posteriorly directed, with no connection to ventral elements of the hyoid arch, and far removed from their interpreted mandibular arch elements. As noted above, the putative facet for their proximal articulation on the neurocranium does not exist (a separate visceral arch element; BA, Supplementary Fig. S2d). These conspicuous rod-like elements in *P. gunni*, projecting behind the head and flanking the anterior axial skeleton, have been subject to diverse interpretations by other authors (parts of the shoulder girdle, dipnoan cranial ribs etc.). Moy-Thomas (1940) suggested aberrant enlarged posterior branchial arches, and Johanson *et al*. (2017) proposed possible synarcuals incorporating vertebral centra, as in batoid chondrichthyans. Our interpretation of the *P. australis* holotype suggests that the socketed end of these ‘post-occipital lamellae’ likely articulated with articular surface (v), which faced posteriorly and slightly laterally (art.pv, Supplementary Fig. S3b).

The double posterodorsal process in *P. australis* (pd.pr, Supplementary Figs. S2c, S3-S4) could have had a non-articular connection with the first occipital arch. However, the corresponding neurocranial projection in *P. gunni* (e.g. Sollas & Sollas 1904, figs. 1, 3) is typically preserved well lateral of the vertebral column, although this was probably rotated out by compaction.

**IV. Description of other material referred to *Palaeospondylus australis* sp. nov.**

Over 400 isolated elements in the *P. australis* material were interpreted on histology to belong to *Palaeospondylus* before the holotype braincase was discovered (Burrow *et al.* 2014). Most are paired (asymmetric) elements. They are grouped into eleven morphological types (Supplementary Table S1). Suggested correspondence to elements of *P. gunni*, with various earlier interpretations and terminologies, are summarised in Supplementary Table S2.

**Type 1** elements (Fig. 1h) are relatively common in our material (Supplementary Table S1), with a distinctive morphology which we consider identifies them as ‘gammations’. They are flattish subrhombic structures with two articulation surfaces along one long edge, separated by a groove leading to a canal that opens out on the lower corner of the opposite edge. A semicircular embayment, presumed to be the cotylus for the other epal element articulation, extends for most of the length of the short edge below the canal.

We have also identified over 100 examples of other complex structures that may correspond to the ‘pre-gammations’ sensu Sollas & Sollas (1904), denoted **Type 2** elements (Fig. 3a, b). These relatively robust, sub-triangular elements are curved in two dimensions, longitudinally and transversely, have a double headed condyle at one end, a rounded swollen angle in the middle of the element, and possible articulation or connecting surfaces along two of the three sides.

**Type 3** elements (Fig. 3o, p) resemble the elements identified as hyomandibulae by Moy-Thomas (1940), being dog-bone shaped with a double headed condyle at one end and a wider thicker convex edge at the other end.

**Type 4** elements (Fig. 3c-e), represented by some 70 specimens, are identified as ceratohyals, as also recognised in *P. gunni* by Moy-Thomas (1940). They are flat subrectangular plates with a perpendicularly directed process at one end. A large depression on the basal surface below the process could have been for muscle or ligament attachment.

**Type 5** elements (Fig. 3m, n) may be equivalent to the ‘anterior trapezial bars’ of Sollas & Sollas (1904), being small, slightly sigmoidally-shaped elements.

**Type 6** elements (Fig. 3k, l), equivalent to the post-occipital lamellae of *P. gunni* (*sensu* Moy-Thomas 1940), are cylindrical for most of their length, with a socket articulation at the anterior end and a flattened spatulate shape at the other end.

**Type 7** elements (Fig. 3h, i) are hollow, three-sided pyramid-shaped structures with an arched base. We consider these comparable to the comma-shaped structures that are always preserved behind the braincase in *P. gunni*, near the anterior end of the post-occipital lamellae. The 3D preservation shows that a smaller canal branches off to the side, from the wide central cavity that opens out apically on the Type 7 elements. Thomson *et al.* (2003) identified these as occipital arches, an interpretation with which we concur, given that the isolated neurocranium lacks an occipital region.

**Type 8** elements (Fig. 3f) are vertebral centra (‘ring vertebrae’ sensu Thomson *et al*. 2003).

**Type 9** elements (Fig. 3g) are neural or haemal arches.

**Type 10** elements (Supplementary Fig. S1a) are short cylindrical bars which we are unable to correlate with structures in *P. gunni*; possibly they are branchial bars.

**Type 11** is only known from one incomplete specimen (Fig. 3q, r). It is bowl-shaped with a central foramen; three strut-like bars radiate out from the foramen to join the side of the ‘bowl’.

The asymmetrical elements are difficult to interpret reliably because of their fragmentary disarticulated preservation, and also because they show a more complex morphology, that is generally not preserved in comparable elements of *P. gunni*.

Based on the position of the corresponding elements in *P. gunni* and their actual structure as revealed by SEM and 3D scans of the isolated *P. australis* elements, we interpret the Type 1 elements as hyomandibulas or less likely, lateral commissures, Type 2 elements as palatoquadrates, Type 3 elements as interhyals or hyomandibulas (dependent on the identity of Type 1 elements), Type 4 elements as ceratohyals, and Type 5 elements as Meckel’s cartilages (Fig. 3j). As noted earlier, the latter elements were identified by Hirasawa *et al.* (2022) as basipterygoid processes on the neurocranium, but we agree with their identification by Thomson *et al*. (2003) as Meckel’s cartilages. Given the position of the ‘pre-gammation’ in articulation with the Meckel’s cartilages in *P. gunni*, and the robustness and morphology revealed by the 3D preservation of Type 2 elements of *P. australis*, we consider these can only be the palatoquadrates. Unlike previous authors, we consider the hyoid arch in *Palaeospondylus* could comprise three elements, a hyomandibula (‘gammation’), an interhyal/epihyal (‘hyomandibular’ of Moy-Thomas 1940), and a ceratohyal; alternatively, if the proximal element is rather a lateral commissure, then we would concur with the interpretation of Moy-Thomas (1940). Regarding the Type 11 element, the only structure in *P. gunni* that could be comparable is the ‘hemidome’ of Sollas & Sollas (1904), which they identified as the nasal capsule (also Thomson *et al.* 2003, Johanson *et al.* 2017). Moy-Thomas (1940) noted its evident ‘triradiate structure’, as indicated by our specimen, but he interpreted it as an anterior part of the palatoquadrate (as did Hirasawa *et al.* 2022). The ‘hemidomes’ are positioned behind the precerebral fontanelle, and as noted earlier, this would require extremely unlikely backward deflection of unknown olfactory tracts to reach them, if they were the nasal capsules. However, we are unable to propose an alternative interpretation. Figure 3s is a reconstruction of the possible layout of the neurocranium and all the isolated elements.

**V. Relationships of *Palaeospondylus***

Johanson *et al*. (2017) interpreted the post-occipital lamellae as possible synarcuals incorporating vertebral centra, as in batoid chondrichthyans. We find this a morphologically unconvincing interpretation. All of the 60 Type 6 (= postoccipital lamellae) in our material conform to the same shape as the ones we have figured, irrespective of their size, with no evidence for fusion with any other elements (e.g. centra). In *Palaeospondylus gunni* however, the ‘postoccipital lamellae’ (the name given by Moy-Thomas is retained, as we are unsure of what they actually are) are not closely associated with the vertebral centra (and certainly not ‘within’ the synarcual, cf. Johanson *et al*. 2017, p. 100). They are also usually oriented posteroventrally, away from the vertebral column, in rare specimens preserved in lateral view. Other characters they listed to associate *Palaeospondylus* with chondrichthyans are: precerebral fontanelle, foramina for lateral dorsal aorta in chondrocranium, palatoquadrate articulation to the ventral postorbital process and the latter enclosing the jugular vein, and absence of perichondral bone (as a stem chondrichthyan). Previously, Johanson *et al*. (2010, p. 414) had assessed the unusual histology of *Palaeospondylus* to represent a ”fossilised ontogenetic stage of endochondral bone, a type of bone characteristic of osteichthyan vertebrates”. Apart from the precerebral fontanelle, the other characters listed above are invalidated by the new evidence of *P. australis*, as are some of the ‘crown-group gnathostome’ characters they proposed (e.g., elongate telencephalon, shown to be short by *P. australis*). We have demonstrated above that most of the identifications of elements made by Hirasawa *et al*. (2022) are incorrect, and there is no support for a tetrapod affinity for *Palaeospondylus*. An interesting observation is that the more extensive phylogenetic re-analysis by Brownstein (2023), based on the data matrix of Brazeau *et al*. (2020) and many of the anatomical interpretations of Hirasawa *et al*. (2022), with recoding of some characters, recovered *Palaeospondylus* as a stem gnathostome. The response by Hirasawa & Kuratani (2023), who also used a modified version of the Brazeau *et al*. (2020) dataset, maintained their tetrapodomorph result! Our phylogenetic analyses show *Palaeospondylus* as a stem gnathostome, sister group to the chondrichthyans; coding of the proximal hyoid arch element as a hyomandibula or lateral commissure (and associated changed coding states) does not affect the result.

**VI. SI Figures and tables**


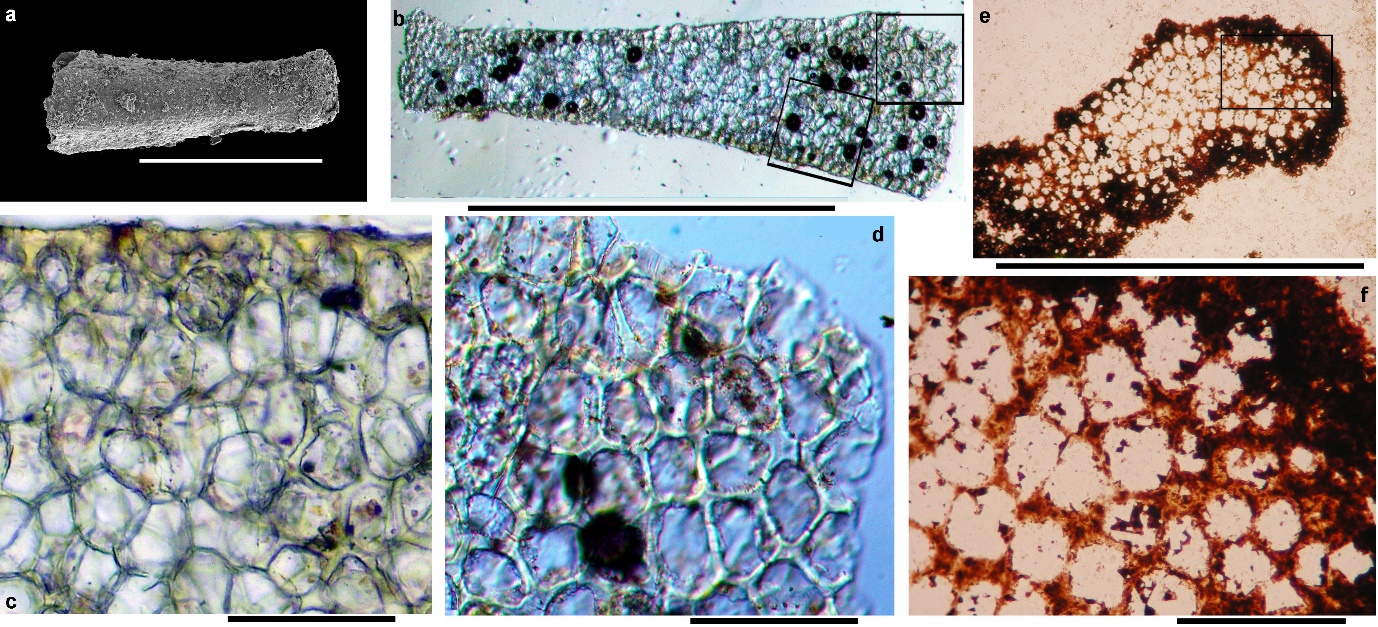


**SI Fig. 1. *Palaeospondylus* histological structure. a**, *P. australis* sp. nov., SEM image of isolated bar element QMF 53546. **b-d**, *P. australis* sp. nov., longitudinal thin section of isolated bar element QMF 53552; **b**, whole section; **c**, closeup of mid-element surface in lower box, rotated 180°; **d**, closeup of broken end of element in upper right box. **e**, **f**, *P. gunni*, probable palatoquadrate in thin section of whole animal NHMUK PV P.22393, closeup in (**f**) of area in box in (**e**) (see Johanson *et al*. 2012, fig. 2). Scale bars are 1 mm in a, b, e and 0.1 mm in c, d, f.


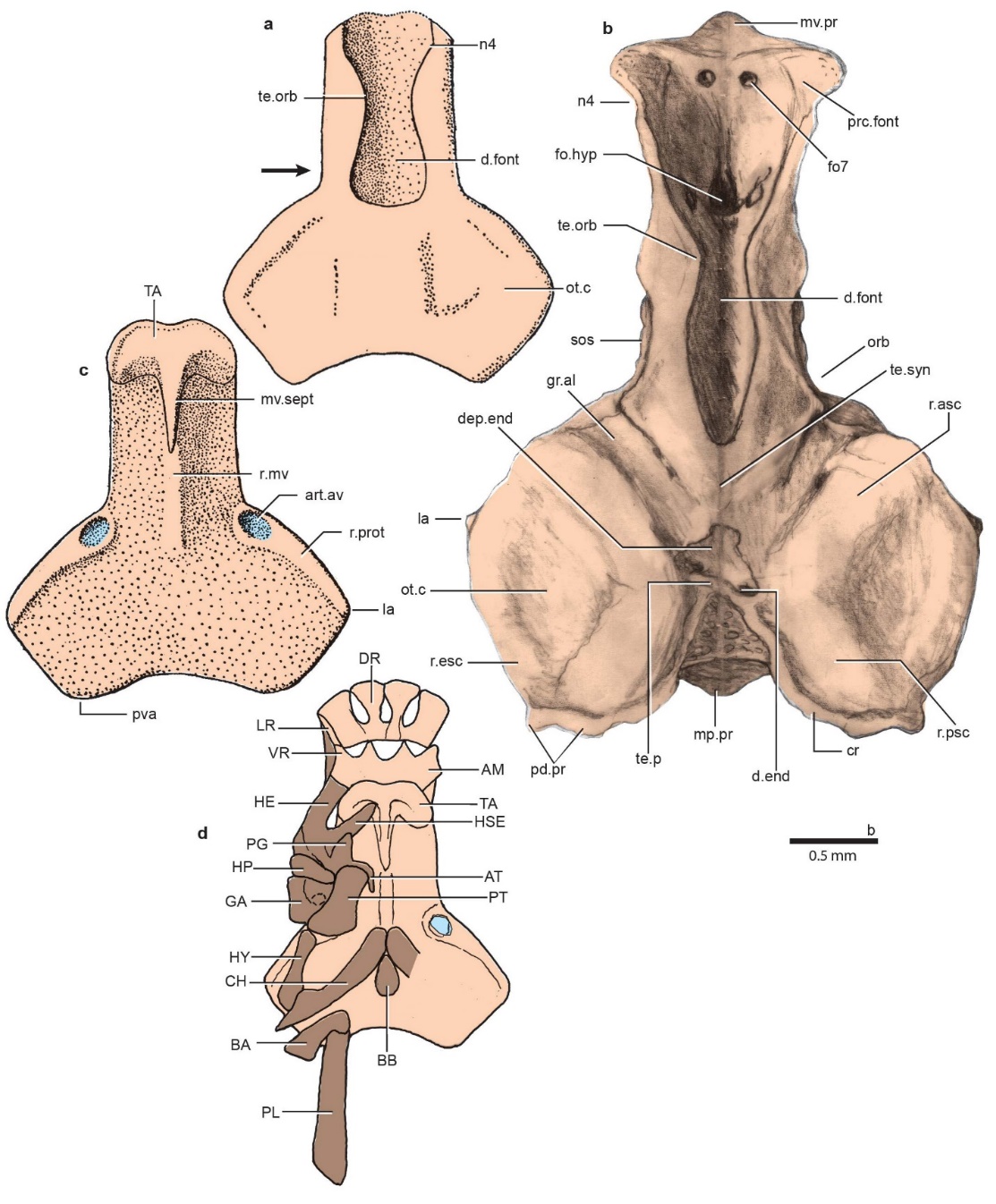


**SI Fig. 2. Neurocranial restorations for *Palaeospondylus*. a**-**b,** dorsal aspect for *P. gunni* Traquair (**a**) and *P. australis* sp. nov. (**b**). **c,** ventral aspect for *P. gunni* Traquair. **a**, **c** modified from Moy-Thomas (1940, figs. 1, 3) by removing structures (‘ampyx’, various ‘rostralia’^17^) anterior to the T-shaped ‘tauidion’, for valid comparison with the isolated *P. australis* neurocranium. Arrow in **a** shows position of the ‘foramen for fifth nerve’ identified by Moy-Thomas^1^. **d**, Ventral neurocranial restoration for *P. gunni*, including the ‘ampyx’ and ‘rostralia’, and right visceral arch elements (darker shading; left side of figure). Visceral arch positions after Moy-Thomas^1^, his labels providing a key to the terminology of Sollas & Sollas^17^ (based on Moy-Thomas 1940, fig. 2; shape, but not position, of some visceral arch elements updated from CT-scanned specimen of Johanson *et al*. 2017, fig. S1B.). (a, c, d, not to scale.) AM, ‘ampyx’; art.av, anteroventral articular depression; AT, ‘anterior trapezial bar’; BA, branchial arch; BB, basibranchial; CH, ceratohyal; cr, crest; d.end, endolymphatic duct opening; dep.end, endolymphatic depression; d.font, dorsal fontanelle; DR, ‘dorsal rostralia’; fo7, foramen through tauidion for cranial nerves or vessels; fo.hyp, hypophysial fossa; GA, ‘gammation’; gr.al, anterolateral groove; HE, ‘hemidome’; HP, ‘hemidome process’; HSE, ‘hemidome septum’; HY, hyomandibula; la, lateral angle of braincase; LR, ‘lateral rostralia’; mp.pr, posterior median process; mv.pr, anterior ventral median process; mv.sept, median ventral septum; n4, notch that may have transmitted cranial nerves or vessels; orb, orbit; ot.c, otic capsule; pd.pr, posterodorsal process; PG, ‘pregammation’; PL, ‘post-occipital lamella’; prc.font, precerebral fontanelle; PT, ‘posterior trapezial bar’; pva, posteroventral angle of braincase; r.asc, ridge over anterior semicircular canal; r.esc, ridge over external semicircular canal; r.mv, median ventral ridge; r.prot, pre-otic ridge; r.psc, ridge over posterior semicircular canal; sos, subocular shelf; TA, ‘tauidion’; te.orb, tectum orbitale; te.p, posterior tectum; te.syn, tectum synotum; VR, ‘ventral rostralia’.


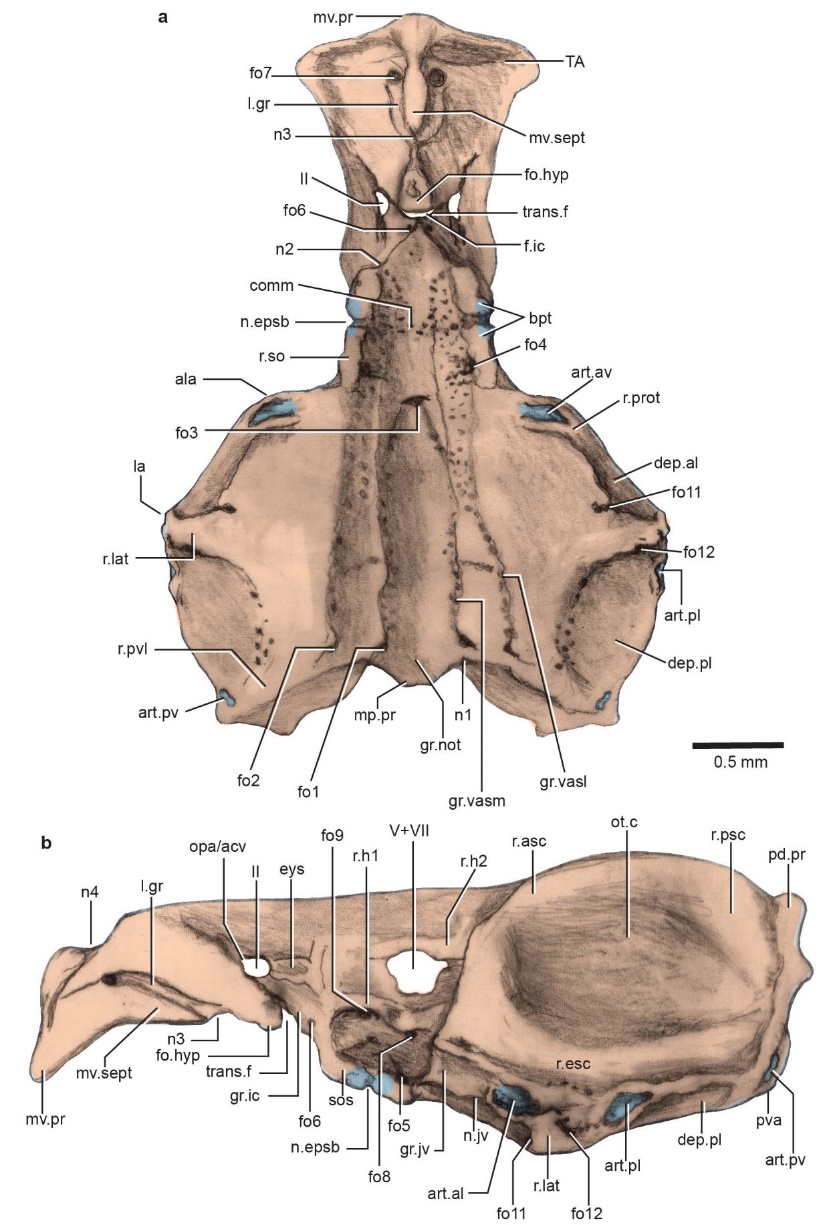


**SI Fig. 3. *Palaeospondylus australis* sp. nov.** Holotype neurocranium restored in ventral (**a**) and left lateral (**b**) views. II, foramen for optic nerve; V+VII, foramen for trigeminal and facial nerves; ala, anterolateral angle of braincase; art.al, anterolateral articular depression of braincase; art.pv, posteroventral articular depression of braincase; bpt, possible basipterygoid process; comm, possible arterial commissure; dep.al, anterolateral depression; dep.pl, posterolateral depression; eys, attachment area for an optic pedicle (eye-stalk); f.ic, foramen for internal carotid artery; fo1-12, foramina for cranial nerves or vessels; fo.hyp, hypophysial fossa of cranial cavity; gr.ic, groove for internal carotid artery; gr.jv, groove, possibly carrying the jugular vein; gr.not, notochordal groove; gr.vasl, lateral vascular groove; gr.vasm, mesial vascular groove; l.gr, lateral groove; n.epsb, notch for efferent pseudobranchial artery; opa/acv, notch, possibly for optic artery and/or anterior cerebral vein; r.h1, r.h2, lower and upper horizontal ridges in orbit; r.lat, lateral ridge under otic capsule; r.pvl, posterolateral ridge on ventral side of otic capsule; r.so, suborbital ridge; ‘tau’, T-shaped ‘tauidion’ region of braincase; trans.f, transverse fissure of braincase. (For other abbreviations see SI Fig. 2 caption.)

**
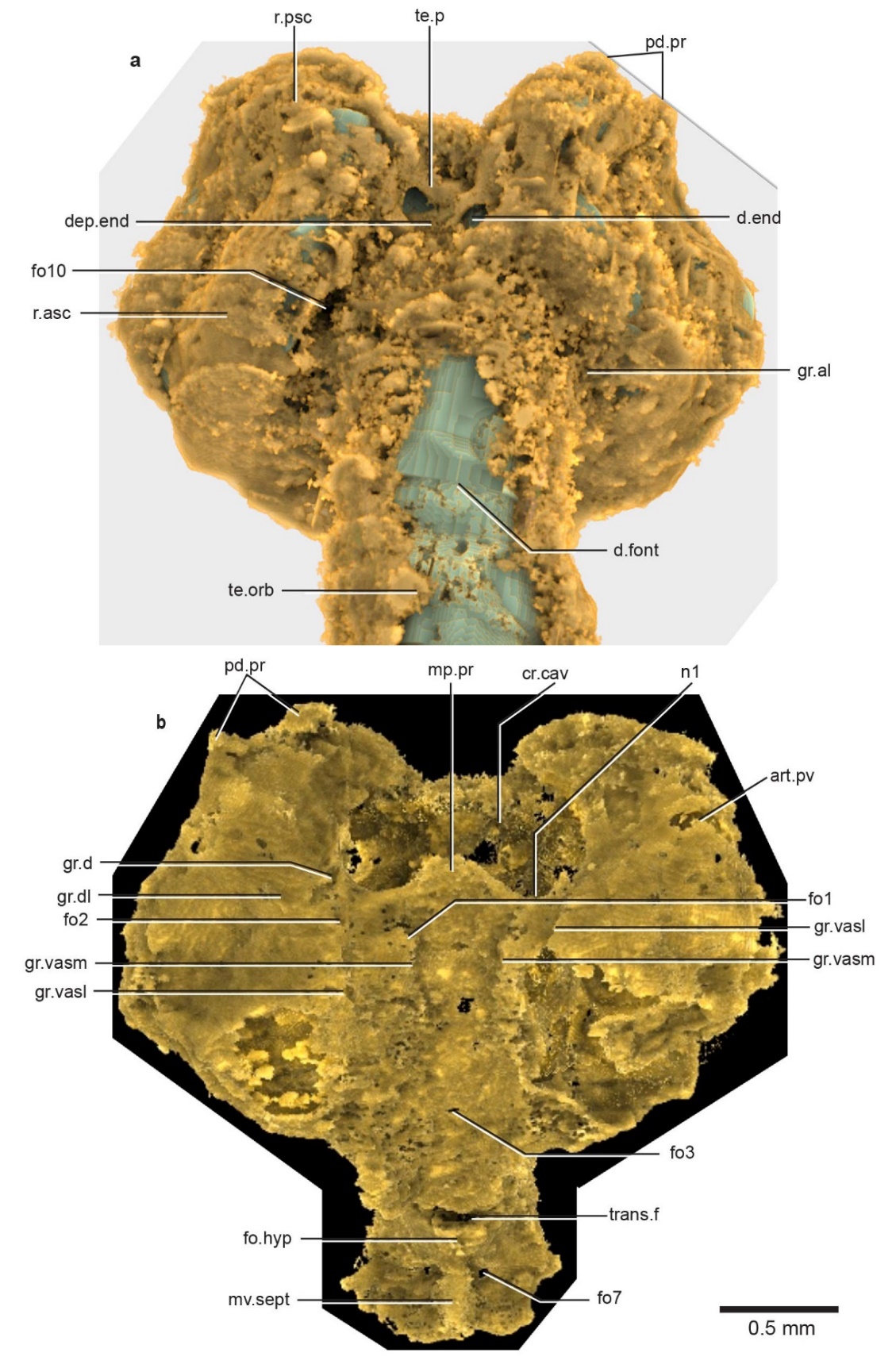
**

**SI Fig. 4. *Palaeospondylus australis* sp. nov.** Holotype neurocranium. **a**, Anterodorsal view of the otic region to show the endolymphatic duct openings and surrounding structures. **b**, Braincase floor in posteroventral view. cr.cav, cranial cavity; gr.al, anterolateral groove; gr.d, dorsal groove; gr.dl, dorsolateral groove. (For other abbreviations see SI Figs 2-3 captions.)


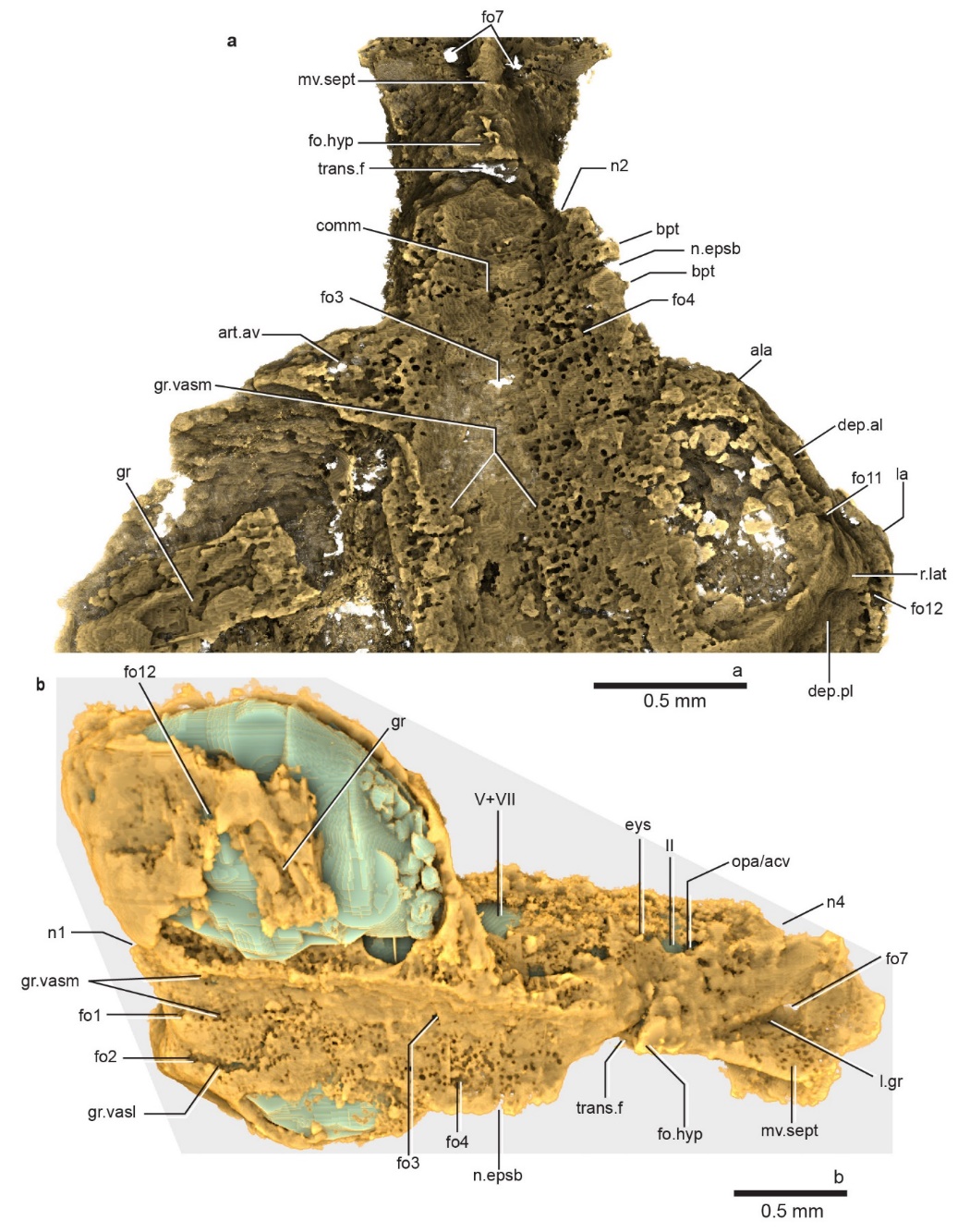


**SI Fig. 5. *Palaeospondylus australis* sp. nov.** Holotype neurocranium showing detail of vascular pores and grooves in a ventral view of the anterior part of the neurocranial floor (**a**), and the entire neurocranial floor in right ventrolateral view (**b**). gr, groove. (For other abbreviations see SI Figs 2-4 captions.)


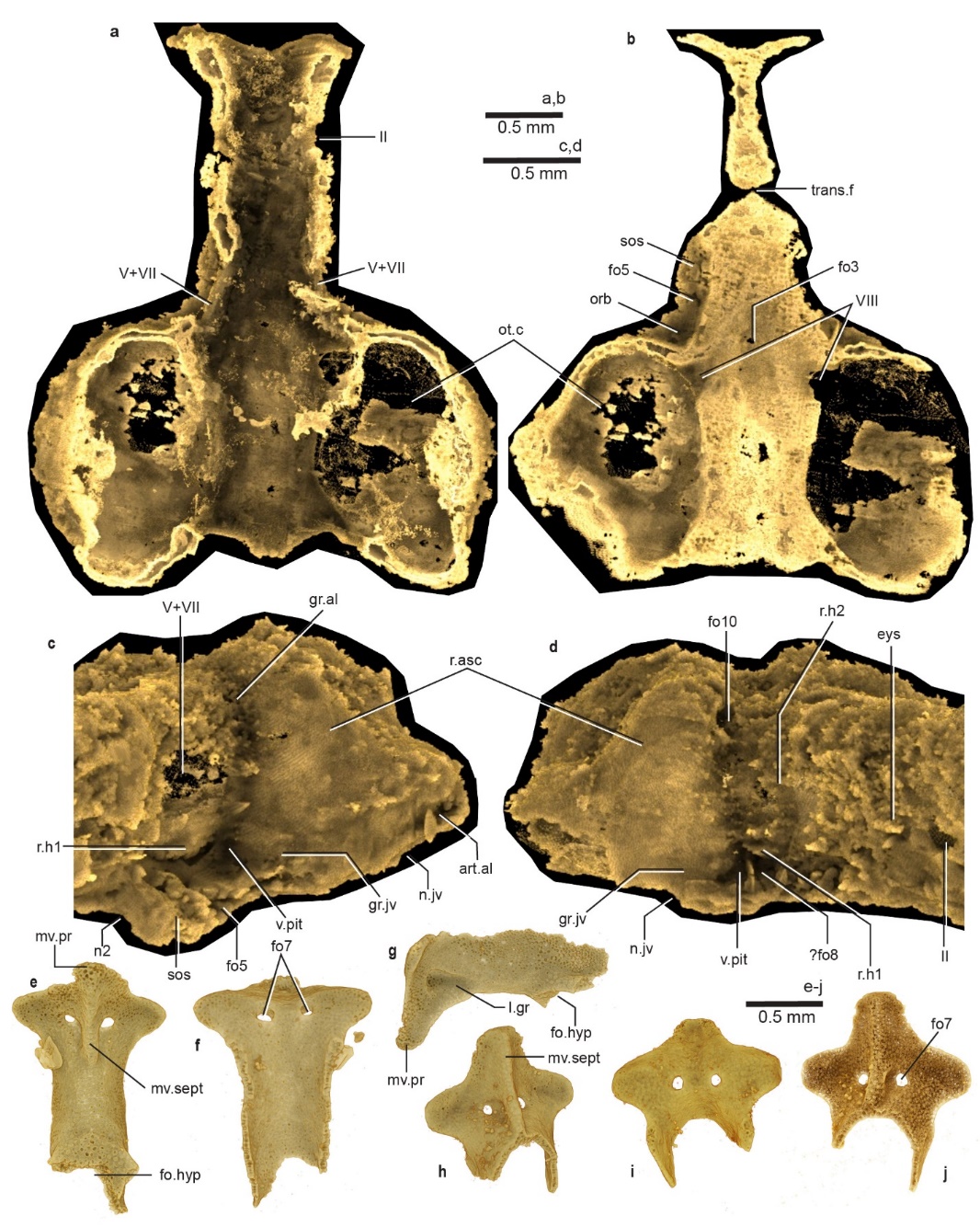


**SI Fig. 6. *Palaeospondylus australis* sp. nov. Holotype neurocranium and isolated ‘tauidion’ elements**. **a**, **b**, Horizontal slices through the holotype neurocranium at the level of the major cranial nerve foramina (**a**), and a deeper level near the neurocranial floor (**b**). **c**, **d**, Anterolateral views into the left (**c**) and right (**d**) orbital cavities of the holotype neurocranium. **e-g**, QMF52827.1, broken ‘tauidion’ in external, internal, and left lateral views. **h-j**, QMF52827.2, broken ‘tauidion’, off-centre right external view, internal view, and off-centre left external view at higher transparency. VIII, foramen for the auditory nerve; v.pit, foramen, possibly for pituitary vein. (For other abbreviations see SI Figs 2-5 captions.)

**
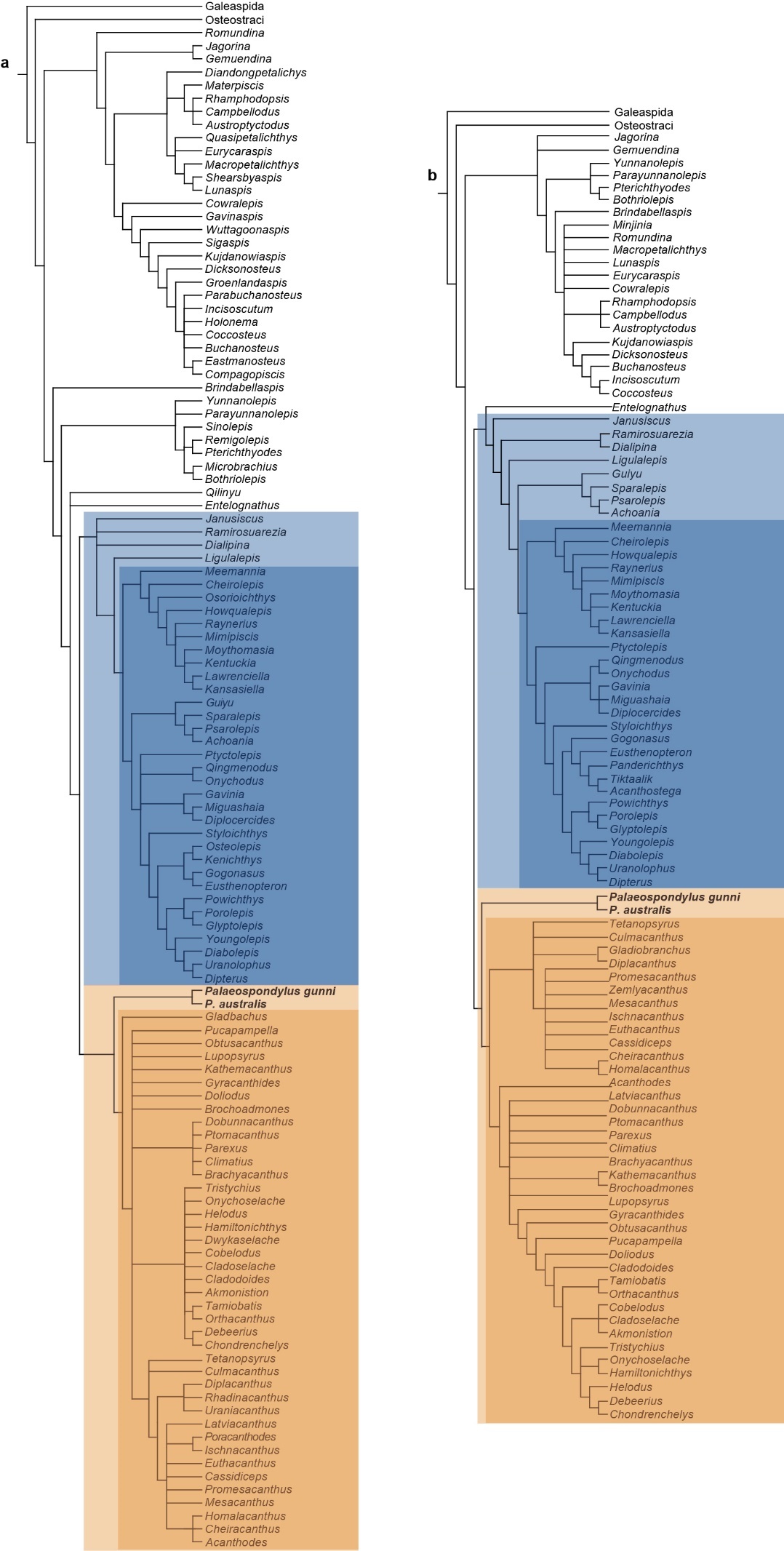
**

**SI Fig. 7. Strict consensus trees of early gnathostome relationship analyses showing *Palaeospondylus* as sister group to chondrichthyans. a**. Strict consensus of 302 shortest length trees with a length of 1143 steps (367 characters, ‘gammation’ as hyomandibula), or 313 trees (368 characters, ‘gammation’ as part of lateral commissure) in our analysis 1 based on the matrix revised from Lu et al. (2017), using the same taxa plus *P. gunni* and *P. australis*; both scenarios show *Palaeospondylus* at the base of the chondrichthyan total group. **b.** Strict consensus of 76 shortest length trees with a length of 876 steps based on the matrix of Brownstein (2023), using the same taxa plus *P. australis*. Equal weights parsimony analysis using the ratchet resulted in 82 trees with a length of 874 steps.


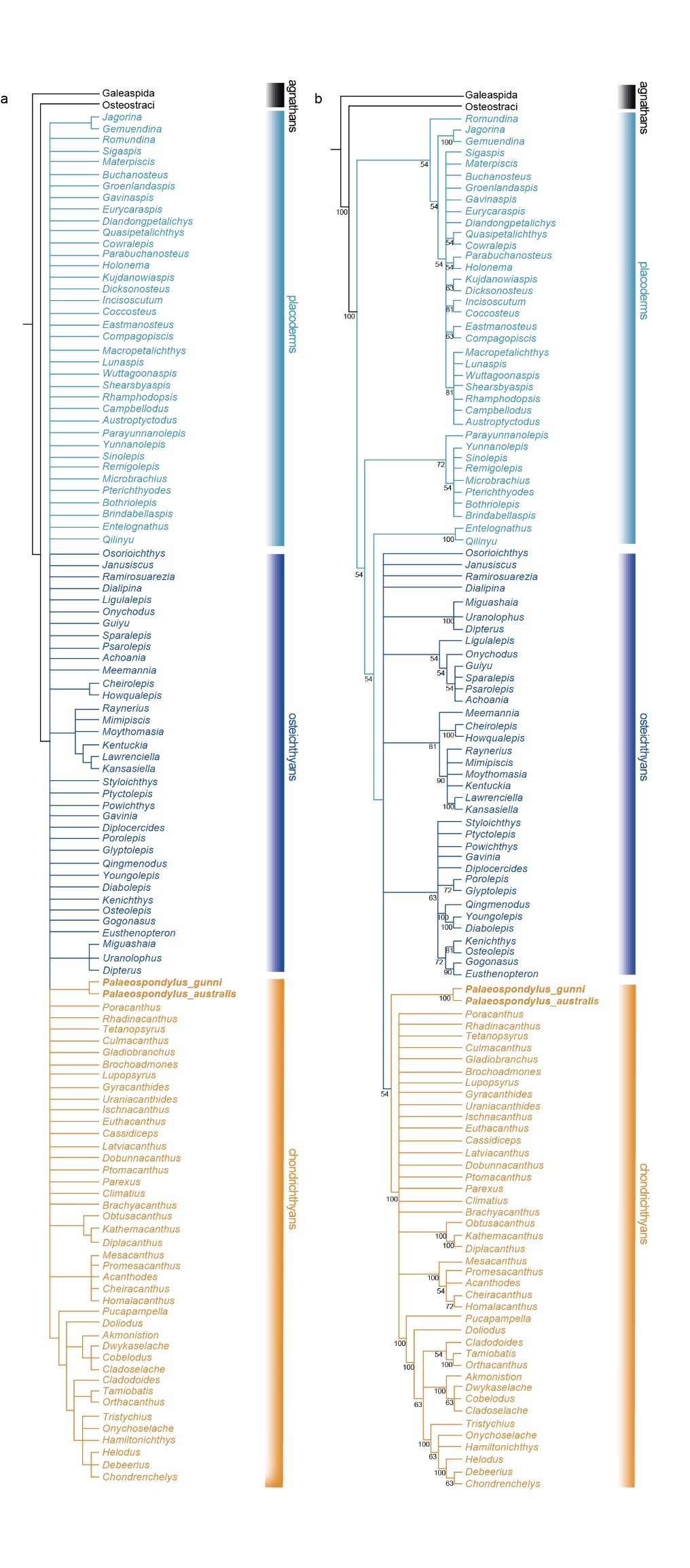


**SI Figure 8. Early gnathostome relationship analyses showing *Palaeospondylus* basal to chondrichthyans. a**. Strict consensus of 11 shortest length trees with a length of 376 steps based on the our matrix 4, revised from Lu *et al*. (2017), using the same taxa plus *P. gunni* and *P. australis*; **b.** 50% majority consensus trees.

**SI Tables.**

**SI Table 1**. *Palaeospondylus australis* elements from the Cravens Peak Beds.


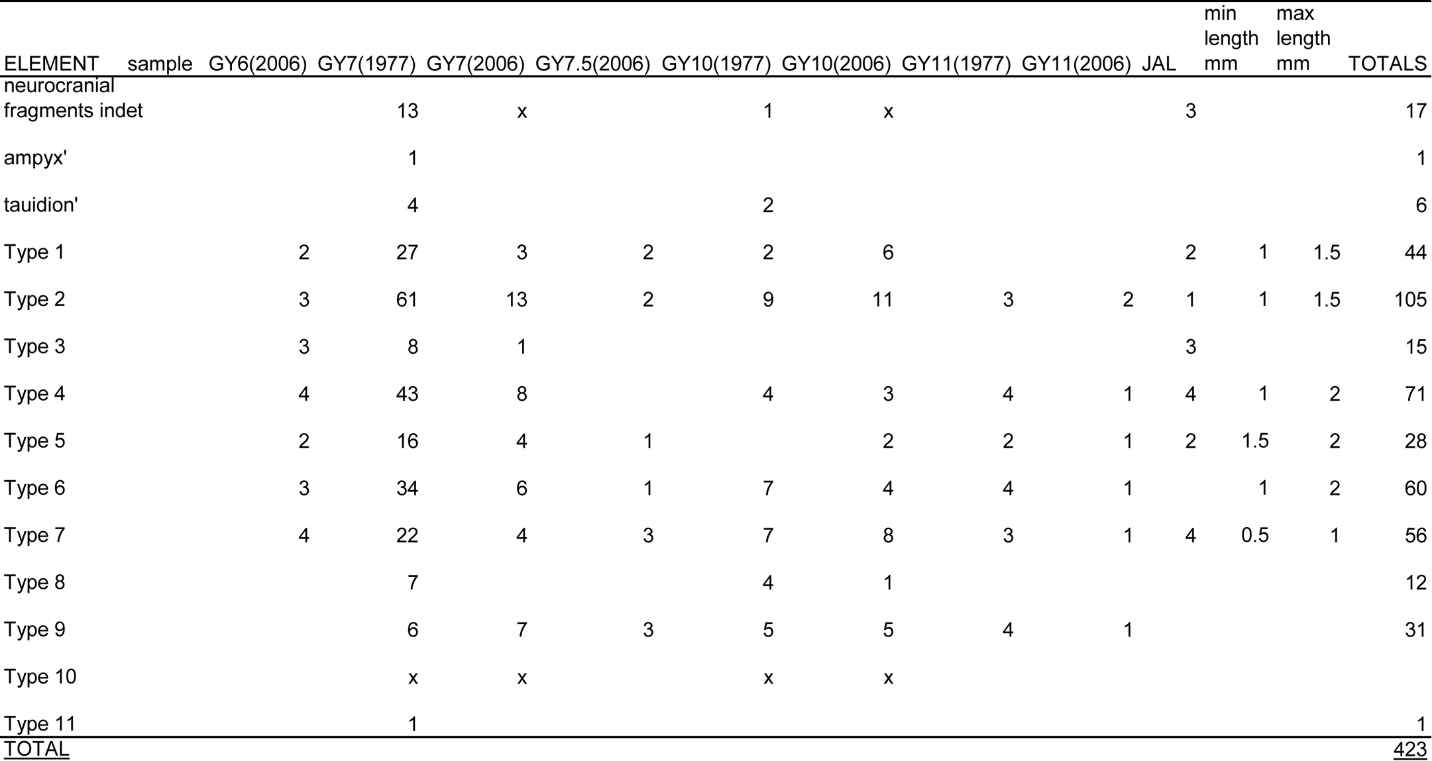


**SI Table 2**. Nomenclature used for identifying structures in *Palaeospondylus gunni* by previous authors and in this publication.


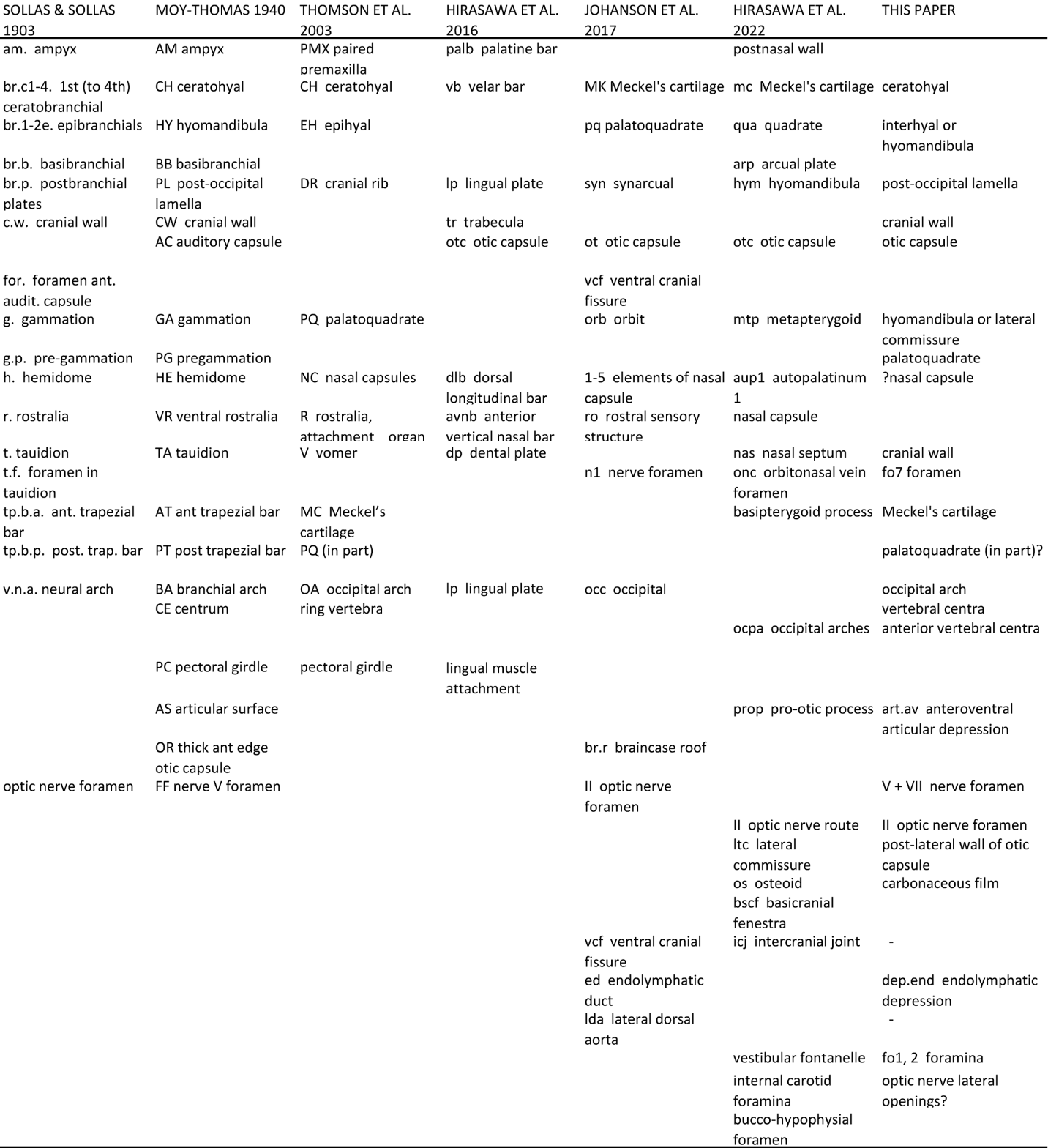


**VII. Phylogenetic Data**

**List of Taxa**

In our analyses 1, 2 and 4, the same taxa are used as in Lu *et al*. (2017), with the addition of *Palaeospondylus australis* and *P. gunni*. thus 96 taxa. Our third analysis based on the dataset used by Brownstein (2023) has 100 taxa (and 284 characters). Several taxon names have been updated in all matrices: *Gladiobranchus* to *Uraniacanthus probaton* (Newman *et al*. 2012), ‘*Ligulalepis*’ to *Ligulalepis* (Burrow *et al*. 2023), *Vernicomacanthus waynensis* to *Dobunnacanthus waynensis* (Dearden *et al*. 2021).

**List of Characters for Analyses 1 and 2, Based on the Lu et al. (2017) Dataset**

***Characters 281-332 are taken from Zhu et al. 2016. Characters 333-346 are new or reorganized characters based on the anatomical works in the current paper. Characters 345-354 are taken from Coates et al. 2018,*** a***nd character 355 is taken from Dearden et al. 2019. Characters 356-360 are from Zhu et al. 2019. Characters 361-368 are new.***

1. Tessellate prismatic calcified cartilage: 0. absent; 1. present.

2. Prismatic calcified cartilage: 0. single layered; 1. multi-layered.

***Note:*** *This character is contingent on c.1 (tesselate prismatic calcified cartilage) being coded as state 1.*

3. Perichondral bone: 0. present; 1. absent.

4. Extensive endochondral ossification: 0. absent; 1. present.

5. Enamel(oid) present on dermal bones and scales: 0. absent; 1. present.

6. Enamel: 0. single-layered; 1. multi-layered.

***Note:*** *This character is contingent on c. 5 (enamel(oid) on dermal bones and scales) being coded as state 1.*

7. Enamel layers: 0. applied directly to one another (ganoine); 1. separated by layers of dentine.

***Note:*** *This character is contingent on c. 5 (enamel(oid) on dermal bones and scales) being coded as state 1.*

8. Pore canal network (*sensu* Zhu et al., 2010): 0. absent; 1. present.

9. Dentinous tissue: 0. absent; 1. present.

10. Dentine kind: 0. mesodentine; 1. semidentine; 2. orthodentine.

***Note:*** *This character is contingent on c. 9 (dentinous tissue) being coded as state 1.*

11. Bone cell lacunae in body scale bases: 0. present; 1. absent.

12. Main dentinous tissue forming fin spine: 0. osteodentine; 1. orthodentine.

***Note:*** *This character is contingent on c. 9 (dentinous tissue) being coded as state 1.*

13. Longitudinal scale alignment in fin webs: 0. present; 1. absent.

14. Differentiated lepidotrichia: 0. absent; 1. present.

15. Body scale growth pattern: 0. comprising single odontode unit/generation ("monodontode"); 1. comprising a complex of multiple odontode generations/units ("polyodontode").

***Note:*** *This character is coded as inapplicable in taxa that lack scales.*

16. Body scale growth concentric: 0. absent; 1. present.

***Note:*** *This character is coded as inapplicable in taxa that lack scales.*

17. Generations of odontodes: 0. buried areally; 1. growing resorbed.

18. Body scales with peg-and-socket articulation: 0. absent; 1. present.

***Note:*** *This character is coded as inapplicable in taxa that lack scales.*

19. Scale peg: 0. broad; 1. narrow.

***Note:*** *This character is contingent on c. 18 (body scales with peg-and-socket articulation) being coded as state 1.*

20. Anterodorsal process on scale: 0. absent; 1. present.

21. Body scale profile: 0. distinct crown and base demarcated by a constriction ("neck"); 1. flattened.

22. Profile of scales with constriction between crown and base: 0. neck similar in width to crown; 1. Neck greatly constricted, resulting in anvil-like shape.

***Note:*** *This character is contingent on c. 21 (body scale profile) being coded as state 0.*

23. Body scales with bulging base: 0. absent; 1. present.

24. Body scales with flattened base: 0. present; 1. absent.

25. Basal pore in scales: 0. absent; 1. present.

26. Flank scale alignment: 0. vertical rows oblique rows or hexagonal; 1. rhombic packing; 2. disorganised.

27. Scute-like ridge scales (basal fulcra): 0. absent; 1. present.

28. Sensory line canal: 0. perforates scales; 1. passes between scales C-shaped scales.

29. Dermal ornamentation: 0. smooth; 1. parallel, vermiform ridges; 2. concentric ridges; 3. tuberculate.

30. Sensory line network: 0. preserved as open grooves (sulci) in dermal bones; 1. sensory lines pass through canals in dermal bones (open as pores).

31. Sensory canals/grooves: 0. contained within the thickness of dermal bones; 1. contained in prominent ridges on visceral surface of bone.

32. Jugal portion of infraorbital canal joins supramaxillary canal: 0. present; 1. absent.

33. Dermal skull roof: 0. includes large dermal plates; 1. consists of undifferentiated plates or tesserae.

***Note:*** *Taxa in which the dermal skull roof does not include large dermal plates are coded as inapplicable for contingent characters relating to this.*

34. Anterior pit line of dermal skull roof: 0. absent; 1. present.

***Note:*** *This character is contingent on c.33 (dermal skull roof) being coded as state 0.*

35. Tessera morphology: 0. large interlocking polygonal plates; 1. microsquamose, not larger than body squamation.

36. Cranial spines: 0. absent; 1. present, multicuspid; 2. present, monocuspid.

37. Extent of dermatocranial cover: 0. complete; 1. incomplete (limited to skull roof)

38. Openings for endolymphatic ducts in dermal skull roof: 0. present; 1. absent.

39. Endolymphatic duct relationship to median skull roof bone (i.e. nuchal plate): 0 within median bone; 1 on bones flanking the median bone (e.g. paranuchals)

40. Pineal opening perforation in dermal skull roof: 0. present; 1. absent.

41. Dermal plate associated with pineal eminence or foramen: 0. contributes to orbital margin; 1. plate bordered laterally by skull roofing bones.

42. Broad supraorbital vaults: 0. absent; 1. present.

43. Median commisure between supraorbital sensory lines: 0. absent; 1. present.

44. Dermal cranial joint at level of sphenoid-otic junction: 0. absent; 1. present.

45. Otic canal extends through postparietals: 0. absent; 1. present.

46. Number of bones of skull roof lateral to postparietals: 0. two; 1 one; 2 more than two.

47. Suture between paired skull roofing bones (centrals of placoderms postparietals of osteichthyans): 0. straight; 1. sinusoidal.

48. Medial processes of paranuchal wrapping posterolateral corners of nuchal plate: 0. absent; 1 present; 2. paranuchals precluded from nuchal by centrals; 3. no median posterior skull roof bone.

49. Paired pits on ventral surface of nuchal plate: 0. absent; 1. present.

50. Sclerotic ring: 0. absent; 1. present.

51. Consolidated cheek plates: 0. absent; 1. present.

52. Cheek plate: 0. undivided; 1. divided (i.e., squamosal and preopercular).

***Note:*** *This character is contingent on c.51 (consolidated cheek plates) being coded as state 1.*

53. Subsquamosals in taxa with divided cheek: 0. absent; 1. present.

***Note:*** *This character is contingent on c.52 (cheek plate) being coded as state 1.*

54. Preopercular shape: 0. rhombic; 1. bar-shaped.

55. Vertical canal associated with preopercular/suborbital canal: 0. absent; 1. present.

56. Enlarged postorbital tessera separate from orbital series: 0. absent; 1. present.

57. Extent of maxilla along cheek: 0. to posterior margin of cheek; 1. cheek bones exclude maxilla from posterior margin of cheek.

58. Dermal neck joint: 0 absent; 1 present.

59. Sensory line scales/plates on head: 0. unspecialized; 1. apposed growth; 2. paralleling canal; 3. semicylindrical C-shaped ring scales.

60. Bony hyoidean gill-cover series (branchiostegals): 0. absent; 1. present.

61. Branchiostegal plate series along ventral margin of lower jaw: 0. Absent; 1. present.

***Note:*** *This character is contingent on c.60 (bony hyoidean gill-cover series) being coded as state 1.*

62. Branchiostegal ossifications: 0. plate-like; 1. narrow and ribbon-like; 2. filamentous.

***Note:*** *This character is contingent on c.60 (bony hyoidean gill-cover series) being coded as state 1.*

63. Branchiostegal ossifications: 0. Ornamented; 1. unornamented.

***Note:*** *This character is contingent on c.60 (bony hyoidean gill-cover series) being coded as state 1.*

64. Imbricated branchiostegal ossifications: 0. absent; 1. present.

***Note:*** *This character is contingent on c.60 (bony hyoidean gill-cover series) being coded as state 1.*

65. Median gular: 0. absent; 1. present.

66. Lateral gular: 0. absent; 1. present.

67. Opercular (submarginal) ossification: 0. absent; 1. present.

68. Shape of opercular (submarginal) ossification: 0. broad plate that tapers towards its proximal end; 1. narrow, rod-shaped.

69. Size of lateral gular plates: 0. extending most of length of the lower jaw; 1. restricted to the anterior third of the jaw (no longer than the width of three or four branchiostegals).

***Note:*** *This character is contingent on c.66 (lateral gular) being coded as state 1.*

70. Gill arches: 0. largely restricted to region under braincase; 1. extend far posterior to braincase.

71. Basihyal: 0. absent; 1. present.

72. Interhyal: 0. absent; 1. present.

73. Hypohyal: 0. absent; 1. present.

74. Endoskeletal urohyal: 0. absent; 1. present.

75. Oral dermal tubercles borne on jaw cartilages or at margins of the mouth: 0. absent; 1. present.

76. Oral dermal tubercles patterned in organised rows (teeth): 0. absent; 1. present.

***Note:*** *This character is contingent on c.75 (oral dermal tubercles borne on jaw cartilages or at margins of the mouth) being coded as state 1.*

77. Enamel(oid) on teeth: 0. absent; 1. present.

***Note:*** *This character is contingent on c.75 (oral dermal tubercles borne on jaw cartilages or at margins of the mouth) being coded as state 1 and c.76 (oral dermal tubercles patterned in organised rows) being coded as state 1.*

78. Cap of enameloid restricted to upper part of teeth (acrodin): 0. absent; 1. present.

***Note:*** *This character is contingent on c.75 (oral dermal tubercles borne on jaw cartilages or at margins of the mouth) being coded as state 1 and c.76 (oral dermal tubercles patterned in organised rows) being coded as state 1.*

79. Tooth whorls: 0. absent; 1. present.

***Note:*** *This character is contingent on c.75 (oral dermal tubercles borne on jaw cartilages or at margins of the mouth) being coded as state 1 and c.76 (oral dermal tubercles patterned in organised rows) being coded as state 1.*

80. Bases of tooth whorls: 0. single, continuous plate; 1. some or all whorls consist of separate tooth units.

***Note:*** *This character is contingent on c.79 (tooth whorls) being coded as state 1.*

81. Distribution of tooth whorls: 0. entire length of tooth row; 1. restricted to symphysial region.

***Note:*** *This character is contingent on c.79 (tooth whorls) being coded as state 1.*

82. Distribution of tooth whorls: 0. upper and lower jaws; 1. lower jaws only; 2. upper jaws only.

***Note:*** *This character is contingent on c.79 (tooth whorls) being coded as state 1.*

83. Teeth ankylosed to dermal bones: 0. absent; 1. present.

***Note:*** *This character is contingent on c.75 (oral dermal tubercles borne on jaw cartilages or at margins of the mouth) being coded as state 1 and c.85 (dermal jaw plates on biting surfaces of jaw cartilages) being coded as state 1.*

84. Plicidentine: 0. absent; 1. present.

***Note:*** *This character is contingent on c.75 (oral dermal tubercles borne on jaw cartilages or at margins of the mouth) being coded as state 1 and c.76 (oral dermal tubercles patterned in organised rows) being coded as state 1.*

85. Dermal jaw plates on biting surface of jaw cartilages: 0. absent; 1. present.

86. Premaxilla: 0. extends under orbit; 1. restricted anterior to orbit.

***Note:*** *This character is contingent on c.85 (dermal jaw plates on biting surfaces of jaw cartilages) being coded as state 1.*

87. Maxilla shape: 0. splint-shaped; 1. cleaver-shaped.

***Note:*** *This character is contingent on c.85 (dermal jaw plates on biting surfaces of jaw cartilages) being coded as state 1.*

88. Pair of tooth plates (anterior supragnathals or vomers) on ethmoidal plate: 0. absent; 1. present.

***Note:*** *This character is contingent on c.85 (dermal jaw plates on biting surfaces of jaw cartilages) being coded as state 1.*

89. Strong posterior flexion of dentary symphysis: 0. absent; 1. present.

***Note:*** *This character is contingent on c.85 (dermal jaw plates on biting surfaces of jaw cartilages) being coded as state 1.*

90. Extent of infradentaries: 0. along much of ventral margin of dentary; 1. restricted to posterior half of dentary.

***Note:*** *This character is contingent on c.85 (dermal jaw plates on biting surfaces of jaw cartilages) being coded as state 1.*

91. Coronoid fangs: 0. absent; 1. present.

***Note:*** *This character is contingent on c.85 (dermal jaw plates on biting surfaces of jaw cartilages) being coded as state 1.*

92. Position of upper mandibular arch cartilage (and associated cheek plate where present): 0. entirely suborbital; 1. with a postorbital extension.

93. Position of mandibular arch articulations: 0. terminal; 1. subterminal.

94. Autopalatine and quadrate: 0. comineralized; 1. separate mineralizations.

95. Large otic process of the palatoquadrate: 0. absent; 1. present.

96. Insertion area for jaw adductor muscles on palatoquadrate: 0. ventral or medial; 1. lateral.

97. Palatoquadrate relationship to dermal cheek bones: 0. articulation narrow and restricted; 1. broad articulation.

***Note:*** *This character is coded as inapplicable in taxa that lack dermal check bones.*

98. Palatoquadrate fused with neurocranium: 0. absent; 1. present.

99. Oblique ridge or groove along medial face of palatoquadrate: 0. Absent; 1. present.

100. Fenestration of palatoquadrate at basipterygoid articulation: 0. absent; 1. present.

101. Perforate or fenestrate anterodorsal (metapterygoid) portion of palatoquadrate: 0. absent; 1. present.

102. Pronounced dorsal process on Meckelian bone or cartilage: 0. absent; 1. present.

103. Number of coronoids: 0. four or more; 1. three or fewer.

***Note:*** *This character is contingent on c.85 (dermal jaw plates on biting surfaces of jaw cartilages) being coded as state 1.*

104. Preglenoid process: 0. absent; 1. present.

105. Jaw articulation located on rearmost extremity of mandible: 0. absent; 1. present.

106. Precerebral fontanelle: 0. absent; 1. present.

107. Median dermal bone of palate (parasphenoid): 0. absent; 1. present.

108. Parasphenoid: 0. lozenge-shaped; 1. splint-shaped; 2. diamond-shaped.

***Note:*** *This character is contingent on c.107 (median dermal bone of palate) being coded as state 1.*

109. Multifid anterior margin of parasphenoid denticle plate: 0. absent; 1. present.

***Note:*** *This character is contingent on c.107 (median dermal bone of palate) being coded as state 1.*

110. Enlarged ascending processes of parasphenoid: 0. absent; 1. present.

***Note:*** *This character is contingent on c.107 (median dermal bone of palate) being coded as state 1.*

111. Buccohypophysial canal in parasphenoid: 0. single; 1. paired.

***Note:*** *This character is contingent on c.107 (median dermal bone of palate) being coded as state 1.*

112. Nasal opening(s): 0. dorsal, placed between orbits; 1. ventral and anterior to orbit.

113. External opening of posterior nostril and orbit: 0. separated by dermal bone(s); 1. confluent.

114. Olfactory tracts: 0. short, with olfactory capsules situated close to telencephalon cavity; 1. elongate and tubular (much longer than wide).

115. Prominent pre-orbital rostral expansion of the neurocranium: 0. present; 1. absent

116. Pronounced sub-ethmoidal keel: 0. absent; 1. present.

117. Internasal vacuities: 0. absent; 1. present.

118. Discrete division of the ethmoid and more posterior braincase at the level of the optic tract canal: 0. absent; 1. present.

119. Position of myodome for superior oblique eye muscles: 0. posterior and dorsal to foramen for nerve II; 1. anterior and dorsal to foramen.

120. Endoskeletal intracranial joint: 0. absent; 1. present.

121. Spiracular groove on basicranial surface: 0. absent; 1. present.

122. Transverse otic process: 0. present; 1. absent.

123. Jugular canal: 0. long (invested in otic region along length of skeletal labyrinth); 1. short (restricted to short portion of region of skeletal labyrinth, or anterior to it); 2. absent (jugular vein uninvested in otic region).

124. Spiracular groove on lateral commissure: 0. absent; 1. present.

***Note:*** *This character is coded as inapplicable in taxa that lack a lateral commissure (postorbital process sensu Giles et al. 2015).*

125. Subpituitary fenestra: 0. absent; 1. present.

126. Supraorbital shelf broad with convex lateral margin: 0. absent; 1. present.

127. Orbit dorsal or facing dorsolaterally, surrounded laterally by endocranium: 0. present; 1. absent.

128. Eyestalk attachment area: 0. absent; 1. present.

129. Postorbital process: 0. absent; 1. present.

130. Canal for jugular in postorbital process: 0. absent; 1. present.

***Note:*** *This character is contingent on c.129 (postorbital process) being coded as state 1.*

131. Series of perforations for innervation of supraorbital sensory canal in supraorbital shelf: 0. absent; 1. present.

132. Extended prehypophysial portion of sphenoid: 0. absent; 1. present.

133. Narrow interorbital septum, with outer walls in contact along midline forming a single sheet: 0. absent; 1. present.

134. The main trunk of facial nerve (N. VII): 0. elongate and passes anterolaterally through orbital floor; 1. stout, divides within otic capsule at the level of the transverse otic wall.

135. Course of hyoid ramus of facial nerve (N. VII) relative to jugular canal: 0. traverses jugular canal, with separate exit in otic region; 1. intersects jugular canal, with exit through posterior jugular foramen.

***Note:*** *This character is coded as inapplicable in taxa where the jugular canal is uninvested in the otic region.*

136. Glossopharyngeal nerve (N. IX) exit: 0. foramen situated posteroventral to otic capsule and anterior to metotic fissure; 1. through metotic fissure.

***Note:*** *This character is coded as inapplicable in taxa that lack a metotic fissure.*

137. Relationship of cranial endocavity to basisphenoid: 0. endocavity occupies full depth of sphenoid; 1. endocavity dorsally restricted.

138. Subcranial ridges: 0. absent; 1. present.

139. Ascending basisphenoid pillar pierced by common internal carotid: 0. absent; 1. present.

140. Canal for lateral dorsal aorta within basicranial cartilage: 0. absent; 1. present.

141. Entrance of internal carotids: 0. through separate openings flanking the hypophyseal opening or recess; 1. through a common opening at the central midline of the basicranium.

142. Canal for efferent pseudobranchial artery within basicranial cartilage: 0. absent; 1. present.

143. Position of basal/basipterygoid articulation: 0. same anteroposterior level as hypophysial opening; 1. anterior to hypophysial opening; 2. posterior to hypophysial opening.

144. Articulation between neurocanium and palatoquadrate posterodorsal to orbit (suprapterygoid articulation): 0 absent; 1. present.

145. Labyrinth cavity: 0. separated from the main neurocranial cavity by a cartilaginous or ossified capsular wall; 1. skeletal capsular wall absent.

146. Basipterygoid process (basal articulation) with vertically oriented component: 0. absent; 1. present.

147. Pituitary vein canal: 0. dorsal to level of basipterygoid process; 1. flanked posteriorly by basipterygoid process.

148. External (horizontal) semicircular canal: 0. absent; 1. present.

149. External (horizontal) semicircular canal: 0. joins the vestibular region dorsal to posterior ampulla; 1. joins level with posterior ampulla.

150. Horizontal semicircular canal in dorsal view: 0. medial to path of jugular vein; 1. dorsal to jugular vein.

151. Lateral cranial canal: 0. absent; 1. present.

152. Posterior dorsal fontanelle: 0. absent; 1. present.

153. Shape of posterior dorsal fontanelle: 0. approximately as long as broad; 1. much longer than wide, slot-shaped.

***Note:*** *This character is contingent on c.153 (posterior dorsal fontanelle) being coded as state 1.*

154. Synotic tectum: 0 absent; 1 present.

155. Dorsal ridge: 0 absent; 1 present.

156. Shape of median dorsal ridge anterior to endolymphatic fossa: 0 developed as a squared-off ridge or otherwise ungrooved; 1 bears a midline groove.

***Note:*** *This character is contingent on c.155 (dorsal ridge) being coded as state 1.*

157. Position of hyomandibula articulation on neurocranium: 0. below or anterior to orbit, on ventrolateral angle of braincase; 1. on otic capsule, posterior to orbit.

158. Position of hyomandibula articulation relative to structure of skeletal labyrinth: 0. anterior or lateral to skeletal labyrinth; 1. at level of posterior semicircular canal.

***Note:*** *This character is coded as inapplicable in taxa where the hyomandibula articulates level with the orbit.*

159. Hyoid arch articulation on braincase: 0. single; 1. double.

160. Branchial ridges: 0. present; 1. reduced to vagal process; 2. absent (articulation made with bare cranial wall).

161. Craniospinal process: 0. absent; 1. present.

162. Ventral cranial fissure: 0. absent; 1. present.

163. Basicranial fenestra: 0. absent; 1. present.

164. Metotic (otic-occipital) fissure: 0. absent; 1. present.

165. Vestibular fontanelle: 0. absent; 1. present.

166. Occipital arch wedged in between otic capsules: 0. absent; 1. present.

167. Spino-occipital nerve foramina: 0. two or more, aligned horizontally; 1. one or two, dorsoventrally offset.

168. Ventral notch between parachordals: 0. present or entirely unfused; 1. absent.

169. Parachordal shape: 0. forming a broad, flat surface as wide as the otic capsules; 1. mediolaterally constricted relative to the otic capsules.

170. Stalk-shaped parachordal/occipital region: 0. absent; 1. present.

171. Paired occipital facets: 0. absent; 1. present.

172. Size of aperture to notochordal canal: 0. much smaller than foramen magnum; 1. as large, or larger, than foramen magnum.

173. Canal for median dorsal aorta within basicranium: 0. absent; 1. present.

174. Hypotic lamina (and dorsally directed glossopharyngeal canal): 0. absent; 1. present

175. Macromeric dermal shoulder girdle: 0. present; 1. absent.

176. Dermal shoulder girdle composition: 0. ventral and dorsal (scapular) components; 1. ventral component only.

***Note:*** *This character is contingent on c.175 (macromeric dermal shoulder girdle) being coded as state 0.*

177. Shape of dorsal blade of dermal shoulder girdle (either cleithrum or anterolateral plate): 0. spatulate; 1. pointed.

***Note:*** *This character is contingent on c.176 (dermal shoulder girdle composition) being coded as state 0.*

178. Dermal shoulder girdle forming a complete ring around the trunk: 0. present; 1. absent.

***Note:*** *This character is contingent on c.176 (dermal shoulder girdle composition) being coded as state 0.*

179. Pectoral fenestra completely encircled by dermal shoulder armour: 0. present; 1. absent.

***Note:*** *This character is contingent on c.176 (dermal shoulder girdle composition) being coded as state 0.*

180. Median dorsal plate: 0. absent; 1. present.

***Note:*** *This character is contingent on c.176 (dermal shoulder girdle composition) being coded as state 0.*

181. Posterior dorsolateral (PDL) plate or equivalent: 0. absent; 1. present.

***Note:*** *This character is contingent on c.176 (dermal shoulder girdle composition) being coded as state 0.*

182. Pronounced internal median keel on dorsal shoulder girdle (i.e., crista of median dorsal plate): 0. absent; 1. present.

***Note:*** *This character is contingent on c.176 (dermal shoulder girdle composition) being coded as state 0.*

183. Crista internalis of dermal shoulder girdle: 0. absent; 1. present.

***Note:*** *This character is contingent on c.176 (dermal shoulder girdle composition) being coded as state 0.*

184. Scapular infundibulum: 0. absent; 1. present.

***Note:*** *This character is contingent on c.176 (dermal shoulder girdle composition) being coded as state 0.*

185. Scapular process of shoulder endoskeleton: 0. absent; 1. present.

186. Ventral margin of separate scapular ossification: 0. horizontal; 1. deeply angled

187. Cross sectional shape of scapular process: 0. flattened or strongly ovate; 1. subcircular.

188. Flange on trailing edge of scapulocoracoid: 0. absent; 1. present.

189. Scapular process with posterodorsal angle: 0. absent; 1. present.

190. Endoskeletal postbranchial lamina on scapular process: 0. present; 1. absent.

191. Mineralisation of internal surface of scapular blade: 0. mineralised all around; 1. unmineralised on internal face forming a hemicylindrical cross-section.

192. Coracoid process: 0. absent; 1. present.

193. Procoracoid mineralisation: 0. absent; 1. present.

194. Fin base articulation on scapulocoracoid: 0. deeper than wide (stenobasal); 1. wider than deep (eurybasal).

195. Pectoral fin articulation: 0. monobasal; 1. polybasal.

196. Number of basals in polybasal pectoral fins: 0. three or more; 1. two.

197. Branching radials in paired fins: 0. absent; 1. present.

198. Number of mesomeres in metapterygial axis: 0. five or fewer; 1. seven or more.

199. Biserial pectoral fin endoskeleton: 0. absent; 1. present.

200. Perforate propterygium: 0. absent; 1. present.

201. Filamentous extension of pectoral fin from axillary region: 0. absent; 1. present.

202. Pelvic fins: 0. absent; 1. present.

203. Pelvic claspers: 0. absent; 1. present.

204. Dermal pelvic clasper ossifications: 0. absent; 1. present.

***Note:*** *This character is contingent on c.203 (pelvic claspers) being coded as state 1.*

205. Pectoral fins covered in macromeric dermal armour: 0. absent; 1. present.

206. Pectoral fin base has large, hemispherical dermal component: 0. absent; 1. present

207. Dorsal fin spines: 0. absent; 1. present.

208. Anal fin spine: 0. absent; 1. present.

209. Paired fin spines: 0. absent; 1. present.

210. Median fin spine insertion: 0. shallow, not greatly deeper than dermal bones/scales; 1. deep.

***Note:*** *This character is coded as inapplicable in taxa that lack median fin spines.*

211. Intermediate fin spines: 0. absent; 1. present.

212. Fin spine cross-section: 0. round or horseshoe shaped; 1. flat-sided, with rectangular profile.

***Note:*** *This character is coded as inapplicable in taxa that lack fin spines.*

213. Intermediate spines when present: 0. one pair; 1. multiple pairs.

***Note:*** *This character is coded as inapplicable in taxa that intermediate fin spines.*

214. Prepectoral fin spines: 0. absent; 1. present.

215. Fin spines with ridges: 0. absent; 1. present.

***Note:*** *This character is coded as inapplicable in taxa that lack fin spines.*

216. Fin spines with nodes: 0. absent; 1. present.

***Note:*** *This character is coded as inapplicable in taxa that lack fin spines.*

217. Fin spines with rows of large retrorse denticles: 0. absent; 1. present.

***Note:*** *This character is coded as inapplicable in taxa that lack fin spines.*

218. Expanded spine rib on leading edge of spine: 0. absent; 1. present.

***Note:*** *This character is coded as inapplicable in taxa that lack fin spines.*

219. Spine ridges: 0. converging at the distal apex of the spine; 1. converging on leading edge of spine.

***Note:*** *This character is coded as inapplicable in taxa that lack fin spines.*

220. Synarcual: 0. absent; 1. present.

221. Series of thoracic supraneurals: 0. absent; 1. present.

222. Number of dorsal fins, if present: 0. one; 1. two.

***Note:*** *This character is coded as inapplicable in taxa that lack dorsal fins.*

223. Posterior dorsal fin shape: 0. base approximately as broad as tall, not broader than all of other median fins; 1. base much longer than the height of the fin, substantially longer than any of the other dorsal fins.

***Note:*** *This character is contingent on c.222 (number of dorsal fins) being coded as state 1 and this character is coded as inapplicable in taxa that lack dorsal fins.*

224. Basal plate in dorsal fin: 0. absent; 1. present.

***Note:*** *This character is coded as inapplicable in taxa that lack dorsal fins.*

225. Branching radial structure articulating with dorsal fin basal plate: 0. absent; 1. present.

***Note:*** *This character is coded as inapplicable in taxa that lack dorsal fins.*

226. Anal fin: 0. absent; 1. present.

227. Basal plate in anal fin: 0. absent; 1. present.

***Note:*** *This character is coded as inapplicable in taxa that lack anal fins.*

228. Caudal radials: 0. extend beyond level of body wall and deep into hypochordal lobe; 1. radials restricted to axial lobe.

229. Supraneurals in axial lobe of caudal fin: 0. absent; 1. present.

230. Epichordal lepidotrichia in caudal fin: 0. absent; 1. present.

***Note:*** *This character is coded as inapplicable in taxa that lack lepidotrichia.*

231. Enamel and pore canals: 0. enamel absent from inner surface of pores; 1. enamel lines portions of pore canal.

***Note:*** *This character is contingent on c. 231 (pore canal network) being coded as state 1.*

232. Canal-bearing bone of skull roof extends far past posterior margin of parietals: 0. no; 1. yes.

233. Pineal eminence (in taxa lacking pineal foramen): 0. absent; 1. present.

234. Position of anterior pitline: 0. on postparietal; 1. on parietal.

235. Opening in dermal skull roof for spiracular bounded by bones carrying otic canal: 0. absent; 1. present.

236. Median skull roof bone between postparietals: 0. absent; 1. present.

237. Westoll lines: 0. absent; 1. present.

238. Preoperculosubmandibular: 0. absent; 1. present.

239. Hyomandibula: 0. imperforate; 1. perforate.

240. Urohyal shape: 0. absent; 1. vertical plate.

241. Maxilla (in taxa with marginal jaw bones): 0. present; 1. absent.

***Note:*** *This character is contingent on c.85 (dermal jaw plates on biting surfaces of jaw cartilages) being coded as state 1.*

242. Length of dentary: 0. constitutes a majority of jaw length; 1. half the length of jaw or less.

***Note:*** *This character is contingent on c.85 (dermal jaw plates on biting surfaces of jaw cartilages) being coded as state 1.*

243. Labial pit: 0. absent; 1. present.

***Note:*** *This character is contingent on c.85 (dermal jaw plates on biting surfaces of jaw cartilages) being coded as state 1.*

244. Prearticular symphysis: 0. absent; 1. present.

***Note:*** *This character is contingent on c.85 (dermal jaw plates on biting surfaces of jaw cartilages) being coded as state 1.*

245. Mandibular sensory canal: 0. extends through infradentaries; 1. extends through infradentaries and dentary.

***Note:*** *This character is contingent on c.85 (dermal jaw plates on biting surfaces of jaw cartilages) being coded as state 1.*

246. Extensive flange composed of prearticular and Meckelian bone that extends beyond ventral edge of outer dermal series: 0. absent; 1. present.

***Note:*** *This character is contingent on c.85 (dermal jaw plates on biting surfaces of jaw cartilages) being coded as state 1.*

247. Posterior coronoid: 0. similar to anterior coronoids; 1. forms expanded coronoid process.

***Note:*** *This character is contingent on c.85 (dermal jaw plates on biting surfaces of jaw cartilages) being coded as state 1.*

248. Retroarticular process: 0. absent; 1. present.

249. Inturned medial process of premaxilla: 0. absent; 1. present.

***Note:*** *This character is contingent on c.85 (dermal jaw plates on biting surfaces of jaw cartilages) being coded as state 1.*

250. Anteriorly directed adductor fossae between neurocranium and skull roof: 0. absent; 1. present.

251. Vomerine fangs: 0. absent; 1. present.

***Note:*** *This character is contingent on c.85 (dermal jaw plates on biting surfaces of jaw cartilages) being coded as state 1.*

252. Number of dermopalatines: 0. multiple; 1. one.

***Note:*** *This character is contingent on c.85 (dermal jaw plates on biting surfaces of jaw cartilages) being coded as state 1.*

253. Entopterygoids: 0. separated; 1. contact along midline.

***Note:*** *This character is contingent on c.85 (dermal jaw plates on biting surfaces of jaw cartilages) being coded as state 1.*

254. Rostral tubuli: 0. absent; 1. present.

255. Position of anterior nostril: 0. facial; 1. at oral margin.

256. Posterior nostril: 0. facial; 1. at margin of oral cavity; 2 palatal.

257. Three large pores (in addition to nostrils) associated with each side of ethmoid: 0. absent; 1. present.

258. Ventral face of nasal capsule in taxa with mineralized ethmoid: 0. complete; 1. fenestra ventrolateralis; 2. entire floor unmineralized.

259. Size of profundus canal in postnasal wall: 0. small; 1. large.

260. Paired pineal and parapineal tracts: 0. absent; 1 present.

261. Posterior of parasphenoid: 0. restricted to ethmosphenoid region; 1. extends to otic region.

***Note:*** *This character is contingent on c.107 (median dermal bone of palate) being coded as state 1.*

262. Endoskeletal spiracular canal: 0. open; 1. spiracular bar; 2. complete enclosure in canal.

263. Barbed lepidotrichial segments: 0. absent; 1. present.

264. Relative position of jugular groove/canal and hyomandibular articulation: 0. hyomandibula dorsal; 1. hyomandibula ventral; 2. hyomandibula straddles.

265. Optic lobes: 0. narrower than cerebellum; 1. same width or wider than cerebellum.

266. Hypophyseal chamber: 0. projects posteroventrally; 1. projects ventrally or anteroventrally.

267. Crus commune of anterior and posterior semicircular canals: 0. dorsal to endocranial roof; 1. ventral to endocranial roof.

268. Horizontal semicircular canal: 0. obiquely oriented; 1. horizontally oriented.

269. Supraotic cavity: 0 absent; 1 present

270. Pelvic girdle with substantial dermal component: 0. present; 1. absent.

271. Pelvic fin spine: 0. absent; 1. present.

272. Pelvic fin: 0. monobasal; 1. polybasal.

273. Postparietals/centrals: 0. absent; 1. present.

274. Condition of postparietals/centrals: 0. do not meet in midline; 1. meet in midline; 2. single midline bone.

275. Parietals: 0. absent; 1. present.

276. Condition of parietals: 0. do not meet in midline; 1. meet in midline.

277. Endoskeletal lamina (postnasal wall) separating posterior nostril and orbit: 0. absent; 1. present.

278. Pituitary vein canal: 0. discontinuous, enters the cranial cavity; 1. discontinuous, enters hypophysial recess; 2. continuous transverse vein.

279. Crus commune: 0. absent; 1. present.

***Note:*** *A crus commune in the skeletal labyrinth is judged to be absent if the saccular cavity separates the anterior and posterior semicircular canals.*

***The following characters are taken from Zhu et al. 2016***

280. Opercular cover of branchial chamber: 0. complete or partial; 1. separate gill covers and gill slits. (Zhu et al. 2016, Character 31)

281. Lateral plate: 0. absent; 1. present. (Zhu et al. 2016, Character 165)

282. Location of pineal foramen/eminence: 0. level with posterior margin of orbits; 1. well posterior of orbits. (Zhu et al. 2016, Character 166)

283 Parietals (preorbitals of placoderms) surround pineal foramen or eminence: 0. yes; 1. no. (Zhu et al. 2016, Character 167)

284. Paranuchal number: 0. one pair; 1. two pairs. ((Zhu et al. 2016, Character 169)

285. Large unpaired median bone contributing to posterior margin of skull roof: 0. absent; 1. present. (Zhu et al. 2016, Character 170)

286. Contact of nuchal or centronuchal plate with paired preorbital plates: 0. absent; 1. present. (Zhu et al. 2016, Character 171)

287. Junction of posterior pitline and main lateral line: 0. far in front of posterior margin of skull roof; 1. close to posterior margin of skull roof. (Zhu et al. 2016, Character 173)

288. Premaxilla forming part of orbit: 0. absent; 1. present. (Zhu et al. 2016, Character 183)

289. Course of supraorbital canal: 0. straight; 1. Lyre-shaped. (Zhu et al. 2016, Character 191)

290. Posterior end of supraorbital canal: 0 in postparietal; 1. in parietal; 2. in intertemporal; 3. in nuchal plate; 4. in postpineal plate. (Zhu et al. 2016, Character 192)

291. Contact between otic and supraorbital canals: 0. not in contact; 1. in contact. (Zhu et al. 2016, Character 193)

292. Infradentary: 0. absent; 1. present. (Zhu et al. 2016, Character 204)

293. Opercular suspension on braincase: 0. absent; 1. present. (Zhu et al. 2016, Character 222)

294. Presupracleithrum: 0. absent; 1. present. (Zhu et al. 2016, Character 229)

295. Anocleithrum: 0. element developed as postcleithrum; 1. element developed as anocleithrum sensu stricto. (Zhu et al. 2016, Character 230)

296. Dorsal cleithrum (AL of the Placodermi), ventral cleithrum (AVL of the Placodermi): 0. unfused; 1. Fused. (Zhu et al. 2016, Character 232)

297. Relationship of clavicle to cleithrum: 0. ascending process of clavicle overlapping cleithrum laterally; 1. ascending process of clavicle wrapping round anterior edge of cleithrum, overlapping it both laterally and mesially. (Zhu et al. 2016, Character 233)

298. Triradiate scapulocoracoid: 0. absent; 1. present. (Zhu et al. 2016, Character 234)

299. Subscapular foramen: 0. absent; 1. present. (Zhu et al. 2016, Character 235)

300. Pectoral propterygium: 0. absent; 1. present. (Zhu et al. 2016, Character 236)

301. Ethmoid articulation for palatoquadrate: 0. placed on postnasal wall; 1. extends posteriorly to the level of N.II. (Zhu et al. 2016, Character 240)

302. Contact between palatoquadrate and dermal cheek bones: 0. continuous contact of metapterygoid and autopalatine; 1. metapterygoid and autopalatine contacts separated by gap between commissural lamina of palatoquadrate and cheek bones. (Zhu et al. 2016, Character 241)

303. Metapterygoid with developed medial ventral protrusion: 0. absent; 1. present. (Zhu et al. 2016, Character 242)

304. Central dermal skull bone (nuchal) with converging posterior pit-line canals and supraorbital canal: 0. absent; 1. converging but not meeting; 2. crossing as an X in bone. (Zhu et al. 2016, Character 246)

305. Deep, high supragnathal bone with durophagous occlusal surface: 0. absent; 1. present. (Zhu et al. 2016, Character 247)

306. Cuccullaris fossa (trapezius fossa): 0. open posteriorly; 1. constrained posteriorly. (Zhu et al. 2016, Character 324)

307. Foremost contact between palatoquadrate and braincase: 0. anterior to nasal cavity; 1. posterior to nasal cavity. (Zhu et al. 2016, Character 326)

308. Laterally extending palatoquadrate: 0. absent; 1. present. (Zhu et al. 2016, Character 327)

309. Close association of pineal organ and nasal cavitie: 0. absent; 1. present. (Zhu et al. 2016, Character 328)

310. Postnasal plate 0 absent: 0. absent; 1. present. (Zhu et al. 2016, Character 341)

311. Preorbital recess of premedian plate: 0. absent; 1. present. (Zhu et al. 2016, Character 342)

312. Nuchal reaching or almost reaching orbital margin: 0. absent; 1. present. (Zhu et al. 2016, Character 345)

313. Postmarginal plate: 0. absent; 1. present. (Zhu et al. 2016, Character 346)

314. Obstantic margin of skull roof: 0. long; 1. short. (Zhu et al. 2016, Character 347)

***Note:*** *The obstantic margin is the overlap area of the skull roof between the trunk shield and the head shield (e.g. Miles 1971, Young 1984, Gardiner & Miles 1990)*

315. Postmarginal line issued from main lateral line: 0. on marginal or supratemporal; 1. on anterior paranuchal or tabular. (Zhu et al. 2016, Character 349)

316. Central sensory line: 0. absent; 1. present, (Zhu et al. 2016, Character 350)

317. Ventral lamina of opercular (submarginal) ossification: 0. absent; 1. present. (Zhu et al. 2016, Character 355)

318. Ventral lamina of suborbital (jugal): 0. absent; 1. present. (Zhu et al. 2016, Character 356)

319. Anterodorsal process of opercular (submarginal) ossification attaching onto skull: 0. absent; 1. present. (Zhu et al. 2016, Character 357)

320. Anterior median dorsal plate: 0. absent; 1. present, (Zhu et al. 2016, Character 358)

321. Anterior median dorsal plate (MD1) relative to posterior median dorsal plate (MD2) in length: 0. MD1 shorter than MD2; 1. MD1 longer than MD2. (Zhu et al. 2016, Character 361)

322. Anterior margin of unpaired anterior median dorsal plate: 0. broad; 1. pointed. (Zhu et al. 2016, Character 362)

323. Anterior lateral plate: 0. absent; 1. present. (Zhu et al. 2016, Character 363)

324. Postbranchial lamina of trunk armour: 0. lateral; 1 internal. (Zhu et al. 2016, Character 364)

325. Anteroventral plate: 0. absent; 1. present. (Zhu et al. 2016, Character 365)

326. Number of medioventral plates: 0. two; 1. one. (Zhu et al. 2016, Character 366)

327. Interolateral plate: 0. paired; 1. fused (unpaired semilunar plate). (Zhu et al. 2016, Character 367)

328. Anterior ventrolateral plates of both sides: 0. in contact; 1. separated. (Zhu et al. 2016, Character 368)

329. Brachial process: 0. absent; 1. present. (Zhu et al. 2016, Character 369)

330. Armoured pectoral appendage: 0. unjointed; 1. jointed. (Zhu et al. 2016, Character 371)

331. Sinus superior: 0. absent; 1. present.

***Note:*** This character is scored as inapplicable in taxa that lack a crus commune (c.279).

332. Pre-ampullary section of posterior semicircular canal: 0. underdeveloped to absent; 1. posterior circular canal forms a fuller ring.

***Note:*** *The ampulla of the posterior semicircular canal may be continuous with the vestibular cavity or can be separated from it by a portion of pre-ampullary canal. This section of canal can be short or extensive, and in some chondrichthyans can be very long. This character is adapted from Maisey, 2001, character 17.*

***New characters***

333. Ampullary end of anterior semicircular canal and external semicircular canal: 0. separated by the utricular chamber; 1. join before entering the utricular chamber.

***Note***: *This character is modified from Maisey, 2001a, character 25.*

334. Orientation of saccular cavity in anterior view: 0. flat inclined; 1. Steeply inclined or vertical.

***Note:*** *In most stem gnathostomes the laterally compressed saccular cavity is flat inclined in anterior view, rather than steeply inclined or vertical.*

335. Sacculus position: 0. restricted ventral to external semicircular canal; 1. extends dorsal to semicircular canal.

***Note*:** *This character considers whether the sacculus is dorsally extensive or is restricted ventrally relative to the external semicircular canal. In taxa that lack an external semicircular canal, the ampullae of the anterior and posterior semicircular canals are used as references.*

336. Vestibular cavity of the bony labyrinth shape: 0. drum-shaped; 1. irregularly shaped.

***Note:*** *In taxa such as Jagorina (Stensiö 1969, fig. 65), Kujdanowiaspis (Stensiö 1969, fig. 61A) and osteostracans (Stensiö 1927, figs. 18 & 19), the saccular cavity resembles a drum, or a flat cylinder, in that it is rounded in lateral view and compressed laterally. This contrasts with the condition in some ‘placoderms’ and crown gnathostomes, where the saccular cavity is bag-like and much more irregular in shape. It is unclear whether in Romundina the posteroventral bulge of the vestibular cavity is homologous to the lagena in crown gnathostomes, but even when this part is omitted, the saccular cavity in Romundina is still irregularly shaped in lateral view and does not resemble a drum.*

337. Endolymphatic complex shape: 0. simple and tube-like l; 1. differentiated into distinctive sections.

338. Endolymphatic complex position: 0. Lateral to the otic cartilaginous wall, close to inner ear; 1. Mesial to the cartilaginous wall, close to brain cavity.

339. Endolymphatic duct distal direction in lateral view: 0. posteriorly directed; 1. vertically directed.

340. Endolymphatic duct distal direction in coronal view: 0. posteriorly directed; 1. mesially directed; 2. laterally directed.

341. Nerve VIII bifurcates before entering the labyrinth cavity: 0. bifurcates; 1. does not bifurcate.

***Note****: this character is independent of the cartilaginous wall between the labyrinth and brain cavity, as Brindabellaspis have both a cartilaginous otic wall and the Nerve VIII does not bifurcate before entering the labyrinth cavity.*

342. Olfactory tracts: 0. parallel or near-parallel; 1. significantly diverged.

343. Prehypophysial diencephalon: 0. The prehypophysial ventral “step” is absent or insignificantly captured by endocast, the ventral aspect of telencephalon is continuous with the anterior boundary of the hypophysial recess; 1. significant prehypophysial diencephalon, indicated by a “step” between the optic nerve canal marking the start of the diencephalon, and the anterior boundary of the hypophysial recess.

344. Otic or pre-vagus section of myelencephalon: 0. Long, longer than metencephalon; 1. Short, shorter than metencephalon.

***Note*:** *Condition 0 is indicated by the otic section of myelencephalon (between Nerve VIII and X) longer than the metencephalon section, and condition 1 is indicated by the otic section of myelencephalon shorter than the metencephalon. We make clear distinction between the otic section of myelencephalon (anterior to Nerve X) and the craniospinal section (posterior to Nerve X) because the length of the craniospinal section is dependent on the numerical fluctuate of the occipital segments and is highly variable in closely associated taxa. As a result, only the otic section of myelencephalon is concerned here. The myelencephalon of Brindabellaspis have a moderately long craniospinal section, but the otic section is short compared to the condition in most other placoderms, and is shorter than the metencephalon section.*

***The following characters are taken from Coates et al. 2018:***

345. Postorbital process and arcade: 0. short and deep - width not more than maximum braincase width (excluding arcade); 1. process and arcade wide - width exceeds maximum width of braincase, and anteroposteriorly narrow; 2. process and arcade massive; 3. arcade forms postorbital pillar. (Coates et al. 2018, Character 144)

346. Postotic process: 0. absent; 1. present. (Coates et al. 2018, Character 165)

347. Space for forebrain and (at least) proximal portion of olfactory tracts narrow and elongate, extending between orbits: 0. absent; 1. present. (Coates et al. 2018, Character 118).

348. Roof of the endocranial space for telencephalon and olfactory tracts offset ventrally relative to level of mesencephalon: 0. absent; 1. present. (Coates et al. 2018, Character 171)

349. Trochlear nerve (IV) foramen anterior to optic nerve foramen: absent (0); present (1). (Coates et al. 2018, Character 126).

350. Angle of external semicircular canal: in lateral view, straight line projected through canal intersects anterior ampulla, external ampullae, and base of foramen magnum: absent (0); present (1). (Coates et al., 2018, Character 177)

351. Left and right external semicircular canals approach or meet the posterodorsal midine of the hindbrain roof: absent (0); present (1). (Coates et al., 2018, Character 178)

***Note:*** *Modified; contingent on whether crus commune is dorsal to endocranial roof.*

352. External opening for endolymphatic ducts anterior to crus commune: absent (0); present (1). (Coates et al. 2018, Character 186).

353. Perilymphatic fenestra within the endolymphatic fossa: absent (0); present (1). (Coates et al. 2018, Character 192).

354. Dentary marginal bone of mouth: absent (0); present (1). (Coates et al. 2018, Character 91).

355. First branchial arch meets neurocranium: 0 ventral to otic region; 1 posterior to otic region. (Dearden et al. 2019, character 68)

***The following characters are taken from Zhu et al. 2019:***

356. Dorsal articular lamina on trunk armour: 0 absent; 1 present (Zhu et al. 2019, character 3)

***Note:*** *The dorsal articular lamina on the trunk armour is present in the “reverse ginglymoid” type neck joints in antiarchs. However, the dual articulation in the dermal neck joints of Qilinyu and Silurolepis also has this character. To better illustrate the possible transition between complex types of dermal neck joints we use this and following new characters instead of the generalized types “sliding”, “ginglymoid” and “reverse-ginglymoid”.*

357. Cranial fossa receiving the ventral articular lamina of the trunk: 0 absent; 1 present (Zhu et al. 2019, character 4)

***Note****: This character separates the previously defined sliding-type joints to the ginglymoid joints, and avoiding the generalization in previous definitions. The sliding-type and reverse-ginglymoid type dermal neck joints lack the fossa receiving the articular lamina.*

358. Trunk ventral articular lamina develops into flange or condyle: 0 absent, continuous along the articular lamina of the anterior dorsolateral plate; 1 present, the articular lamina develops into distinctive flange or condyle. (Zhu et al. 2019, character 5)

***Note:*** *This character is previously mostly integrated into the “ginglymoid” category. However, sliding joint (absent of a cranial fossa) can also develops a distinct and protruding flange, in comparison with a continuous profile along the anterior dorsolateral plate.*

359. Rotatory contact of the articulation 0 absent; 1 present (Zhu et al. 2019, character 8)

***Note:*** *The rotatory dermal neck joint is only is only present in arthrodires above phlyctaenids (including brachythoracids).*

360. Lateral ridges on skull roof laterally defines the articulation: 0 absent; 1 present (Zhu et al. 2019, character 8)

***Note:*** *A lateral ridge or process (also called mesial articular process) defines the mesial boundary of the visceral contact face for the articular lamina on the paranuchal plate in petalichthyids and some ptyctodontids. This structure is absent in other placoderms.*

***New characters***:

361. T-shaped ethmoidal element: 0 absent; 1 present

362. Nerve opening penetrates T-shaped bone close to the midline: 0 absent; 1 present

363. Ventral fissure incorporates N. II opening: 0 absent; 1 present

364. Anterior dorsal fontanelle: 0 absent; 1 present

365. Cartilage comprises large cell spaces separated by extracellular mineralisation scaffold: 0. absent; 1 present

366. Lateral commissure invested in neurocranium: 0 present; 1 absent

367. Mineralised ring centra in pre-caudal vertebral column: 0 absent; 1 present

368. Endoskeletal radials in caudal fin: 0 absent; 1 present

**List of characters used in Analysis 4**

***Characters 5-69, 76-91, 93, 97, 103, 107-111, 113, 175-184, 186-201, 204-221, 225-227, 230-238, 241-253, 257, 260-263, 265, 269-271, 273-276, 280, 281, 283-300, 302-306, 310-330, 354, 356-360 deleted from our analysis 1 character list, and new characters 142–145 added.***

1. Tessellate prismatic calcified cartilage: 0. absent; 1. present.

2. Prismatic calcified cartilage: 0. single layered; 1. multi-layered.

***Note:*** *This character is contingent on c.1 (tesselate prismatic calcified cartilage) being coded as state 1.*

3. Perichondral bone: 0. present; 1. absent.

4. Extensive endochondral ossification: 0. absent; 1. present.

5. Gill arches: 0. largely restricted to region under braincase; 1. extend far posterior to braincase.

6. Basihyal: 0. absent; 1. present.

7. Interhyal: 0. absent; 1. present.

8. Hypohyal: 0. absent; 1. present.

9. Endoskeletal urohyal: 0. absent; 1. present.

10. Oral dermal tubercles borne on jaw cartilages or at margins of the mouth: 0. absent; 1. present.

11. Position of upper mandibular arch cartilage (and associated cheek plate where present): 0. entirely suborbital; 1. with a postorbital extension.

12. Autopalatine and quadrate: 0. comineralized; 1. separate mineralizations.

13. Large otic process of the palatoquadrate: 0. absent; 1. present.

14. Insertion area for jaw adductor muscles on palatoquadrate: 0. ventral or medial; 1. lateral.

15. Palatoquadrate fused with neurocranium: 0. absent; 1. present.

16. Oblique ridge or groove along medial face of palatoquadrate: 0. Absent; 1. present.

17. Fenestration of palatoquadrate at basipterygoid articulation: 0. absent; 1. present.

18. Perforate or fenestrate anterodorsal (metapterygoid) portion of palatoquadrate: 0. absent; 1. present.

19. Pronounced dorsal process on Meckelian bone or cartilage: 0. absent; 1. present.

20. Preglenoid process: 0. absent; 1. present.

21. Jaw articulation located on rearmost extremity of mandible: 0. absent; 1. present.

22. Precerebral fontanelle: 0. absent; 1. present.

23. Nasal opening(s): 0. dorsal, placed between orbits; 1. ventral and anterior to orbit.

24. Olfactory tracts: 0. short, with olfactory capsules situated close to telencephalon cavity; 1. elongate and tubular (much longer than wide).

25. Prominent pre-orbital rostral expansion of the neurocranium: 0. present; 1. absent

26. Pronounced sub-ethmoidal keel: 0. absent; 1. present.

27. Internasal vacuities: 0. absent; 1. present.

28. Discrete division of the ethmoid and more posterior braincase at the level of the optic tract canal: 0. absent; 1. present.

29. Position of myodome for superior oblique eye muscles: 0. posterior and dorsal to foramen for nerve II; 1. anterior and dorsal to foramen.

30. Endoskeletal intracranial joint: 0. absent; 1. present.

31. Spiracular groove on basicranial surface: 0. absent; 1. present.

32. Transverse otic process: 0. present; 1. absent.

33. Jugular canal: 0. long (invested in otic region along length of skeletal labyrinth); 1. short (restricted to short portion of region of skeletal labyrinth, or anterior to it); 2. absent (jugular vein uninvested in otic region).

34. Spiracular groove on lateral commissure: 0. absent; 1. present.

***Note:*** *This character is coded as inapplicable in taxa that lack a lateral commissure (postorbital process sensu Giles et al. 2015).*

35. Subpituitary fenestra: 0. absent; 1. present.

36. Supraorbital shelf broad with convex lateral margin: 0. absent; 1. present.

37. Orbit dorsal or facing dorsolaterally, surrounded laterally by endocranium: 0. present; 1. absent.

38. Eyestalk attachment area: 0. absent; 1. present.

39. Postorbital process: 0. absent; 1. present.

40. Canal for jugular in postorbital process: 0. absent; 1. present.

***Note:*** *This character is contingent on c.129 (postorbital process) being coded as state 1.*

41. Series of perforations for innervation of supraorbital sensory canal in supraorbital shelf: 0. absent; 1. present.

42. Extended prehypophysial portion of sphenoid: 0. absent; 1. present.

43. Narrow interorbital septum, with outer walls in contact along midline forming a single sheet: 0. absent; 1. present.

44. The main trunk of facial nerve (N. VII): 0. elongate and passes anterolaterally through orbital floor; 1. stout, divides within otic capsule at the level of the transverse otic wall.

45. Course of hyoid ramus of facial nerve (N. VII) relative to jugular canal: 0. traverses jugular canal, with separate exit in otic region; 1. intersects jugular canal, with exit through posterior jugular foramen.

***Note:*** *This character is coded as inapplicable in taxa where the jugular canal is uninvested in the otic region.*

46. Glossopharyngeal nerve (N. IX) exit: 0. foramen situated posteroventral to otic capsule and anterior to metotic fissure; 1. through metotic fissure.

***Note:*** *This character is coded as inapplicable in taxa that lack a metotic fissure.*

47. Relationship of cranial endocavity to basisphenoid: 0. endocavity occupies full depth of sphenoid; 1. endocavity dorsally restricted.

48. Subcranial ridges: 0. absent; 1. present.

49. Ascending basisphenoid pillar pierced by common internal carotid: 0. absent; 1. present.

50. Canal for lateral dorsal aorta within basicranial cartilage: 0. absent; 1. present.

51. Entrance of internal carotids: 0. through separate openings flanking the hypophyseal opening or recess; 1. through a common opening at the central midline of the basicranium.

52. Canal for efferent pseudobranchial artery within basicranial cartilage: 0. absent; 1. present.

53. Position of basal/basipterygoid articulation: 0. same anteroposterior level as hypophysial opening; 1. anterior to hypophysial opening; 2. posterior to hypophysial opening.

54. Articulation between neurocanium and palatoquadrate posterodorsal to orbit (suprapterygoid articulation): 0 absent; 1. present.

55. Labyrinth cavity: 0. separated from the main neurocranial cavity by a cartilaginous or ossified capsular wall; 1. skeletal capsular wall absent.

56. Basipterygoid process (basal articulation) with vertically oriented component: 0. absent; 1. present.

57. Pituitary vein canal: 0. dorsal to level of basipterygoid process; 1. flanked posteriorly by basipterygoid process.

58. External (horizontal) semicircular canal: 0. absent; 1. present.

59. External (horizontal) semicircular canal: 0. joins the vestibular region dorsal to posterior ampulla; 1. joins level with posterior ampulla.

60. Horizontal semicircular canal in dorsal view: 0. medial to path of jugular vein; 1. dorsal to jugular vein.

61. Lateral cranial canal: 0. absent; 1. present.

62. Posterior dorsal fontanelle: 0. absent; 1. present.

63. Shape of posterior dorsal fontanelle: 0. approximately as long as broad; 1. much longer than wide, slot-shaped.

***Note:*** *This character is contingent on c.153 (posterior dorsal fontanelle) being coded as state 1.*

64. Synotic tectum: 0 absent; 1 present.

65. Dorsal ridge: 0 absent; 1 present.

66. Shape of median dorsal ridge anterior to endolymphatic fossa: 0 developed as a squared-off ridge or otherwise ungrooved; 1 bears a midline groove.

***Note:*** *This character is contingent on c.155 (dorsal ridge) being coded as state 1.*

67. Position of hyomandibula articulation on neurocranium: 0. below or anterior to orbit, on ventrolateral angle of braincase; 1. on otic capsule, posterior to orbit.

68. Position of hyomandibula articulation relative to structure of skeletal labyrinth: 0. anterior or lateral to skeletal labyrinth; 1. at level of posterior semicircular canal.

***Note:*** *This character is coded as inapplicable in taxa where the hyomandibula articulates level with the orbit.*

69. Hyoid arch articulation on braincase: 0. single; 1. double.

70. Branchial ridges: 0. present; 1. reduced to vagal process; 2. absent (articulation made with bare cranial wall).

71. Craniospinal process: 0. absent; 1. present.

72. Ventral cranial fissure: 0. absent; 1. present.

73. Basicranial fenestra: 0. absent; 1. present.

74. Metotic (otic-occipital) fissure: 0. absent; 1. present.

75. Vestibular fontanelle: 0. absent; 1. present.

76. Occipital arch wedged in between otic capsules: 0. absent; 1. present.

77. Spino-occipital nerve foramina: 0. two or more, aligned horizontally; 1. one or two, dorsoventrally offset.

78. Ventral notch between parachordals: 0. present or entirely unfused; 1. absent.

79. Parachordal shape: 0. forming a broad, flat surface as wide as the otic capsules; 1. mediolaterally constricted relative to the otic capsules.

80. Stalk-shaped parachordal/occipital region: 0. absent; 1. present.

81. Paired occipital facets: 0. absent; 1. present.

82. Size of aperture to notochordal canal: 0. much smaller than foramen magnum; 1. as large, or larger, than foramen magnum.

83. Canal for median dorsal aorta within basicranium: 0. absent; 1. present.

84. Hypotic lamina (and dorsally directed glossopharyngeal canal): 0. absent; 1. present

85. Scapular process of shoulder endoskeleton: 0. absent; 1. present.

86. Pelvic fins: 0. absent; 1. present.

87. Pelvic claspers: 0. absent; 1. present.

88. Number of dorsal fins, if present: 0. one; 1. two.

***Note:*** *This character is coded as inapplicable in taxa that lack dorsal fins.*

89. Posterior dorsal fin shape: 0. base approximately as broad as tall, not broader than all of other median fins; 1. base much longer than the height of the fin, substantially longer than any of the other dorsal fins.

***Note:*** *This character is contingent on c.222 (number of dorsal fins) being coded as state 1 and this character is coded as inapplicable in taxa that lack dorsal fins.*

90. Basal plate in dorsal fin: 0. absent; 1. present.

***Note:*** *This character is coded as inapplicable in taxa that lack dorsal fins.*

91. Caudal radials: 0. extend beyond level of body wall and deep into hypochordal lobe; 1. radials restricted to axial lobe.

92. Supraneurals in axial lobe of caudal fin: 0. absent; 1. present.

93. Hyomandibula: 0. imperforate; 1. perforate.

94. Urohyal shape: 0. absent; 1. vertical plate.

95. Rostral tubuli: 0. absent; 1. present.

96. Position of anterior nostril: 0. facial; 1. at oral margin.

97. Posterior nostril: 0. facial; 1. at margin of oral cavity; 2 palatal.

98. Ventral face of nasal capsule in taxa with mineralized ethmoid: 0. complete; 1. fenestra ventrolateralis; 2. entire floor unmineralized.

99. Size of profundus canal in postnasal wall: 0. small; 1. large.

100. Relative position of jugular groove/canal and hyomandibular articulation: 0. hyomandibula dorsal; 1. hyomandibula ventral; 2. hyomandibula straddles.

101. Hypophyseal chamber: 0. projects posteroventrally; 1. projects ventrally or anteroventrally.

102. Crus commune of anterior and posterior semicircular canals: 0. dorsal to endocranial roof; 1. ventral to endocranial roof.

103. Horizontal semicircular canal: 0. obiquely oriented; 1. horizontally oriented.

104. Pelvic fin: 0. monobasal; 1. polybasal.

105. Endoskeletal lamina (postnasal wall) separating posterior nostril and orbit: 0. absent; 1. present.

106. Pituitary vein canal: 0. discontinuous, enters the cranial cavity; 1. discontinuous, enters hypophysial recess; 2. continuous transverse vein.

107. Crus commune: 0. absent; 1. present.

***Note:*** *A crus commune in the skeletal labyrinth is judged to be absent if the saccular cavity separates the anterior and posterior semicircular canals.*

***The following characters are taken from Zhu et al. 2016***

108. Location of pineal foramen/eminence: 0. level with posterior margin of orbits; 1. well posterior of orbits. (Zhu et al. 2016, Character 166)

109. Ethmoid articulation for palatoquadrate: 0. placed on postnasal wall; 1. extends posteriorly to the level of N.II. (Zhu et al. 2016, Character 240)

110. Foremost contact between palatoquadrate and braincase: 0. anterior to nasal cavity; 1. posterior to nasal cavity. (Zhu et al. 2016, Character 326)

111. Laterally extending palatoquadrate: 0. absent; 1. present. (Zhu et al. 2016, Character 327)

112. Close association of pineal organ and nasal cavitie: 0. absent; 1. present. (Zhu et al. 2016, Character 328)

113. Sinus superior: 0. absent; 1. present.

***Note:*** This character is scored as inapplicable in taxa that lack a crus commune (c.279).

114. Pre-ampullary section of posterior semicircular canal: 0. underdeveloped to absent; 1. posterior circular canal forms a fuller ring.

***Note:*** *The ampulla of the posterior semicircular canal may be continuous with the vestibular cavity or can be separated from it by a portion of pre-ampullary canal. This section of canal can be short or extensive, and in some chondrichthyans can be very long. This character is adapted from Maisey, 2001, character 17.*

***New characters***

115. Ampullary end of anterior semicircular canal and external semicircular canal: 0. separated by the utricular chamber; 1. join before entering the utricular chamber.

***Note***: *This character is modified from Maisey, 2001a, character 25.*

116. Orientation of saccular cavity in anterior view: 0. flat inclined; 1. Steeply inclined or vertical.

***Note:*** *In most stem gnathostomes the laterally compressed saccular cavity is flat inclined in anterior view, rather than steeply inclined or vertical.*

117. Sacculus position: 0. restricted ventral to external semicircular canal; 1. extends dorsal to semicircular canal.

***Note*:** *This character considers whether the sacculus is dorsally extensive or is restricted ventrally relative to the external semicircular canal. In taxa that lack an external semicircular canal, the ampullae of the anterior and posterior semicircular canals are used as references.*

118. Vestibular cavity of the bony labyrinth shape: 0. drum-shaped; 1. irregularly shaped.

***Note:*** *In taxa such as Jagorina (Stensiö 1969, fig. 65), Kujdanowiaspis (Stensiö 1969, fig. 61A) and osteostracans (Stensiö 1927, figs. 18 & 19), the saccular cavity resembles a drum, or a flat cylinder, in that it is rounded in lateral view and compressed laterally. This contrasts with the condition in some ‘placoderms’ and crown gnathostomes, where the saccular cavity is bag-like and much more irregular in shape. It is unclear whether in Romundina the posteroventral bulge of the vestibular cavity is homologous to the lagena in crown gnathostomes, but even when this part is omitted, the saccular cavity in Romundina is still irregularly shaped in lateral view and does not resemble a drum.*

119. Endolymphatic complex shape: 0. simple and tube-like l; 1. differentiated into distinctive sections.

120. Endolymphatic complex position: 0. Lateral to the otic cartilaginous wall, close to inner ear; 1. Mesial to the cartilaginous wall, close to brain cavity.

121. Endolymphatic duct distal direction in lateral view: 0. posteriorly directed; 1. vertically directed.

122. Endolymphatic duct distal direction in coronal view: 0. posteriorly directed; 1. mesially directed; 2. laterally directed.

123. Nerve VIII bifurcates before entering the labyrinth cavity: 0. bifurcates; 1. does not bifurcate.

***Note****: this character is independent of the cartilaginous wall between the labyrinth and brain cavity, as Brindabellaspis have both a cartilaginous otic wall and the Nerve VIII does not bifurcate before entering the labyrinth cavity.*

124. Olfactory tracts: 0. parallel or near-parallel; 1. significantly diverged.

125. Prehypophysial diencephalon: 0. The prehypophysial ventral “step” is absent or insignificantly captured by endocast, the ventral aspect of telencephalon is continuous with the anterior boundary of the hypophysial recess; 1. significant prehypophysial diencephalon, indicated by a “step” between the optic nerve canal marking the start of the diencephalon, and the anterior boundary of the hypophysial recess.

126. Otic or pre-vagus section of myelencephalon: 0. Long, longer than metencephalon; 1. Short, shorter than metencephalon.

***Note*:** *Condition 0 is indicated by the otic section of myelencephalon (between Nerve VIII and X) longer than the metencephalon section, and condition 1 is indicated by the otic section of myelencephalon shorter than the metencephalon. We make clear distinction between the otic section of myelencephalon (anterior to Nerve X) and the craniospinal section (posterior to Nerve X) because the length of the craniospinal section is dependent on the numerical fluctuate of the occipital segments and is highly variable in closely associated taxa. As a result, only the otic section of myelencephalon is concerned here. The myelencephalon of Brindabellaspis have a moderately long craniospinal section, but the otic section is short compared to the condition in most other placoderms, and is shorter than the metencephalon section.*

***The following characters are taken from Coates et al. 2018:***

127. Postorbital process and arcade: 0. short and deep - width not more than maximum braincase width (excluding arcade); 1. process and arcade wide - width exceeds maximum width of braincase, and anteroposteriorly narrow; 2. process and arcade massive; 3. arcade forms postorbital pillar. (Coates et al. 2018, Character 144)

128. Postotic process: 0. absent; 1. present. (Coates et al. 2018, Character 165)

129. Space for forebrain and (at least) proximal portion of olfactory tracts narrow and elongate, extending between orbits: 0. absent; 1. present. (Coates et al. 2018, Character 118).

130. Roof of the endocranial space for telencephalon and olfactory tracts offset ventrally relative to level of mesencephalon: 0. absent; 1. present. (Coates et al. 2018, Character 171)

131. Trochlear nerve (IV) foramen anterior to optic nerve foramen: absent (0); present (1). (Coates et al. 2018, Character 126).

132. Angle of external semicircular canal: in lateral view, straight line projected through canal intersects anterior ampulla, external ampullae, and base of foramen magnum: absent (0); present (1). (Coates et al., 2018, Character 177)

133. Left and right external semicircular canals approach or meet the posterodorsal midine of the hindbrain roof: absent (0); present (1). (Coates et al., 2018, Character 178)

***Note:*** *Modified; contingent on whether crus commune is dorsal to endocranial roof.*

134. External opening for endolymphatic ducts anterior to crus commune: absent (0); present (1). (Coates et al. 2018, Character 186).

135. Perilymphatic fenestra within the endolymphatic fossa: absent (0); present (1). (Coates et al. 2018, Character 192).

136. First branchial arch meets neurocranium: 0 ventral to otic region; 1 posterior to otic region. (Dearden et al. 2019, character 68)

***New characters***:

137. T-shaped ethmoidal element: 0 absent; 1 present

138. Nerve opening penetrates T-shaped bone close to the midline: 0 absent; 1 present

139. Ventral fissure incorporates N. II opening: 0 absent; 1 present

140. Anterior dorsal fontanelle: 0 absent; 1 present

141. Cartilage comprises large cell spaces separated by extracellular mineralisation scaffold: 0. absent; 1 present

142. Dermal scales: 0 present; 1 absent

143. Dermal plates: 0 present; 1 absent

144. Fin spines: 0 present; 1 absent

145. Teeth: 0 present; 1 absent

**Apomorphy list for the *Palaeospondylus*-Chondrichthyes sister group node**

Char 22. Precerebral fontanelle: 0. absent; 1. **present**.

Char 33. Jugular canal: 0. long (invested in otic region along length of skeletal labyrinth);

1. short (restricted to short portion of region of skeletal labyrinth, or anterior to it);

2. **absent** (jugular vein uninvested in otic region).

Char 68. Position of hyomandibula articulation relative to structure of skeletal labyrinth:

0. anterior or lateral to skeletal labyrinth; 1. **at level of posterior semicircular canal**.

Char 79. Parachordal shape: 0. **forming a broad, flat surface as wide as the otic capsules**;

1. mediolaterally constricted relative to the otic capsules

**VIII. Description of Supplementary Data and Video**

Supplementary Data are available at Figshare (<https://figshare.com/s/5315534e4deb2b0a5f9>e).

*File name: Supplementary Data 1*

Nexus file of the data matrix based on the Lu et al. (2017) dataset, used in the parsimony analysis with the proximal hyoid arch element identified as the hyomandibula.

*File name: Supplementary Data 2*

Nexus file of the data matrix based on the Lu et al. (2017) dataset, used in the parsimony analysis with the proximal hyoid arch element identified as the lateral commissure.

*File name: Supplementary Data 3*

Nexus file of the data matrix used in the parsimony analysis based on the Brownstein (2023) dataset.

*File name: Supplementary Data 4*

Nexus file of the data matrix based on the Lu et al. (2017) dataset, used in the parsimony analysis with the proximal hyoid arch element identified as the hyomandibula, with character list reduced to 145 characters.

*File name: Supplementary Video 1*

A video of the holotype of *Palaeospondylus australis* neurocranium QMF52826

**IX. Supplementary References**

Allis, E. P. 1923. The cranial anatomy of *Chlamydoselachus anguineus*. *Acta Zoologica, Stockholm* **4**, 123–221.

Bjerring, H. C. 1978. The “intracranial joint” versus the “ventral otic fissure”. *Acta Zoologica* **59**, 203–214.

Brazeau, M. D. & Friedman, M. 2014. The characters of Palaeozoic jawed vertebrates. *Zoological Journal of the Linnean Society* **170**, 779–821. doi:10.1111/zoj.12111.

Brazeau, M. D., Giles, S., Dearden, R. P., Jerve, A., Ariunchimeg, Y., Zorig, E., Sansom, R., Guillerme, T. & Castiello, M. 2020. Endochondral bone in an Early Devonian ‘placoderm’ from Mongolia. *Nature Ecology & Evolution* **4,** 1477–1484.

Brownstein, C. D. 2023. *Palaeospondylus* and the early evolution of gnathostomes. *Nature* **620**, E20-E22. doi:10.1038/s41586-023-06434-5

Bulman, O. M. B. 1931. Note on *Palaeospondylus gunni* Traquair. *Annals and Magazine of Natural History* **8**, 179–190.

Burrow, C. J. & Young, G. C. 2005. The acanthodian fauna of the Craven Peaks Beds (Early to Middle Devonian), western Queensland. *Memoirs of the Queensland Museum* **51**, 3–25.

Burrow, C. J. & Turner, S. 2012. Fossil fish taphonomy and the contribution of microfossils to documenting Devonian vertebrate history, p. 189–223 in *Earth and Life: Global biodiversity, extinction intervals and biogeographic perturbations through time* (ed. J.A. Talent). Springer’s Legacy Series, International Year of Planet Earth, Springer Science+Business Media B.V.

Burrow, C. J., Turner, S., Young, G. C. 2010. Middle Palaeozoic microvertebrate assemblages and biogeography of East Gondwana (Australasia, Antarctica). *Palaeoworld* **19**, 37–54. doi:10.1016/j.palwor.2009.11.001

Burrow, C. J., Young., G. C. & Lu., J. 2023. Dermal skeleton of the stem osteichthyan *Ligulalepis* from the Lower Devonian of New South Wales (Australia). *Spanish Journal of Palaeontology* **38**. doi:[10.7203/sjp.26575](https://doi.org/10.7203/sjp.26575)

Burrow, C., Young, G. & Senden, T. 2014. An antipodean *Palaeospondylus*? 2014 Australian Earth Sciences Convention (AESC). *Geological Society of Australia, Abstracts* **110**, 217–218.

Burrow, C. J., Hovestadt, D. C., Hovestadt-Mueler, M., Turner, S. & Young, G. C. 2008. New information on the Devonian shark *Mcmurdodus*, based on material from western Queensland, Australia. *Acta Geologica Polonica* **58**, 155–163.

Capetta H., Duffin C. & Zidek, J. 1993. Chondrichthyes, p. 593–609 in *The Fossil Record 2* (ed. M.J. Benton). Chapman & Hall, London.

Chang, M.-M. 1982. The braincase of *Youngolepis*, a Lower Devonian crossopterygian from Yunnan, south-western China. Doctoral thesis, Stockholm University, Stockholm, 113 pp.

Clement, A. M., King, B., Giles, S., Choo, B., Ahlberg, P. E., Young, G. C. & Long, J. A. 2018. Neurocranial anatomy of an enigmatic Early Devonian fish sheds light on early osteichthyan evolution. *eLife* **7**, 34349. doi:10.7554/eLife.34349

Coates, M. I. & Sequeira, S. E. K. 1998. The braincase of a primitive shark. *Transactions of the Royal Society of Edinburgh. Earth Sciences* **89**, 63–85.

Coates, M. I., Finarelli, J. A., Sansom, I. J., Andreev, P. S., Criswell, K. E., Tietjen, K., Rivers, M. L. & La Riviere, P. J. 2018. An early chondrichthyan and the evolutionary assembly of a shark body plan. *Proceedings of the Royal Society B: Biological Sciences*, **285**(1870), 20172418. 1-10. doi:10.1098/rspb.2017.2418

Davis, S. P., Finarelli, J. A. & Coates, M. I. 2012. *Acanthodes* and shark-like conditions in the last common ancestor of modern gnathostomes. *Nature* **486**, 247–250. doi:10.1038/nature11080

Dawson, J. W. 1893. Some salient points in the science of the Earth. London. 499 p.

Dean, B. 1898. Remarks on the affinities of *Palaeospondylus gunni*. In reply to Dr. R. H. Traquair. *Proceedings of the Zoological Society of London* **66**, 343–348.

Dean, B. 1900. The so-called Devonian lamprey, *Paleospondylus*. *Memoirs of the New York Academy of Sciences* **2**, 1–32.

Dearden, R. P., Stockey, C. & Brazeau, M. D. 2019. The pharynx of the stem-chondrichthyan *Ptomacanthus* and the early evolution of the gnathostome gill skeleton. *Nat Commun* **10**, 2050. doi:10.1038/s41467-019-10032-3

Dearden, R. P., den Blaauwen, J. L., Sansom, I. J., Burrow, C. J., Davidson, R., Newman, M. J., Ko, A. & Brazeau, M. D. 2021. A revision of *Vernicomacanthus* Miles with comments on the characters of stem-group chondrichthyans. *Papers in Palaeontology* **7**, 1949–1976. doi:10.1002/spp2.1369

de Beer, G. R. 1937. *The development of the vertebrate skull*. Oxford University Press. 554 pp., 143 pl.

Forey, P. L. & Gardiner, B. G. 1981. J.A. Moy-Thomas and his association with the British Museum (Natural History). *Bulletin of the British Museum of Natural History (Geology)* **35**, 131−144.

Friedman, M. & Brazeau, M. D. 2010. A reappraisal of the origin and basal radiation of the Osteichthyes. *Journal of Vertebrate Paleontology* **30**, 36–56. doi:10.1080/02724630903409071

Gardiner, B. G. 1984. The relationships of the palaeoniscoid fishes, a review based on new specimens of *Mimia* and *Moythomasia* from the Upper Devonian of Western Australia. *Bulletin of the British Museum (Natural History) Geology* **37**, 1–428.

Gardiner, B. G. & Bartram, A. W. H. 1977. The homologies of ventral cranial fissures in osteichthyans, p. 227–245 in *Problems in Vertebrate Evolution* (eds. Andrews, S. M., Miles, R. S. & Walker, A. D.), Academic Press, London.

Gardiner, B. G. & Miles, R. S. 1990. A new genus of eubrachythoracid arthrodire from Gogo, Western Australia. *Zool. J. Linn. Soc.* **99**, 159–204.

Giles, S., Darras, L., Clément, G., Blieck, A. & Friedman, M. 2015. An exceptionally preserved Late Devonian actinopterygian provides a new model for primitive cranial anatomy in ray-finned fishes. *Proceedings of the Royal Society of London B: Biological Sciences* **282**, 20151485. doi:10.1098/rspb.2015.1485

Gill, T. 1896. Fishes, Living and Fossil. *Science* **4**, 909–917.

Goodrich, E. S. 1930. *Studies on the structure and development of vertebrates*. London, Macmillan (Dover Publications, New York 1958), 837 pp.

Goujet, D. & Young, G. C. 2004. Placoderm anatomy and phylogeny: new insights, p. 109–126 in *Recent Advances in the Origin and Early Radiation of Vertebrates*, (ed. Arratia, G., Wilson, M. V. H. & Cloutier, R.), München, Verlag Dr. Friedrich Pfeil.

Hirasawa, T. & Kuratani, S., 2023. Reply to: *Palaeospondylus* and the early evolution of gnathostomes. Nature **620**, E23-E24. doi:10.1038/s41586-023-06435-4

Hirasawa, T., Oisi, Y. & Kuratani, S. 2016. *Palaeospondylus* as a primitive hagfish. *Zoological Letters* **2**, 20, doi:10.1186/s40851-016-0057-0.

Hirasawa, T., Hu, Y., Uesugi, K., Hoshino, M., Manabe, M. & Kuratani, S. 2022. Morphology of *Palaeospondylus* shows affinity to tetrapod ancestors. *Nature* **606**, 109–112. doi:10.1038/s41586-022-04781-3

Holland, T. 2014. The endocranial anatomy of *Gogonasus andrewsae* Long, 1985 revealed through micro CT-scanning. *Earth and Environmental Science Transactions of the Royal Society of Edinburgh* **105**, 9–34. doi:10.1017/S1755691014000164

Holmgren, N. 1940. Embryological, morphological, and phylogenetical researches. *Acta Zoologica* **21**, 51-266. doi:10.1111/j.1463-6395.1940.tb00339.x

Hu, Y., Lu, J.& Young, G. C. 2017. New findings in a 400 million-year-old Devonian placoderm shed light on jaw structure and function in basal gnathostomes. *Scientific Reports* **7**, 7813. doi:10.1038/s41598-017-07674-y

Janvier, P. 1996. *Early Vertebrates*. Clarendon Press, Oxford, 393 pp.

Jarvik, E. 1996. The Devonian tetrapod Ichthyostega. *Fossils and Strata* **40**, 1–206.

Johanson, Z., Kearsley, A., den Blaauwen, J., Newman, M. & Smith, M. M. 2010. No bones about it: An enigmatic Devonian fossil reveals a new skeletal framework - A potential role of loss of gene regulation. *Seminars in Cell and Developmental Biology* **21**, 414–423. doi:10.1016/j.semcdb.2009.10.011

Johanson, Z., Kearsley, A., den Blaauwen, J., Newman, M. J. & Smith, M. M. 2012. Ontogenetic development of an exceptionally preserved Devonian cartilagenous skeleton. *Journal of Experimental Zoology Part B, Molecular and Developmental Evolution* **318**, 50–58. doi:10.1002/jez.b.21441

Johanson, Z., Smith, M., Sanchez, S., Senden, T., Trinajstic, K. & Pfaff. C. 2017. Questioning hagfish affinities of the enigmatic Devonian vertebrate *Palaeospondylus*. *Royal Society Open Science* **4**, 170214. doi:10.1098/rsos.170214

Kerr, J. G. 1900. The zoological position of' *Palaeospondylus*, Traquair. *Proceedings of the Cambridge Philosophical Society* **10**, 298–299.

Kyle, H. 1926. *The biology of fishes*. London, New York. 396 p.

Long, J.A., Young, G.C., Holland, T., Senden, T.J. & Fitzgerald, E.M.G. 2006. An exceptional Devonian fish from Australia sheds light on tetrapod origins. *Nature* **444***,* 199–202.

Long, J. A., Thomson, V., Burrow, C. J. & Turner, S. 2021. Fossil chondrichthyan remains from the Middle Devonian Kevington Creek Formation, South Blue Range, Victoria, p. 239–245 in *Ancient Fishes and their Living Relatives: a Tribute to John G. Maisey* (eds. Pradel, A., Denton, J. S. S. & Janvier, P.). Verlag Dr Friedrich Pfeil, Munich.

Lu, J., Zhu, M., Long, J.A., Zhao, W., Senden, T.J., Jia, L. & Qiao, T. 2012. The earliest known stem-tetrapod from the Lower Devonian of China. *Nature Communications*, **3**: 1160. doi: 10.1038/ncomms2170

Lu, J., Giles, S., Friedman, M. & Zhu, M. 2017. A new stem sarcopterygian illuminates patterns of character evolution in early bony fishes. *Nature Communications* **8**, 8 p, doi:10.1038/s41467-017-01801-z

Lu, J., Young, G., Hu, Y. Z., Qiao, T. & Min, Z. 2019. The posterior cranial portion of the earliest known Tetrapodomorph *Tungsenia paradoxa* and the early evolution of tetrapodomorph endocrania. *Vertebrata PalAsiatica* **57**, 93-104, doi:10.19615/j.cnki.1000-3118.181031

Maisey, J. G. 1989. Visceral skeleton and musculature of a Late Devonian shark. *Journal of Vertebrate Paleontology* **9**, 174–190.

Maisey, J. G. 2001a. Remarks on the inner ear of elasmobranchs and its interpretation from skeletal labyrinth morphology*. Journal of Morphology* **250**, 236–264.

Maisey, J. G. 2001b. A primitive chondrichthyan braincase from the Middle Devonian of Bolivia, p. 263–288 in *Major Events in Early Vertebrate Evolution* (ed. Ahlberg, P. E.), Taylor & Francis, London & New York.

Maisey, J. G. 2005. Braincase of the Upper Devonian shark *Cladodoides wildungensis* (Chondrichthyes, Elasmobranchii), with observations on the braincase in early chondrichthyans. *Bulletin of the American Museum of Natural History* **288**, 1–103.

Maisey, J. G. 2007. The braincase in Paleozoic symmoriiform and cladoselachian sharks. *Bulletin of the American Museum of Natural History* **307**, 1–122.

Maisey, J. G. & Anderson, M. E. 2001. A primitive chondrichthyan braincase from the Early Devonian of South Africa. *Journal of Vertebrate Paleontology* **21**, 702–713.

Maisey, J., Miller, R. &, Turner, S. 2009. The braincase of the chondrichthyan *Doliodus* from the Lower Devonian Campbellton Formation of New Brunswick, Canada. *Acta Zoologica* **90** (Suppl. 1), 109–122.

Maisey, J. G., Janvier, P., Pradel, A., Denton, J. S. S., Bronson, A., Miller, R. & Burrow, C. J. 2019. *Doliodus* and pucapampellids: contrasting perspectives on stem chondrichthyan morphology, p. 87-109 in *Evolution and Development of Fishes* (eds. Underwood, C., Richter, M. & Johanson, Z.). Cambridge University Press, Cambridge.

Mark-Kurik, E. & Poldvere, A. 2012. Devonian stratigraphy in Estonia: current state and problems. *Estonian Journal of Earth Sciences* **61**, 33–47. doi:10.3176/earth.2012.1.03

Marshall J. E. A., Rogers, D. A. & Whiteley M. J. 1996. Devonian marine incursions into the Orcadian Basin, Scotland. *Journal of the Geological Society, London* **153**, 451–466.

Marshall J. E. A., Astin T. R., Brown J. F., Mark-Kurik E. & Lazauskiene J. 2007. Recognizing the Kačák Event in the Devonian terrestrial environment and its implications for understanding land – sea interactions. In *Devonian Events and Correlations* (eds. Becker, R. T. & Kirchgasser, W. T.), *Geological Society, London, Special Publications* **278**, 133–155.

Miles, R. S. 1971. The Holonematidae (placoderm fishes), a review based on new specimens of *Holonema* from the Upper Devonian of Western Australia. *Philosophical Transactions of the Royal Society of London (B)* **263**, 101–234.

Moy-Thomas, J. A. 1940. The Devonian fish *Palaeospondylus gunni* Traquair. *Philosophical Transactions of the Royal Society of London, Series B, Biological Sciences* **230**, 391–413.

Newman, M. J. & den Blaauwen, J. L. 2008. New information on the enigmatic Devonian vertebrate *Palaeospondylus gunni*. *Scottish Journal of Geology* **44**, 89–91.

Newman, M. J., Davidson, R. G., Blaauwen, J. L. D. & Burrow, C. J. 2012. The Early Devonian acanthodian *Uraniacanthus curtus* (Powrie, 1870) n. comb. from the Midland Valley of Scotland. *Geodiversitas* **34**, 739–759. doi:10.5252/g2012n4a2

Pradel, A., Maisey, J. G., Tafforeau, P. & Janvier, P. 2009. An enigmatic gnathostome vertebrate skull from the Middle Devonian of Bolivia. *Acta Zoologica* (Stockholm) **90** (Suppl. 1), 123-133.

Pradel, A., Maisey, J. G., Tafforeau, P., Mapes, R. H. & Mallatt, J. 2014. A Palaeozoic shark with osteichthyan-like branchial arches. *Nature* **509**, 608–611. doi:10.1038/nature13195

Rahmat, S. & Gilland, E. 2014. Comparative anatomy of the carotid-basilar arterial trunk and hindbrain penetrating arteries in vertebrates. *The Open Anatomy Journal* **6**, 1–26. doi:10.2174/1877609401406010001

Ritchie, A. 2004. A new genus and two new species of groenlandaspidid arthrodire (Pisces: Placodermi) from the Early-Middle Devonian Mulga Downs Group of western New South Wales, Australia. In Proceedings of Symposium 6 (Palaeozoic Vertebrates): First International Palaeontological Congress (IPC 2002), Sydney, Australia, July 2002 (ed. Young, G. C.). *Fossils and Strata* **50**, 56–81.

Schaeffer, B. 1981. The xenacanth shark neurocranium, with comments on elasmobranch monophyly. *Bulletin of the America Museum of Natural History* **169**, 1–66.

Schultze, H. P. 1986. Dipnoans as sarcopterygians. In: Biology and evolution of lungfishes *Journal of Morphology* **190**(SI), 39–74.

Sollas, W. J. & Sollas, I. B. J. 1904. An account of the Devonian fish *Palaeospondylus gunni* Traquair. *Philosophical Transactions of the Royal Society, London* **196**, 267–294.

Stensiö, E. A. 1927. The Devonian and Downtonian vertebrates of Spitzbergen. 1. Family Cephalaspidae. *Skrifter om Svalbard og Ishavet* **12**, 1–391.

Stensiö, E. 1969. *Traité de Paléontologie* Vol. 4:2 (ed. Piveteau, J.), p. 71–692. Masson, Paris.

Stephenson, M. H., Leng, M. J., Michie, U. & Vane, C. H. 2006. Palaeolimnology of Palaeozoic lakes, focussing on a single lake cycle in the Middle Devonian of the Orcadian Basin, Scotland. *Earth Science Reviews* **75**, 177–197.

Thomson, K. S., Sutton, M. & Thomas, B. 2003. A larval Devonian lungfish. 2003. *Nature* **426**, 833–834.

Traquair, R. H. 1890. On the fossil fishes found at Achanarras Quarry, Caithness. *Annals and Magazine of Natural History* **1890**, 479–486.

Traquair, R. H. 1897. Affinities of *Palaeospondylus gunni,* Traquair. *Proceedings of the Zoological Society* **1897**, 314–317.

Trewin N. H. 1986. Palaeoecology and sedimentology of the Achanarras fish bed of the Middle Old Red Sandstone, Scotland. *Transactions of the Royal Society of Edinburgh, Earth Sciences* **77**, 21–46.

Turner, S. 1995. Devonian thelodont scales (Agnatha, Thelodonti) from Queensland. *Memoirs of the Queensland Museum* **38**, 677–685.

Turner, S., 1997. Sequence of Devonian thelodont scale assemblages in East Gondwana. *Geological Society of America, Special Publications* **32**, 295–315.

Turner, S., Jones, P. J., Draper, J. J., 1981. Early Devonian thelodonts (Agnatha) from the Toko Syncline, western Queensland, and a review of other Australian discoveries. *BMR Journal of Australian Geology and Geophysics* **6**, 51–69.

Turner, S. & Young, G. C. 1987. Shark teeth from the Early-Middle Devonian Cravens Peak Beds, Georgina Basin, Queensland. *Alcheringa* **11**, 233–244.

Wang, S. Q. 1983. Ostracods from the Devonian Sipai Formation of Guangxi. *Memoir of the Nanjing Institute of Geology and Palaeontology* **18**, 111-154 (in Chinese, with English abstract).

Young, G. C. 1979. New information on the structure and relationships of *Buchanosteus* (Placodermi, Euarthrodira) from the Early Devonian of New South Wales. *Zool. J. Linn. Soc.* **66**, 309-352.

Young, G. C. 1984. An asterolepidoid antiarch (placoderm fish) from the Early Devonian of the Georgina Basin, central Australia. *Alcheringa* **8**, 65–80.

Young, G. C. 1986. The relationships of placoderm fishes. *Zoological Journal of the Linnean Society* **88**, 1–57.

Young, G. C. 1991. The first armoured agnathan vertebrates from the Devonian of Australia, p. 67–85 in *Early Vertebrates and Related Problems of Evolutionary Biology* (eds. Chang, M. M., Liu, Y. H., Zhang, G. R.). Science Press, Beijing.

Young, G. C. 1996. Devonian (chart 4). p. 96–109 in *An Australian Phanerozoic Timescale* (eds. Young, G. C. & Laurie, J. R.). Oxford University Press, Melbourne.

Young, G. C. & Goujet, D. 2003. Devonian fish remains from the Dulcie Sandstone and Cravens Peak Beds, Georgina Basin, central Australia. *Records of the Western Australian Museum, Supplement* **65**, 1–85.

Young, G. C. & Turner, S. 2000. Devonian microvertebrates and marine-nonmarine correlation in East Gondwana: Overview, in Palaeozoic Vertebrate Biochronology and Global Marine/Non-Marine Correlation. Final Report of IGCP 328 (1991-1996) (eds. Blieck, A. & Turner, S.). *Courier Forschungsinstitut Senckenberg* **223**, 453–470.

Young, G. C., Burrow, C. B., Long, J. A., Turner, S. & Choo, B. 2010. Devonian macrovertebrate assemblages and biogeography of East Gondwana (Australasia, Antarctica). *Palaeoworld* **19**, 55–74. doi:10.1016/j.palwor.2009.11.005

Young, G. C. & Lu, J. 2020. Asia–Gondwana connections indicated by Devonian fishes from Australia: palaeogeographic considerations. *Journal of Palaeogeography* **9**, 8. doi:10.1186/s42501-020-00057-x

Young, G. C. & Schultze, H.-P. 2005. New osteichthyans (bony fishes) from the Devonian of central Australia. *Mitteilungen aus dem Museum fur Naturkunde in Berlin, Geowissenschaftliche Reihe* **8**, 13–35.

Zhu, M. 2000. Catalogue of Devonian vertebrates in China, with notes on bio-events, in Palaeozoic Vertebrate Biochronology and Global Marine/Non-Marine Correlation. Final Report of IGCP 328 (1991-1996) (eds. Blieck, A. & Turner, S.). *Courier Forschungsinstitut Senckenberg* **223**, 373–390.

Zhu, M., Ahlberg, P.E., Pan, Z., Zhu, Y., Qiao, T., Zhao, W., Jia, L. & Lu, J. 2016. A Silurian maxillate placoderm illuminates jaw evolution. Science **354**, 334–336. doi:10.1126/science.aah3764

Zhu, M., Wang, W., Yu, X., 2010. *Meemannia eos*, a basal sarcopterygian fish from the Lower Devonian of China – expanded description and significance, pp. 199-214 in *Morphology, Phylogeny and Paleobiogeography of Fossil Fishes* (eds. Elliott, D.K., Maisey, J.G., Yu, X. & Miao, D.). Verlag Dr. Friedrich Pfeil, Munich.

Zhu, M., Zhao, W. J., Jia, L. T., Lu, J., Qiao, T. & Qu, Q. M. 2009. The oldest articulated osteichthyan reveals mosaic gnathostome characters. *Nature* **458**, 469–474. doi:10.1038/nature07855

Zhu, Y.-A., Lu, J. & Zhu, M. 2019. Reappraisal of the Silurian placoderm *Silurolepis* and insights into the dermal neck joint evolution. *Royal Society Open Science* **6**, 191181. doi:10.1098/rsos.191181
